# Supplementary material for: Incidence of sudden cardiac death in the young: a systematic review
Source: BMJ Open. 2020 Oct 7;10(10):e040815. doi: 10.1136/bmjopen-2020-040815 (PMC7542928; doi:10.1136/bmjopen-2020-040815)
Supplement: Supplementary data [file bmjopen-2020-040815supp001.pdf]

Incidence of sudden cardiac death in the young: a systematic review- Supplementary material

Contents:

MEDLINE search strategy- page 1

Table S1: study risk of bias assessment- page 2

Figure S1: Sudden cardiac death incidence by age- page 3

Figure S2: Sudden cardiac death incidence by athletic/ military status- page 4

Figure S3: Sudden cardiac death incidence by race- page 5

MEDLINE search strategy

1 exp Death, Sudden, Cardiac/
2 (sudden adj3 death).mp
3 cardiac death.mp
4 1 or 2 or 3
5 exp Incidence/
6 incidence.mp
7 exp Prevalence/
8 prevalence.mp
9 5 or 6 or 7 or 8
10 (adolescen* or young* or student* or athlet* or soccer or football*).mp
11 exp Young Adult/ or exp Adult/
12 exp Adolescent/
13 exp Child/
14 10 or 11 or 12 or 13
15 exp Cohort Studies/
16 exp Longitudinal Studies/
17 exp Follow-Up Studies/
18 exp Prospective Studies/
19 exp Retrospective Studies/
20 (longitudinal or prospective* or observational or registry).mp
21 (autopsy or death certificate*).mp
22 15 or 16 or 17 or 18 or 19 or 20 or 21
23 4 and 9 and 14 and 22
24 limit 23 to (case reports or comment or editorial or letter)
25 23 not 24

Table S1: study risk of bias assessment

	1-Appropriate sample frame	2-Sampling of participants	3-Adequate sample size	4-Description of subjects and setting	5-Sufficient coverage of sample in analysis	6-Valid methods to identify condition	7-Measurement of condition-standardised and reliable	8-Appropriate statistical analysis	9-Response rate adequate
Anastasakis 2018	Y	Y	Y	Y	Y	Y	Y	Y	Y
Anderson 1994	Y	Y	Y	Y	Y	N	Y	Y	Y
Asatryan 2017	Y	Y	Y	Y	Y	N	N	N	N
Bagnall 2016	Y	Y	Y	Y	Y	Y	Y	Y	Y
Bonny 2017	Y	N	N	N	U/C	N	Y	N	Y
Chugh 2004	Y	Y	N	Y	Y	N	N	N	Y
Corrado 2006	Y	Y	Y	Y	N	N	Y	Y	Y
Drehner 1999	N	Y	Y	Y	Y	N	U/C	N	N
Driscoll 1985	Y	Y	N	Y	Y	N	N	Y	Y
Eckart 2004	N	Y	Y	Y	Y	N	Y	Y	Y
Eckart 2011	N	Y	Y	Y	Y	N	Y	Y	N
Einarsson 2007	Y	Y	Y	Y	Y	U/C	U/C	U/C	U/C
El-Assaad 2017	Y	Y	Y	Y	Y	N	Y	N	Y
Fragkouli 2010	Y	Y	Y	Y	U/C	N	Y	N	U/C
Goudevenos 1995	N	Y	N	Y	Y	N	N	N	N
Harmon 2016	N	Y	Y	Y	N	N	N	Y	N
Harmon 2015	N	Y	Y	Y	N	N	N	Y	U/C
Hofer 2014	Y	Y	Y	Y	Y	N	N	Y	Y
Hua 2009	Y	N	N	N	N	N	N	Y	Y
Karvouni 2000	Y	Y	Y	Y	U/C	N	Y	N	U/C
Malhotra 2018	N	Y	N	Y	Y	U/C	Y	Y	Y
Margey 2011	Y	Y	Y	Y	Y	Y	Y	Y	N
Maron 2016	Y	Y	Y	Y	Y	U/C	U/C	Y	Y
Maron 2014	N	Y	Y	Y	N	U/C	Y	Y	N
Morentin 2001	Y	Y	Y	Y	Y	N	Y	Y	Y
Morentin 2011	N	Y	N	Y	Y	Y	U/C	N	Y
Neuspiel 1985	Y	Y	Y	Y	Y	N	N	N	Y
Papadakis 2009	Y	Y	Y	Y	Y	N	Y	Y	Y
Perez 1992	Y	Y	N	Y	Y	N	U/C	N	Y
Pilmer 2013	Y	Y	Y	Y	U/C	N	Y	N	U/C
Pilmer 2014	Y	Y	Y	Y	Y	N	Y	N	Y
Uuskula 1998	N	Y	Y	Y	Y	N	U/C	Y	U/C
Vaartjes 2009	Y	Y	Y	Y	Y	N	Y	Y	Y
Winkel 2017	Y	Y	Y	Y	Y	N	N	Y	Y
Wisten 2002	Y	Y	Y	Y	Y	Y	N	N	Y
Wisten 2017	Y	Y	Y	Y	Y	N	N	Y	N
Wren 2000	Y	Y	Y	Y	Y	N	Y	Y	Y
Zhang 2019	Y	N	Y	N	Y	N	Y	N	Y

Key: Y- Yes; N- No; U/C- Unclear

Full list of questions:

1. Was the sample frame appropriate to address the target population?
2. Were study participants sampled in an appropriate way?
3. Was the sample size adequate?
4. Were the study subjects and the setting described in detail?
5. Was the data analysis conducted with sufficient coverage of the identified sample?
6. Were valid methods used for the identification of the condition?
7. Was the condition measured in a standard, reliable way for all participants?
8. Was there appropriate statistical analysis?
9. Was the response rate adequate, and if not, was the low response rate managed appropriately?

Couper K, *et al. BMJ Open* 2020; 10:e040815. doi: 10.1136/bmjopen-2020-040815

Figure S1: Sudden cardiac death incidence by age

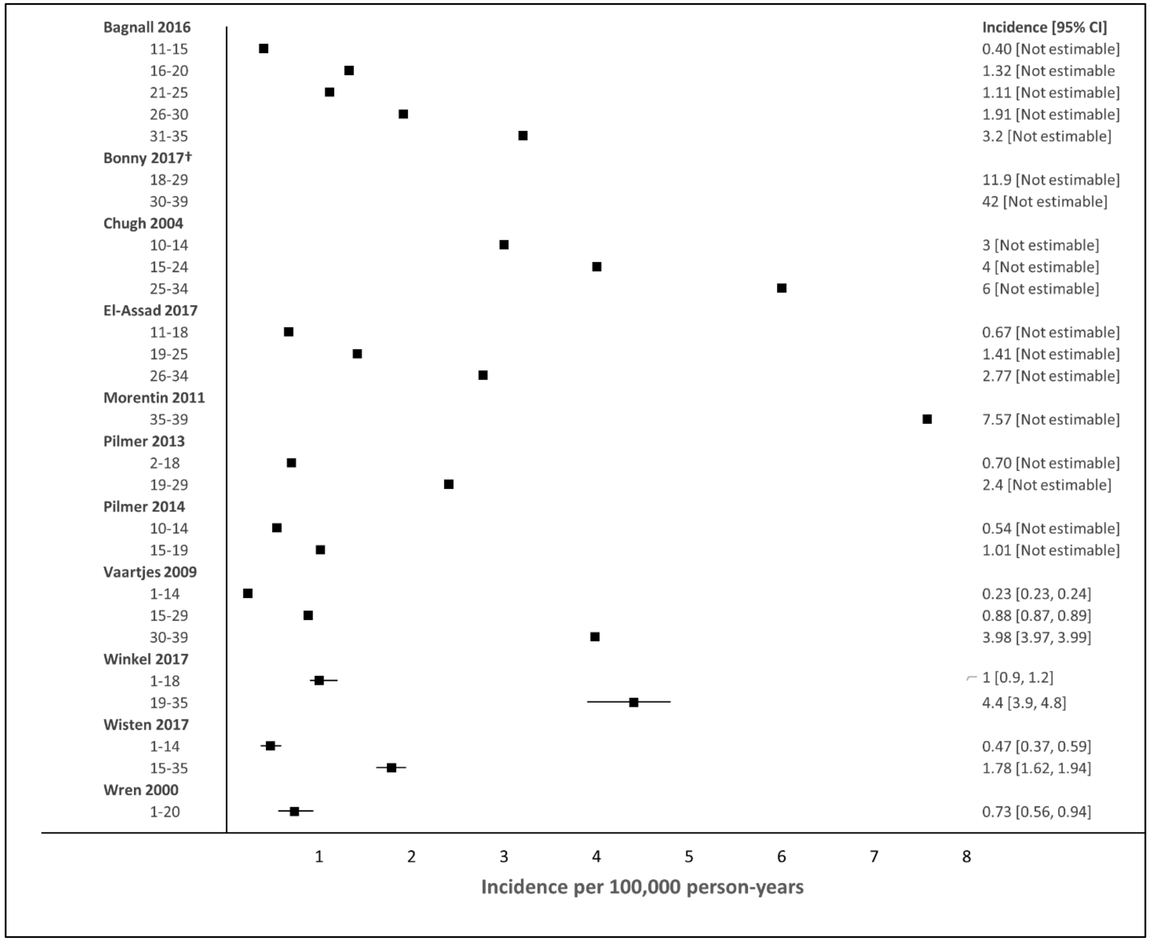

†- Data not presented on chart as significant outlier

Figure S2: Sudden cardiac death incidence by athletic/ military status

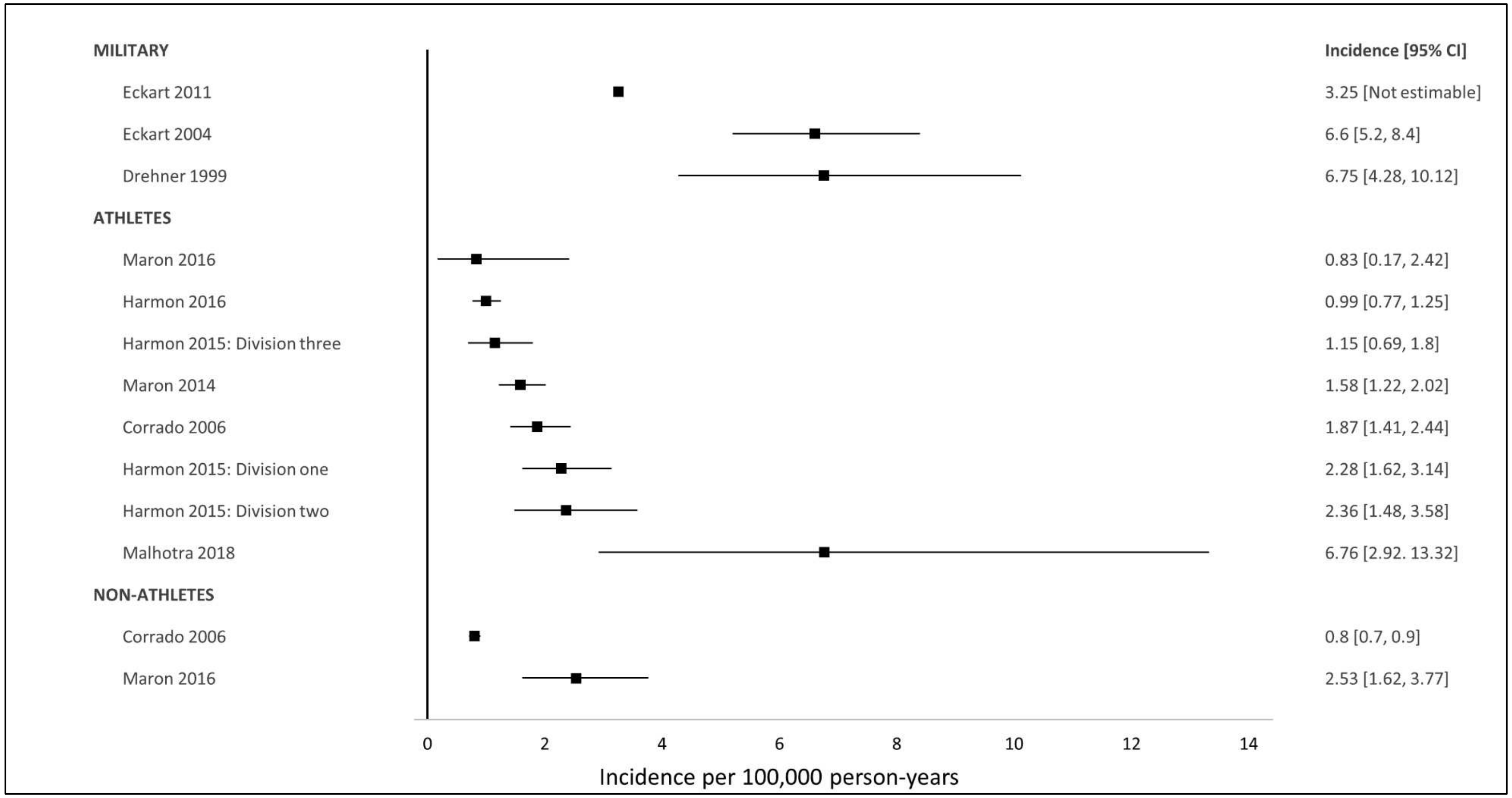

Figure S3: Sudden cardiac death incidence by race

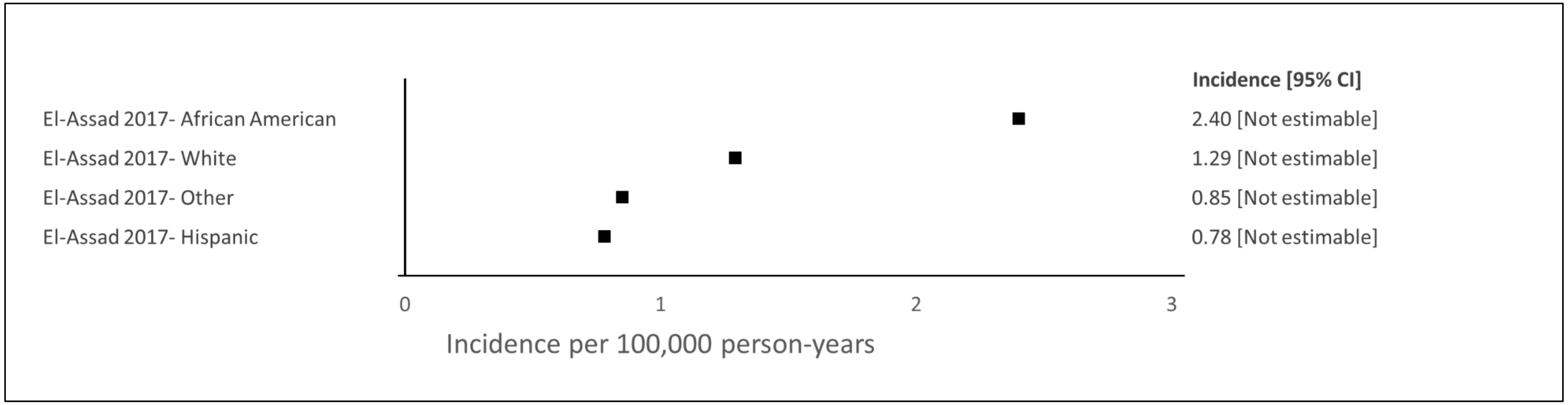

Couper K, *et al. BMJ Open* 2020; 10:e040815. doi: 10.1136/bmjopen-2020-040815
