# Supplementary material for: Incidence of sudden cardiac death in the young: a systematic review
Source: BMJ Open. 2020 Oct 7;10(10):e040815. doi: 10.1136/bmjopen-2020-040815 (PMC7542928; doi:10.1136/bmjopen-2020-040815)
Supplement: Author's manuscript [file bmjopen-2020-040815.draft_revisions.pdf]

BMJ Open

BMJ Open is committed to open peer review. As part of this commitment we make the peer review history of every article we publish publicly available.

When an article is published we post the peer reviewers' comments and the authors' responses online. We also post the versions of the paper that were used during peer review. These are the versions that the peer review comments apply to.

The versions of the paper that follow are the versions that were submitted during the peer review process. They are not the versions of record or the final published versions. They should not be cited or distributed as the published version of this manuscript.

BMJ Open is an open access journal and the full, final, typeset and author-corrected version of record of the manuscript is available on our site with no access controls, subscription charges or pay-per-view fees (http://bmjopen.bmj.com).

If you have any questions on BMJ Open's open peer review process please email info.bmjopen@bmj.com

BMJ Open

Incidence of sudden cardiac death in the young: a systematic review

Journal:	*BMJ Open*
Manuscript ID	bmjopen-2020-040815
Article Type:	Original research
Date Submitted by the Author:	22-May-2020
Complete List of Authors:	Couper, Keith; University of Warwick Warwick Medical School, Warwick Medical School; University Hospitals Birmingham NHS Foundation Trust, Critical Care Unit<br>Putt, Oliver; University of Warwick Warwick Medical School, Warwick Medical School<br>Field, Richard; University Hospitals Birmingham NHS Foundation Trust, Critical Care Unit<br>Poole, Kurtis; University of Warwick Warwick Medical School, Warwick Medical School<br>Bradlow, William; University Hospitals Birmingham NHS Foundation Trust, Department of cardiology<br>Clarke, Aileen; University of Warwick Warwick Medical School, Warwick Medical School<br>Perkins, Gavin; University of Warwick Warwick Medical School, Warwick Medical School; University Hospitals Birmingham NHS Foundation Trust, Critical Care Unit<br>Royle, Pamela; University of Warwick Warwick Medical School, Warwick Medical School<br>Yeung, Joyce ; University of Warwick Warwick Medical School, Warwick Medical School; University Hospitals Birmingham NHS Foundation Trust, Critical Care Unit<br>Taylor-Phillips, Sian; University of Warwick Warwick Medical School, Warwick Medical School
Keywords:	Cardiac Epidemiology < CARDIOLOGY, EPIDEMIOLOGY, Adult cardiology < CARDIOLOGY, Paediatric cardiology < PAEDIATRICS

SCHOLARONE™
Manuscripts

I, the Submitting Author has the right to grant and does grant on behalf of all authors of the Work (as defined in the below author licence), an exclusive licence and/or a non-exclusive licence for contributions from authors who are: i) UK Crown employees; ii) where BMJ has agreed a CC-BY licence shall apply, and/or iii) in accordance with the terms applicable for US Federal Government officers or employees acting as part of their official duties; on a worldwide, perpetual, irrevocable, royalty-free basis to BMJ Publishing Group Ltd ("BMJ") its licensees and where the relevant Journal is co-owned by BMJ to the co-owners of the Journal, to publish the Work in this journal and any other BMJ products and to exploit all rights, as set out in our licence.

The Submitting Author accepts and understands that any supply made under these terms is made by BMJ to the Submitting Author unless you are acting as an employee on behalf of your employer or a postgraduate student of an affiliated institution which is paying any applicable article publishing charge ("APC") for Open Access articles. Where the Submitting Author wishes to make the Work available on an Open Access basis (and intends to pay the relevant APC), the terms of reuse of such Open Access shall be governed by a Creative Commons licence – details of these licences and which Creative Commons licence will apply to this Work are set out in our licence referred to above.

Other than as permitted in any relevant BMJ Author's Self Archiving Policies, I confirm this Work has not been accepted for publication elsewhere, is not being considered for publication elsewhere and does not duplicate material already published. I confirm all authors consent to publication of this Work and authorise the granting of this licence.

For peer review only

Incidence of sudden cardiac death in the young: a systematic review

Keith Couper, Oliver Putt, Richard Field, Kurtis Poole, William Bradlow, Aileen Clarke, Gavin D Perkins, Pamela Royle, Joyce Yeung, Sian Taylor-Phillips

Keith Couper, Assistant Professor- k.couper@warwick.ac.uk
Warwick Medical School, University of Warwick, Coventry, UK
Critical Care Unit, University Hospitals Birmingham NHS Foundation Trust, Birmingham, UK

Oliver Putt, Academic Foundation Doctor- oliver.putt@nhs.net
Warwick Medical School, University of Warwick, Coventry, UK

Richard Field, Registrar- richiefield@doctors.org.uk
Critical Care Unit, University Hospitals Birmingham NHS Foundation Trust, Birmingham, UK

Kurtis Poole, Research Associate- kurtis.poole@nhs.net
Warwick Medical School, University of Warwick, Coventry, UK

William Bradlow, Consultant- William.bradlow@uhb.nhs.uk
Department of Cardiology, University Hospitals Birmingham NHS Foundation Trust, Birmingham, UK

Aileen Clarke, Professor- aileen.clarke@warwick.ac.uk
Warwick Medical School, University of Warwick, Coventry, UK

Gavin D Perkins, Professor,- g.d.perkins@warwick.ac.uk
Warwick Medical School, University of Warwick, Coventry, UK
Critical Care Unit, University Hospitals Birmingham NHS Foundation Trust, Birmingham, UK

Pamela Royle, Research Fellow- p.l.royle@warwick.ac.uk
Warwick Medical School, University of Warwick, Coventry, UK

Joyce Yeung, Associate Professor- j.yeung.4@warwick.ac.uk
Warwick Medical School, University of Warwick, Coventry, UK
Critical Care Unit, University Hospitals Birmingham NHS Foundation Trust, Birmingham, UK

Sian Taylor-Phillips, Associate Professor- s.taylor-phillips@warwick.ac.uk
Warwick Medical School, University of Warwick, Coventry, UK

Corresponding author: Dr Keith Couper, Warwick Clinical Trials Unit, Warwick Medical School, University of Warwick, Gibbet Hill Road, Coventry, CV4 7AL
Tel: 02476 151179. Email: k.couper@warwick.ac.uk

For peer review only

Abstract

Objective: To summarise studies describing incidence of sudden cardiac death in a general population of young individuals to inform screening policy.

Design: Systematic review.

Data sources: Database searches of MEDLINE, EMBASE, and the Cochrane library (all inception to current) on 29th April 2019 (updated 16th November 2019), and forward/ backward citation tracking of eligible studies.

Study eligibility criteria: All studies that reported incidence of sudden of cardiac death in young individuals (12-39 years) in a general population, with no restriction on language or date. Planned sub-groups were incidence by age, sex, race, and athletic status (including military personnel).

Data extraction: Two reviewers independently assessed study eligibility, extracted study data, and assessed risk of bias using the Joanna Briggs Institute critical appraisal checklist for prevalence studies.

Analysis: Reported incidence of sudden cardiac death in the young per 100,000 person-years.

Results: 38 studies that reported incidence across five continents. We identified substantial heterogeneity in population, sudden cardiac death definition, and case ascertainment methods, precluding meta-analysis. Median reported follow-up years was 6.97 million (interquartile range 2.34 million to 23.70 million) and number of sudden cardiac death cases was 64 (interquartile range 40 to 251). In the general population, the median of reported incidence was 1.7 sudden cardiac death per 100,000 person-years (interquartile range (IQR) 1.3 to 2.6, range 0.75 to 11.9). Most studies (n=14, 54%) reported an incidence between one and two cases per 100,000 person-years. Incidence was higher in males and older individuals.

Conclusions: This systematic review identified variability in the reported incidence of sudden cardiac death in the young across studies. Most studies reported an incidence between one and two cases per 100,000 person-years.

PROSPERO registration PROSPERO (CRD42019120563).

Keywords: sudden cardiac death; epidemiology; incidence; systematic review

Word count: 2481

For peer review only

Strengths and limitations of this study

- A systematic review that identified 38 relevant international studies with no restriction on publication language.
- We were unable to pool study data due to substantial study heterogeneity.
- The definition of sudden cardiac death is challenging to apply particularly in large datasets, leading to a risk of misclassification bias in some studies.

For peer review only

For peer review only - http://bmjopen.bmj.com/site/about/guidelines.xhtml

Introduction

Cardiovascular disease is the leading cause of death worldwide.[1] In the young, however, deaths due to cardiovascular disease are much less frequent than deaths due to other causes such as unintentional injury, suicide, and homicide.[2] Nevertheless, over 20,000 young individuals aged under 45 die due to cardiovascular disease in the United States each year.[3] The sub-group of young cardiovascular deaths that occur suddenly has a particularly profound effect on the individual's family and local community.

Screening of asymptomatic individuals for cardiac conditions, such as cardiomyopathies and channelopathies, has been proposed as a strategy to reduce the incidence of sudden cardiac death in young people. The best evidence that such a strategy might be effective comes from a single Italian before-after study that reported a decreased incidence of sudden cardiac death in athletes following the introduction of a mandatory athlete screening programme.[4] In view of the limitations of the current evidence, screening of young asymptomatic non-athletes is not presently supported by either the American Heart Association or European Society of Cardiology.[5-7]

For organisations that make decisions regarding the implementation of population screening programmes, such as the US Preventive Services Task Force and UK National Screening Committee, a clear understanding of the incidence of the target condition provides important context for decision-making.[8 9] In particular, incidence is an indicator of the size of the health problem, and maximum benefit which could be gained from screening, to be balanced against the potential harms such as stopping young people from participating in exercise. Previous reviews of sudden cardiac death incidence have focussed on the incidence across all ranges, or in specific populations.[10 11] The aim of this systematic review is to describe current evidence on the incidence of sudden cardiac death in the young.

Methods

We conducted a systematic review of studies that report the incidence of sudden cardiac death in the young. We were originally commissioned by the UK National Screening Committee to undertake a rapid review of incidence studies. We subsequently chose to develop the rapid review in to this systematic review, with no limitation on study location, date, or publication language.

The review is written in accordance with the Preferred Reporting Items for Systematic Reviews and Meta-Analyses (PRISMA) guidelines. The review is registered with PROSPERO (CRD42019120563).[12]

Search and study selection

We searched MEDLINE (1946-current), EMBASE (1947-current) and the Cochrane library (inception-current) for eligible studies. The search strategy was developed by an information scientist. We, used a combination of keywords and MESH terms to describe the population (e.g. young adult, adolescent), condition (e.g. sudden death, sudden cardiac death), and study type (e.g. cohort, longitudinal) of interest. An example search strategy is included in the electronic supplement.

Following searches and duplicate removal, two reviewers independently screened study titles and abstracts. Conflicts were resolved through discussion or, where needed, arbitration by a third reviewer. The same process was adopted for review of full-text papers. We identified additional studies through forward and backward citation tracking of included studies.

For peer review only - http://bmjopen.bmj.com/site/about/guidelines.xhtml

Study eligibility criteria

We included studies that described the incidence (per 100,000 person-years) of sudden cardiac death in the young, or that provided sufficient data to allow calculation of the incidence. We defined a young individual as someone aged 12-39 years. For reasons of pragmatism, we included studies where the reported incidence combined individuals in our target age range with younger individuals (≥ one-year). Exclusion of individuals under one-year avoided the risk of conflating sudden cardiac death with sudden infant death syndrome. We excluded studies where the reported incidence combined individuals in our target age range with older individuals (≥ 40-years), due to the marked increase in sudden cardiac death incidence from the age of 40.[13]

We also excluded studies that reported incidence only in a population with previously known disease, or only included deaths that occurred at certain times of day (e.g. at work or school) or during specific activities (e.g. sports). We imposed no restriction on language or publication date. We did not mandate a specific definition of sudden cardiac death or case-ascertainment process, but these were recorded during data extraction and considered in risk of bias assessments.

Our main population of interest was the general population aged 12-39. To be included in the main analysis, studies were required to report incidence in a general population that included males and females with an age group that spanned at least ten-years between 12 and 39 years. Where studies reported more than one age sub-group (e.g. 12-21 years; 22-31 years), we selected the sub-group closest to the age of mid-late teens to be included in the main analysis. Our planned sub-groups included incidence by age, sex, race, and athletic status (including military personnel). We do not report sub-groups of sub-groups (for example, athletes broken down by sex).

Data extraction and analysis

We used a piloted pro forma to extract population characteristics, study design case identification methods, use of systematic screening, and outcome data. We assessed risk of bias using the Joanna Briggs Institute critical appraisal checklist for prevalence studies.[14] We developed review-specific criteria for each checklist question. Two reviewers independently extracted data and assessed risk of bias, with discrepancies resolved through discussion.

We extracted incidence per 100,000 person-years and associated 95% confidence interval from the papers. Where these data were not reported, we calculated them from the reported number of sudden cardiac deaths and person follow-up years. We computed the 95% confidence interval based on a binomial distribution, using Stata Version 15.1. Clinical heterogeneity in relation to population characteristics and case ascertainment methods precluded pooling of data.

Patient/ public involvement

We did not involve patients or members of the public in setting the research question, designing the study, undertaking the study, the interpretation of the results, or study write-up.

Results

We performed initial database searches on 29th April 2019, and updated searches on 16th November 2019. Through searches and citation tracking, we identified 8360 unique citations of which we

For peer review only - http://bmjopen.bmj.com/site/about/guidelines.xhtml

reviewed the full-text of 238. We excluded 27 of the 65 papers initially included due to substantial or complete data overlap with other studies (figure one).

Most of the 38 included studies[4 15-51] were undertaken in Europe (n=20, 53%) and North America (n=14, 37%) (table one). The majority were retrospective studies (n=29, 76%). Median number of deaths and person follow-up years were 64 (interquartile range 40 to 251) and 6.97 million (interquartile range 2.34 million to 23.70 million) respectively. Study duration ranged from one-year to 41-years.

There was variability in the population, case ascertainment methods, and sudden cardiac death definition used between studies. This methodological heterogeneity precluded meta-analysis. Only nine studies (24%) stated that they used the AHA/ESC definition of sudden cardiac death. Five (13%) studies identified sudden cardiac death cases using death certificates, with the remaining using a range of approaches that incorporated autopsy data to some extent. Our risk of bias assessment found that most studies were at high risk of bias due to the method used to identify sudden cardiac death (figure two; electronic supplement table S1).

Our main analysis included 26 studies (figure three). The median of reported incidence was 1.7 sudden cardiac death per 100,000 person-years (interquartile range (IQR) 1.3 to 2.6, range 0.75 to 11.9). Of the 26 studies, 17 (65%) reported an incidence of less than two sudden cardiac death events per 100,000 person-years, and 14 (54%) studies reported an incidence between one and two cases per 100,000 person-years. All four studies reporting an incidence over three cases per 100,000 person-years were at high risk of bias across multiple domains.

Geographically, incidence appeared highest in African and Asian countries, although this may in part reflect the case ascertainment process and associated risk of bias. These studies also tended to include only older individuals (≥ 18-years).

For our sub-group analysis, sex, age, athletic status, and race were reported in 13, 11, 9, and 1 studies respectively. We observed a consistently higher incidence in males (figure four). In males, median incidence was 4.4 sudden cardiac death per 100,000 person-years (IQR 1.8 to 4.4, range 1.3 to 36), compared with a median incidence in females of 0.9 cases per 100,000 person-years (IQR 0.6 to 1.4, range 0 to 7.0).

The use of different age cut-offs within studies made comparison between studies on the association of incidence of sudden cardiac death with age challenging (electronic supplement figure S1).

Incidence in military personnel was reported in three studies and appeared to be consistently higher than in other populations (electronic supplement figure S2). All three studies were undertaken in American military personnel and reported that individuals received pre-enrolment screening for cardiac conditions. We did not observe a clear difference in incidence between athletic and non-athletic populations. The highest incidence was reported in a prospective study of screened UK soccer players, although the low number of sudden cardiac deaths (8 events) means that the estimate of the incidence is imprecise.

Race was reported in a single American study which broke incidence down in to four categories (African American, White, Hispanic, other). Incidence was highest in the African-American population (electronic supplement figure S3).

For peer review only - http://bmjopen.bmj.com/site/about/guidelines.xhtml

Discussion

In this systematic review, we included 38 studies from five continents that reported incidence of sudden cardiac death in young individuals. We identified significant variability in the reported incidence across studies. In our main analysis, most studies reported an incidence of between one and two sudden cardiac deaths per 100,000 person-years, although reported incidence ranged from 0.75 to 11.9 cases per 100,000 person-years. This variability may reflect differences in population, case-ascertainment methods, and definition used for sudden cardiac death. In pre-defined subgroups, we observed that male sex and increasing age seem to be associated with increased incidence.

Strengths and weaknesses of the study

The key strength of this review was the systematic identification and synthesis of 38 studies from a wide range of settings. We did not restrict inclusion by date and language, with fluent speakers used for translation.

Our review has four key limitations. Firstly, whilst the geographical area covered by index studies was often large with a long recruitment period, the median number of sudden cardiac deaths identified per study was 64 cases. As such, misclassification bias may have significantly impacted the reported incidence in some studies.[52] Secondly, heterogeneity between studies in terms of the population and case ascertainment method used precluded meta-analysis. Thirdly, particularly for sub-groups, studies often described only the absolute incidence, and did not provide sufficient additional data to calculate the 95% confidence interval. As such, we could not describe the precision of the incidence rate in many studies. Finally, we limited our condition of interest to sudden cardiac death, and did not examine incidence of sudden cardiac arrest. Whilst survival after sudden cardiac arrest is associated with important morbidity, the standard Utstein categorisation of cardiac arrest cause encompasses all medical causes, of which cardiac causes are only a sub-set.[53 54]

Comparison with other studies

Our review builds on two previous systematic reviews that described sudden cardiac death incidence across all age groups or in specific groups of young individuals.[10 11] In line with our review findings, these reviews reported issues across index studies in relation to variability in reported incidence, use of different definitions of sudden cardiac death, and use of different methods to identify cases of sudden cardiac death.

Variability in reported incidence reflects in part the challenge of accurately identifying cases of sudden cardiac death. The classdefinition of sudden cardiac death is based on both the circumstances and cause of death, such that classification requires knowledge of the cause of death and events leading up to the death.[5 6] Death certificate data provide an efficient way to estimate sudden cardiac death incidence, but lack of information on the circumstances of the death means studies relying on these data likely over-estimate incidence.[18]

Determination of the cause of sudden cardiac death usually requires an autopsy, ideally by a specialist pathologist.[55 56] In cases where cause of death remains uncertain after autopsy, genetic testing of the deceased may be informative in determining both the cause of death and the need for cardiac screening of the victim's family.[16 56 57] In an Australasian study, genetic testing of 113 cases of unexplained sudden cardiac death cases identified cardiac gene variants that were definitely or probably pathogenic in over a quarter of cases.[16]

Studies included in this review spanned a 66-year period (1950 to 2016), although most studies report data collected since 2000. Recent observational studies provide evidence of a decreasing

For peer review only - http://bmjopen.bmj.com/site/about/guidelines.xhtml

incidence of sudden cardiac death.[24 41 58] A Danish study reported a 3% average annual decrease in sudden cardiac death incidence between 2000 and 2009.[58] Potential explanations for this decrease include improved investigation of inherited cardiac disease, public health strategies to deal with obesity and smoking, and improved response to out-of-hospital cardiac arrest..

Clinical and policy implications

The decision to implement screening programmes in both the UK and USA requires evidence that the benefits of screening outweigh its harms.[9 59] Disease incidence data provides important context for evaluating evidence, particularly in the absence of direct evidence of benefit from randomised controlled trials. In less common conditions, a key concern are the potential harms that may result from false-positive results, and overdiagnosis of disease which may never become symptomatic. Nevertheless, rare disease may still meet criteria for population-based screening conditions, such as maple syrup urine disease in newborns.[60]

The incidence of a condition describes the number of individuals that might benefit from screening. In practice, not all young individuals that sustain sudden cardiac death have the potential to benefit from screening. Firstly, to be effective, screening must reliably identify individuals with disease. In this context, screening must detect a range of structural and electrical cardiac disease. In a prospective cohort study of 11,168 screened teenage footballers, six out of eight individuals who subsequently experienced sudden cardiac death did not disease detected at screening.[22]

Secondly, population-level screening is targeted at asymptomatic individuals without family history of sudden cardiac death. However, index studies included groups at increased risk of sudden cardiac death, such as individuals with diagnosed cardiac disease, asymptomatic individuals with a family history of sudden cardiac death, and symptomatic individuals without diagnosed cardiac disease.[61-63] In the latter two groups, testing for cardiac disease is supported by current guidelines.[6 64-66]

Finally, effective screening relies on the individuals willingness to be screened. To date, most studies report screening which, whilst technically voluntary, was mandated for participation in an activity, such as sport. Studies of athletes report young people are supportive of screening.[67 68] In practice, however, uptake of school-based cardiac screening programmes varies from 56% to 79%, with substantial variation by year and school.[69 70]

In conclusion, studies of incidence of sudden cardiac death in young individuals have produced varying estimates of the incidence, although most studies report the incidence to be between one and two cases per 100,000 person-years. In these studies of young individuals, incidence was highest in males and amongst older individuals.

For peer review only

For peer review only - http://bmjopen.bmj.com/site/about/guidelines.xhtml

Table and figure list

Figure one: PRISMA flow chart of study selections

Figure two: Risk of bias assessment

Figure three: Incidence of sudden cardiac death in general population

Figure footnote: Range following study name indicates population age range

Figure four: Incidence of sudden cardiac death in general population- sex sub-groups

Table one: summary of included studies

Author contributions

KC accepts full responsibility for the work and conduct of the study, had access to the data, and controlled the decision to publish.

KC designed the study, acted as reviewer, undertook the analysis, and drafted the manuscript. OP and RF acted as reviewers. PR designed database searches. KP, WB, AC, GDP, JY and STP contributed to study design. All authors contributed to the write up and approved the final version.

Ethics approval

Not required

Data sharing statement

All data relevant to the study are included in the article or uploaded as supplementary information

Funding

This study was based on a rapid review originally commissioned and funded by the UK National Screening Committee. This systematic review used the same research question as commissioned for the rapid review. Otherwise, the UK National Screening Committee had no role in study design; collection, analysis, and interpretation of data; in the writing of the report; and in the decision to submit the article for publication. All authors, external and internal, had full access to all of the data (including statistical reports and tables) in the study and can take responsibility for the integrity of the data and the accuracy of the data analysis.

This research is supported by the National Institute for Health Research (NIHR) Applied Research Collaboration (ARC) West Midlands. Professor Perkins is supported as an NIHR senior investigator. Dr Taylor-Phillips is supported by an NIHR Career Development Fellowship (CDF-2016-09-018)”.

The opinions are those of the authors and not the NIHR, the UK National Screening Committee, the NHS or the Department of Health and Social Care.

For peer review only

Competing interests

All authors have completed the ICMJE uniform disclosure at www.icmje.org/coi_disclosure.pdf and declare: KC, KP, AC, PR, GDP, JY, STP received financial support from Public Health England for the rapid review on which this review was based; no financial relationships with any organisations that might have an interest in the submitted work in the previous three years; KC, JY and GDP have volunteer roles with the International Liaison Committee on Resuscitation, European Resuscitation Council and Resuscitation Council UK.

For peer review only

For peer review only - http://bmjopen.bmj.com/site/about/guidelines.xhtml

References

1. World Health Organisation. World Health Statistics 2018: Monitoing health for the SDGs (sustainable development goals). Geneva, 2018. [Available from http://apps.who.int/iris/bitstream/handle/10665/272596/9789241565585-eng.pdf?ua=1 accessed 13/02/2020]

2. Center for Disease Control and Prevention. 10 leading causes of death by age group, United States- 2017 [Available from: https://www.cdc.gov/injury/wisqars/LeadingCauses.html accessed 20/12/2019]

3. Benjamin EJ, Muntner P, Alonso A, et al. Heart Disease and Stroke Statistics- 2019 Update: A Report From the American Heart Association. Circulation 2019;139(10):e56-e528.

4. Corrado D, Basso C, Pavei A, et al. Trends in Sudden Cardiovascular Death in Young Competitive Athletes After Implementation of a Preparticipation Screening Program. JAMA 2006;296(13):1593-601.

5. Al-Khatib SM, Stevenson WG, Ackerman MJ, et al. 2017 AHA/ACC/HRS Guideline for Management of Patients With Ventricular Arrhythmias and the Prevention of Sudden Cardiac Death. Circulation 2018;138(13):e272-e391.

6. Priori SG, Blomström-Lundqvist C, Mazzanti A, et al. 2015 ESC Guidelines for the management of patients with ventricular arrhythmias and the prevention of sudden cardiac death. Eur Heart J 2015;36(41):2793-867.

7. Van Brabandt H, Desomer A, Gerkens S, et al. Harms and benefits of screening young people to prevent sudden cardiac death. BMJ 2016;353:i1156.

8. US Preventive Services Task Force. Procedure Manual 2018 [Available from: https://www.uspreventiveservicestaskforce.org/Page/Name/procedure-manual accessed 08/01/2020]

9. Public Health England. Criteria for appraising the viability, effectiveness and appropriateness of a screening programme. Available from: https://www.gov.uk/government/publications/evidence-review-criteria-national-screening-programmes/criteria-for-appraising-the-viability-effectiveness-and-appropriateness-of-a-screening-programme accessed 13/02/2020]

10. Harmon KG, Drezner JA, Wilson MG, et al. Incidence of sudden cardiac death in athletes: a state-of-the-art review. Heart 2014;100(16):1227-34.

11. Kong MH, Fonarow GC, Peterson ED, et al. Systematic Review of the Incidence of Sudden Cardiac Death in the United States. JACC 2011;57(7):794-801.

12. Moher D, Liberati A, Tetzlaff J, et al. Preferred reporting items for systematic reviews and meta-analyses: the PRISMA statement. Ann Intern Med 2009;151(4):264-9.

13. Risgaard B, Winkel BG, Jabbari R, et al. Burden of sudden cardiac death in persons aged 1 to 49 years: nationwide study in Denmark. Circ Arrhythm Electrophysiol 2014;7(2):205-11.

For peer review only

14. Joanna Briggs Institute. Joanna Briggs Institute Checklist for Prevalence Studies 2017 [Available from: http://joannabriggs.org/assets/docs/critical-appraisal-tools/JBI_Critical_Appraisal-Checklist_for_Prevalence_Studies2017.pdf accessed 17/03/2019].

15. Anastasakis A, Papatheodorou E, Ritsatos K, et al. Sudden unexplained death in the young: epidemiology, aetiology and value of the clinically guided genetic screening. EP Europace 2018;20(3):472-80.

16. Bagnall RD, Weintraub RG, Ingles J, et al. A Prospective Study of Sudden Cardiac Death among Children and Young Adults. NEJM 2016;374(25):2441-52.

17. Bonny A, Tibazarwa K, Mbouh S, et al. Epidemiology of sudden cardiac death in Cameroon: The first population-based cohort survey in sub-Saharan Africa. Int J Epidemiol 2017;46(4):1230-38.

18. Chugh SS, Jui J, Gunson K, et al. Current burden of sudden cardiac death: Multiple source surveillance versus retrospective death certificate-based review in a large U.S. community. JACC 2004;44(6):1268-75.

19. Eckart RE, Shry EA, Burke AP, et al. Sudden death in young adults: An autopsy-based series of a population undergoing active surveillance. JACC 2011;58(12):1254-61.

20. Fragkouli K, Vougiouklakis T. Sudden cardiac death: An 11-year postmortem analysis in the region of Epirus, Greece. Pathol Res Pract 2010;206(10):690-94.

21. Hofer F, Fellmann F, Schlapfer J, et al. Sudden cardiac death in the young (5-39 years) in the canton of Vaud, Switzerland. BMC Cardiovasc Disord 2014;14:140.

22. Malhotra A, Dhutia H, Finocchiaro G, et al. Outcomes of Cardiac Screening in Adolescent Soccer Players. NEJM 2018;379(6):524-34.

23. Papadakis M, Sharma S, Cox S, et al. The magnitude of sudden cardiac death in the young: a death certificate-based review in England and Wales. EP Europace 2009;11(10):1353-8.

24. Wisten A, Krantz P, Stattin EL. Sudden cardiac death among the young in Sweden from 2000 to 2010: an autopsy-based study. EP Europace 2017;19(8):1327-34.

25. Winkel BG, Risgaard B, Bjune T, et al. Gender differences in sudden cardiac death in the young-a nationwide study. BMC Cardiovasc Disord 2017;17:19.

26. Pilmer CM, Kirsh JA, Hildebrandt D, et al. Sudden cardiac death in children and adolescents between 1 and 19 years of age. Heart Rhythm 2014;11(2):239-45.

27. Pilmer CM, Porter B, Kirsh JA, et al. Scope and nature of sudden cardiac death before age 40 in Ontario: a report from the cardiac death advisory committee of the office of the chief coroner. Heart Rhythm 2013;10(4):517-23.

28. Harmon KG, Asif IM, Maleszewski JJ, et al. Incidence, Cause, and Comparative Frequency of Sudden Cardiac Death in National Collegiate Athletic Association Athletes: A Decade in Review. Circulation 2015;132(1):10-9.

29. Harmon KG, Asif IM, Maleszewski JJ, et al. Incidence and Etiology of Sudden Cardiac Arrest and Death in High School Athletes in the United States. Mayo Clin Proc 2016;91(11):1493-502.

30. Maron BJ, Haas TS, Murphy CJ, et al. Incidence and causes of sudden death in U.S. college athletes. JACC 2014;63(16):1636-43.

For peer review only - http://bmjopen.bmj.com/site/about/guidelines.xhtml

31. Maron BJ, Haas TS, Duncanson ER, et al. Comparison of the Frequency of Sudden Cardiovascular Deaths in Young Competitive Athletes Versus Nonathletes: Should We Really Screen Only Athletes? Am J Cardiol 2016;117(8):1339-41.

32. Morentin B, Audicana C. Population-based study of out-of-hospital sudden cardiovascular death: incidence and causes of death in middle-aged adults. Rev Esp Cardiol 2011;64(1):28-34.

33. Zhang J, Zhou X, Xing Q, et al. Epidemiological investigation of sudden cardiac death in multiethnic Xinjiang Uyghur autonomous region in Northwest China. BMC Public Health 2019;19(1):116.

34. Wren C, O'Sullivan JJ, Wright C. Sudden death in children and adolescents. Heart 2000;83(4):410-3.

35. Wisten A, Forsberg H, Krantz P, et al. Sudden cardiac death in 15-35-year olds in Sweden during 1992-99. J Intern Med 2002;252(6):529-36.

36. Vaartjes I, Hendrix A, Hertogh EM, et al. Sudden death in persons younger than 40 years of age: incidence and causes. Eur J Cardiovasc Prev Rehabil 2009;16(5):592-6.

37. Uuskula M, Lamp K, Vali M. An age-related difference in the ratio of sudden coronary death over acute myocardial infarction in Estonian males. J Clin Epidemiol 1998;51(7):577-80.

38. Morentin B, Paz Suarez-Mier M, Audicana C, et al. Incidence and causes of sudden death in persons less than 36 years of age. Med Clin 2001;116(8):281-5.

39. Margey R, Roy A, Tobin S, et al. Sudden cardiac death in 14- to 35-year olds in Ireland from 2005 to 2007: a retrospective registry. EP Europace 2011;13(10):1411-8.

40. Goudevenos JA, Papadimitriou ED, Papathanasiou A, et al. Incidence and other epidemiological characteristics of sudden cardiac death in northwest Greece. Int J Cardiol 1995;49(1):67-75.

41. El-Assaad I, Al-Kindi SG, Aziz PF. Trends of Out-of-Hospital Sudden Cardiac Death Among Children and Young Adults. Pediatrics 2017;140(6):e20171438.

42. Eckart RE, Scoville SL, Campbell CL, et al. Sudden death in young adults: a 25-year review of autopsies in military recruits. Ann Intern Med 2004;141(11):829-34.

43. Driscoll DJ, Edwards WD. Sudden unexpected death in children and adolescents. JACC 1985;5(6):118B-21B.

44. Asatryan B, Vital C, Kellerhals C, et al. Sports-related sudden cardiac deaths in the young population of Switzerland. PLoS ONE 2017;12(3):e0174434.

45. Hua W, Zhang L-F, Wu Y-F, et al. Incidence of Sudden Cardiac Death in China: Analysis of 4 Regional Populations. JACC 2009;54(12):1110-18.

46. Einarsson G, Björnsson J, Gunnarsson G. Sudden cardiac death in the young. A 30 year nation-wide study in Iceland. Int J Cardiol 2007;119:S34-S35.

47. Neuspiel DR, Kuller LH. Sudden and Unexpected Natural Death in Childhood and Adolescence. JAMA 1985;254(10):1321-25.

48. Drehner D, Neuhauser KM, Neuhauser TS, et al. Death among U.S. Air Force basic trainees, 1956 to 1996. Mil Med 1999;164(12):841-7.

For peer review only - http://bmjopen.bmj.com/site/about/guidelines.xhtml

49. Karvouni E, Anastasakis A, Spiliopoulou C, et al. Sudden death in the young in Greece. Hell J Cardiol 2000;41:350-70.

50. Anderson RE, Hill RB, Broudy DW, et al. A population-based autopsy study of sudden, unexpected deaths from natural causes among persons 5 to 39 years old during a 12-year period. Hum Pathol 1994;25(12):1332-40.

51. Perez G, Marrugat J, Sunyer J, et al. Sudden cardiac death in the counties of Girona. Med Clin 1992;99(13):489-92.

52. Delgado-Rodríguez M, Llorca J. Bias. Journal Epidemiol Community Health 2004;58(8):635-41.

53. Lilja G, Nielsen N, Bro-Jeppesen J, et al. Return to Work and Participation in Society After Out-of-Hospital Cardiac Arrest. Circ Cardiovasc Qual Outcomes 2018;11(1):e003566.

54. Perkins GD, Jacobs IG, Nadkarni VM, et al. Cardiac arrest and cardiopulmonary resuscitation outcome reports: update of the Utstein Resuscitation Registry Templates for Out-of-Hospital Cardiac Arrest: a statement for healthcare professionals from a task force of the International Liaison Committee on Resuscitation and the American Heart Association Emergency Cardiovascular Care Committee and the Council on Cardiopulmonary, Critical Care, Perioperative and Resuscitation. Circulation 2015;132(13):1286-300.

55. de Noronha SV, Behr ER, Papadakis M, et al. The importance of specialist cardiac histopathological examination in the investigation of young sudden cardiac deaths. EP Europace 2013;16(6):899-907.

56. Basso C, Aguilera B, Banner J, et al. Guidelines for autopsy investigation of sudden cardiac death: 2017 update from the Association for European Cardiovascular Pathology. Virchows Archiv 2017;471(6):691-705.

57. Lahrouchi N, Raju H, Lodder EM, et al. Utility of Post-Mortem Genetic Testing in Cases of Sudden Arrhythmic Death Syndrome. JACC 2017;69(17):2134-45.

58. Lynge TH, Nielsen JL, Blanche P, et al. Decline in incidence of sudden cardiac death in the young: a 10-year nationwide study of 8756 deaths in Denmark. EP Europace 2019;21(6):909-17.

59. U.S. Preventive Services Task Force. Grade Definitions 2012 [Available from: https://www.uspreventiveservicestaskforce.org/Page/Name/grade-definitions accessed 06/01/2019]

60. UK National Screening Committee. The UK NSC recommendation on Maple Syrup Urine Disease 2014 [Available from: https://legacyscreening.phe.org.uk/msud accessed 21st January 2020]

61. Norrish G, Field E, Mcleod K, et al. Clinical presentation and survival of childhood hypertrophic cardiomyopathy: a retrospective study in United Kingdom. Eur Heart J 2018;40(12):986-93.

62. Alexander PMA, Nugent AW, Daubeney PEF, et al. Long-Term Outcomes of Hypertrophic Cardiomyopathy Diagnosed During Childhood. Circulation 2018;138(1):29-36.

63. Ranthe MF, Winkel BG, Andersen EW, et al. Risk of cardiovascular disease in family members of young sudden cardiac death victims. Eur Heart J 2013;34(7):503-11.

64. Department of Health. National Service Framework for Coronary Heart Disease- Chapter Eight: Arrhythmias and Sudden Cardiac Death London: Department of Health, 2005. [Available from:

For peer review only

For peer review only - http://bmjopen.bmj.com/site/about/guidelines.xhtml

https://webarchive.nationalarchives.gov.uk/20100420225444/http://www.dh.gov.uk/en/Healthcare/Longtermconditions/Vascular/Coronaryheartdisease/DH_4117048 accessed 13/02/2020]

65. National Institute for Health and Care Excellence. Clinical Guideline 109: Transient loss of consciousness ('blackouts') in over 16s 2014 [Available from: https://www.nice.org.uk/guidance/cg109 accessed 18/03/2019]

66. Brignole M, Moya A, de Lange FJ, et al. 2018 ESC Guidelines for the diagnosis and management of syncope. Eur Heart J 2018;39(21):1883-948.

67. Asif IM, Annett S, Ewing JA, et al. Psychological impact of electrocardiogram screening in National Collegiate Athletic Association athletes. Br J Sports Med 2017;51(20):1489-92.

68. Asif IM, Johnson S, Schmieg J, et al. The psychological impact of cardiovascular screening: the athlete's perspective. Br J Sports Med 2014;48(15):1162-6.

69. Marek J, Bufalino V, Davis J, et al. Feasibility and findings of large-scale electrocardiographic screening in young adults: data from 32,561 subjects. Heart Rhythm 2011;8(10):1555-9.

70. Vilardell P, Brugada J, Aboal J, et al. Characterization of electrocardiographic findings in young students. Rev Esp Cardiol 2020;73(2):139-44.

For peer review only

Table one:

	Design	Setting	Population: Type/ Age	SCD cases	Follow-up years	Systematic screening	SCD definition	Case ascertainment	
								Identification of deaths	Main method to determine SCD
Anastasakis 2018	PC	Greece 2002-2010	General 1-35	226	12,750,000	No	AHA/ ESC	Government records/ death certificates	Autopsy record review
Anderson 1994	RC	USA 1977-1988	General 5-39	183	9,836,196	No	Other	Medical examiner records	Autopsy record review
Asatryan 2017	RC	Switzerland 1999-2010	General 10-39	349[†]	25,344,456	Athletes only	Other	Forensic medicine database	Autopsy record review
Bagnall 2016	PC	Austrasia 2010-2012	General 1-35	490	37,770,000	No	AHA/ ESC	Government records/ death certificates; Autopsy records	Autopsy record review
Bonny 2017	PC	Cameroon 2013	General 18-39	-	-	No	Other	Government records/ death certificates	Medical record review; interviews
Chugh 2004	PC	USA 2002-2003	General 10-34	-	-	No	AHA/ ESC	Hospital/ ambulance/ medical examiner records; death certificates	Autopsy/ medical record review; ICD code
Corrado 2006	PC	Italy 1979-2004	General 12-35	320	36,144,100	Athletes only	Other	Medical centre registers, media searches (prospective registry)	Autopsy record review
Drehner 1999	RC	USA 1956-1996	Military 17-26	23	340,990	Yes	Not stated	Military records	Autopsy record review
Driscoll 1985	RC	USA 1950-1982	General 1-22	7	930,678	No	Other	Government records/ death certificates	Autopsy/ medical record review
Eckart 2004	RC	USA 1977-2001	Milritary 17-35	64	-	Yes	Other	Military records	Autopsy/ military record review
Eckart 2011	RC	USA 1998-2008	Military <20	-	-	Yes	Other	Military records	Autopsy record review
Einarsson 2007	RC	Iceland 1974-2004	General 12-35	42	-	No	Not stated	Government records/ death certificates	Autopsy/ medical record review
El-Assaad 2017	RC	USA 1999-2015	General 1-34	31492	-	No	Other	Government records/ death certificates	ICD code
Fragkouli 2010	RC	Greece 1998-2008	General 1-35	28	-	No	Other	Forensic laboratory records	Autopsy/ medical/ police record review
Goudevenos 1995	PC	Greece 1990-1993	General 30-39	-	-	No	Other	Hospital/ coroner records; Department of statistics	Autopsy/ medical record review; interviews
Harmon 2015	RC	USA 2003-2013	Athletes 17-24	79	4,242,519	Yes	AHA/ ESC	Media search; NCAA records	Autopsy record review; interviews
Harmon 2016	RC	USA 2007-2013	Athletes 14-18	69	6,974,640	Yes	Other	Media search	Autopsy record review
Hofer 2014	RC	Switzerland 2000-2007	General 5-39	40	2,340,368	No	Not stated	Government records/ death certificates	ICD code
Hua 2009	PC	China 2005-2006	General 25-34	9	115,188	No	Other	Government records/ death certificates	Medical record review; interviews

For peer review only

Karvouni 2000	RC	Greece 1997-1999	General 1-35	33‡	4,033,333	No	Other	Forensic laboratory, hospital and ambulance records	Autopsy record review
Malhotra 2018	PC	UK 1996-2016	Athletes 15-17	8	118,351	Yes	Not stated	Voluntary reports/ survey; media search	Autopsy record review
Margey 2011	RC	Ireland 2005-2007	General 15-34	116	4,065,513	No	AHA/ ESC	Government records/ death certificates	Autopsy record review
Maron 2014	RC	USA 2002-2011	Athletes 17-26	64	4,052,369	No	Not stated	Media search; NCAA records	Autopsy record review
Maron 2016	RC	USA 2000-2014	General 14-23	27	1,308,730	No	Not stated	Medical examiner records	Autopsy record review
Morentin 2001	RC	Spain 1991-1998	General 1-35	46	4,413,088	No	Other	Forensic laboratory records	Autopsy record review
Morentin 2011	RC	Spain 2003-2008	General 35-39	42	-	No	AHA/ ESC	Government records/ death certificates	Autopsy record review
Neuspiel 1985	RC	USA 1972-1980	General 1-21	51	-	No	Other	Government records/ death certificates	Autopsy/ medical record review
Papadakis 2009	RC	UK 2002-2005	General 1-34	1677	94,256,200	No	Other	Government records/ death certificates	ICD code
Perez 1992	PC	Spain 1987-1988	General 25-34	1	-	No	Not stated	Government records/ death certificates	Medical record review; interviews
Pilmer 2013	RC	Canada 2008	General 2-29	56	-	No	Other	Coroner records	Autopsy/ medical/ police record review
Pilmer 2014	RC	Canada 2005-2009	General 1-19	116	14,893,860	No	Other	Coroner records	Autopsy record review
Uuskula 1998	RC	Estonia 1980-1996	General 20-39	251	902,241	No	Other	Government records/ death certificates	Autopsy record review
Vaartjes 2009	RC	Holland 1996-2006	General 1-39	1500	92,374,043	No	Other	Government records/ death certificates	ICD code
Winkel 2017	RC	Denmark 2000-2009	General 1-35	635	23,700,000	No	AHA/ ESC	Government records/ death certificates	Autopsy record review
Wisten 2002	RC	Sweden 1992-1999	General 15-35	181	19,514,080	No	AHA/ ESC	Government records/ national forensic medicine database	Autopsy/ medical record review
Wisten 2017	RC	Sweden 2000-2010	General 1-35	552	42,900,000	Athletes only	AHA/ ESC	Government records/ national forensic medicine database	Autopsy record review
Wren 2000	RC	England 1985-1994	General 1-20	59	8,060,000	No	Other	Coroner/ hospital records	Death certificate
Zhang 2019	RC	China 2015	General 18-35	-	-	No	Other	Government records/ death certificates	Medical record review; interviews

PC- Prospective cohort; RC- Retrospective cohort. AHA/ESC- American Heart Association/ European Society of Cardiology. NCAA National Collegiate Athletic Association. SCD- Sudden cardiac death.
† Study inflated number of SCDs to account for low autopsy rate. ‡- Unable to replicate reported incidence based on study data.

For peer review only - http://bmjopen.bmj.com/site/about/guidelines.xhtml

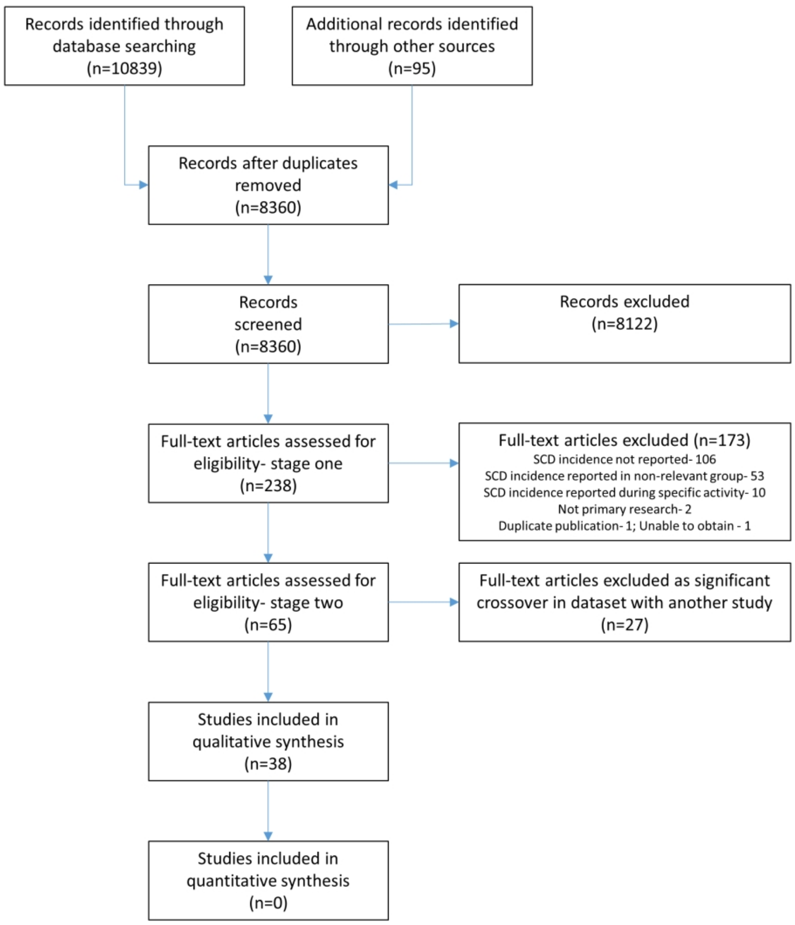

PRISMA flow chart of study selections

190x275mm (300 x 300 DPI)

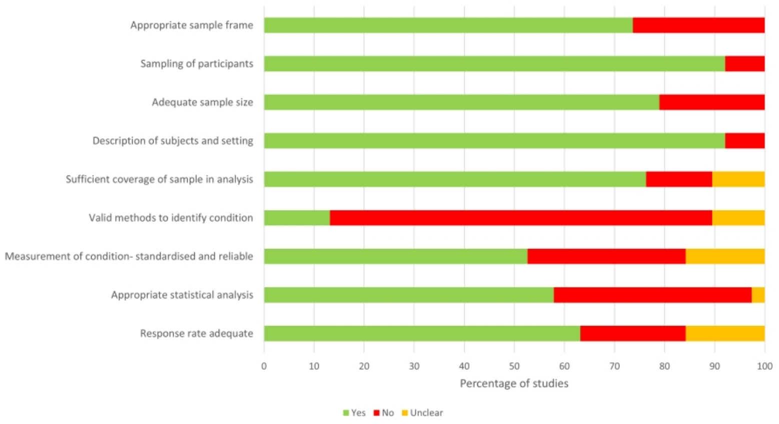

Risk of bias assessment

338x190mm (300 x 300 DPI)

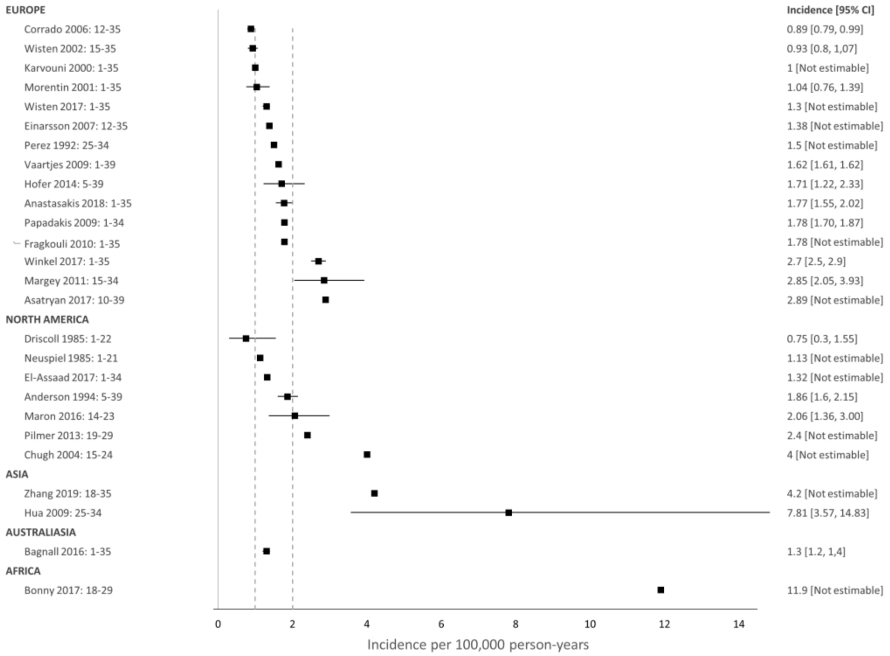

Incidence of sudden cardiac death in general population

Figure footnote: Range following study name indicates population age range

275x190mm (300 x 300 DPI)

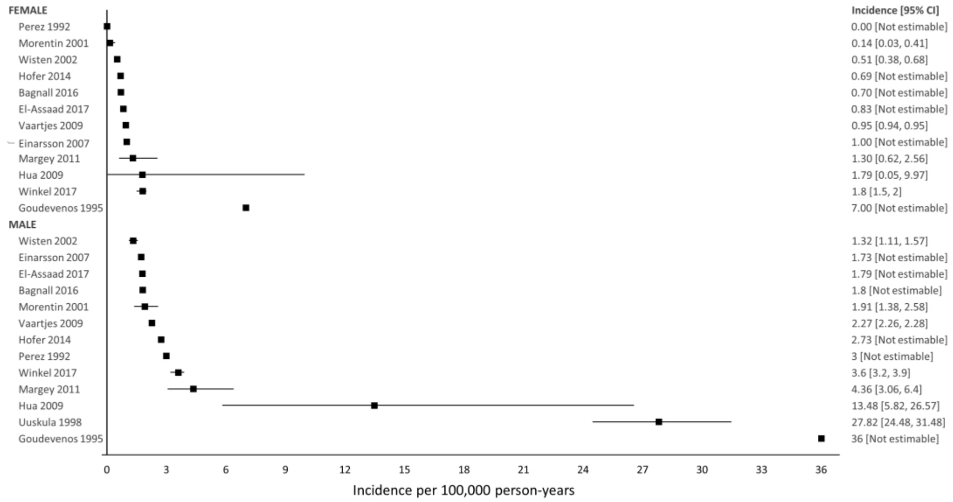

Incidence of sudden cardiac death in general population- sex sub-groups

275x190mm (300 x 300 DPI)

Incidence of sudden cardiac death in the young: a systematic review- Supplementary material

Contents:

MEDLINE search strategy- page 1

Table S1: study risk of bias assessment- page 2

Figure S1: Sudden cardiac death incidence by age- page 3

Figure S2: Sudden cardiac death incidence by athletic/ military status- page 4

Figure S3: Sudden cardiac death incidence by race- page 5

MEDLINE search strategy

1 exp Death, Sudden, Cardiac/
2 (sudden adj3 death).mp
3 cardiac death.mp
4 1 or 2 or 3
5 exp Incidence/
6 incidence.mp
7 exp Prevalence/
8 prevalence.mp
9 5 or 6 or 7 or 8
10 (adolescen* or young* or student* or athlet* or soccer or football*).mp
11 exp Young Adult/ or exp Adult/
12 exp Adolescent/
13 exp Child/
14 10 or 11 or 12 or 13
15 exp Cohort Studies/
16 exp Longitudinal Studies/
17 exp Follow-Up Studies/
18 exp Prospective Studies/
19 exp Retrospective Studies/
20 (longitudinal or prospective* or observational or registry).mp
21 (autopsy or death certificate*).mp
22 15 or 16 or 17 or 18 or 19 or 20 or 21
23 4 and 9 and 14 and 22
24 limit 23 to (case reports or comment or editorial or letter)
25 23 not 24

For peer review only - http://bmjopen.bmj.com/site/about/guidelines.xhtml

For peer review only

Table S1: study risk of bias assessment

	1-Appropriate sample frame	2-Sampling of participants	3-Adequate sample size	4-Description of subjects and setting	5-Sufficient coverage of sample in analysis	6-Valid methods to identify condition	7-Measurement of condition-standardised and reliable	8-Appropriate statistical analysis	9-Response rate adequate
Anastasakis 2018	Y	Y	Y	Y	Y	Y	Y	Y	Y
Anderson 1994	Y	Y	Y	Y	Y	N	Y	Y	Y
Asatryan 2017	Y	Y	Y	Y	Y	N	N	N	N
Bagnall 2016	Y	Y	Y	Y	Y	Y	Y	Y	Y
Bonny 2017	Y	N	N	N	U/C	N	Y	N	Y
Chugh 2004	Y	Y	N	Y	Y	N	N	N	Y
Corrado 2006	Y	Y	Y	Y	N	N	Y	Y	Y
Drehner 1999	N	Y	Y	Y	Y	N	U/C	N	N
Driscoll 1985	Y	Y	N	Y	Y	N	N	Y	Y
Eckart 2004	N	Y	Y	Y	Y	N	Y	Y	Y
Eckart 2011	N	Y	Y	Y	Y	N	Y	Y	N
Einarsson 2007	Y	Y	Y	Y	Y	U/C	U/C	U/C	U/C
El-Assaad 2017	Y	Y	Y	Y	Y	N	Y	N	Y
Fragkouli 2010	Y	Y	Y	Y	U/C	N	Y	N	U/C
Goudevenos 1995	N	Y	N	Y	Y	N	N	N	N
Harmon 2016	N	Y	Y	Y	N	N	N	Y	N
Harmon 2015	N	Y	Y	Y	N	N	N	Y	U/C
Hofer 2014	Y	Y	Y	Y	Y	N	N	Y	Y
Hua 2009	Y	N	N	N	N	N	N	Y	Y
Karvouni 2000	Y	Y	Y	Y	U/C	N	Y	N	U/C
Malhotra 2018	N	Y	N	Y	Y	U/C	Y	Y	Y
Margey 2011	Y	Y	Y	Y	Y	Y	Y	Y	N
Maron 2016	Y	Y	Y	Y	Y	U/C	U/C	Y	Y
Maron 2014	N	Y	Y	Y	N	U/C	Y	Y	N
Morentin 2001	Y	Y	Y	Y	Y	N	Y	Y	Y
Morentin 2011	N	Y	N	Y	Y	Y	U/C	N	Y
Neuspiel 1985	Y	Y	Y	Y	Y	N	N	N	Y
Papadakis 2009	Y	Y	Y	Y	Y	N	Y	Y	Y
Perez 1992	Y	Y	N	Y	Y	N	U/C	N	Y
Pilmer 2013	Y	Y	Y	Y	U/C	N	Y	N	U/C
Pilmer 2014	Y	Y	Y	Y	Y	N	Y	N	Y
Uuskula 1998	N	Y	Y	Y	Y	N	U/C	Y	U/C
Vaartjes 2009	Y	Y	Y	Y	Y	N	Y	Y	Y
Winkel 2017	Y	Y	Y	Y	Y	N	N	Y	Y
Wisten 2002	Y	Y	Y	Y	Y	Y	N	N	Y
Wisten 2017	Y	Y	Y	Y	Y	N	N	Y	N
Wren 2000	Y	Y	Y	Y	Y	N	Y	Y	Y
Zhang 2019	Y	N	Y	N	Y	N	Y	N	Y

Key: Y- Yes; N- No; U/C- Unclear

Full list of questions:

1. Was the sample frame appropriate to address the target population?
2.Were study participants sampled in an appropriate way?
3.Was the sample size adequate?
4.Were the study subjects and the setting described in detail?
5.Was the data analysis conducted with sufficient coverage of the identified sample?
6.Were valid methods used for the identification of the condition?
7.Was the condition measured in a standard, reliable way for all participants?
8.Was there appropriate statistical analysis?
9.Was the response rate adequate, and if not, was the low response rate managed appropriately?

Figure S1: Sudden cardiac death incidence by age

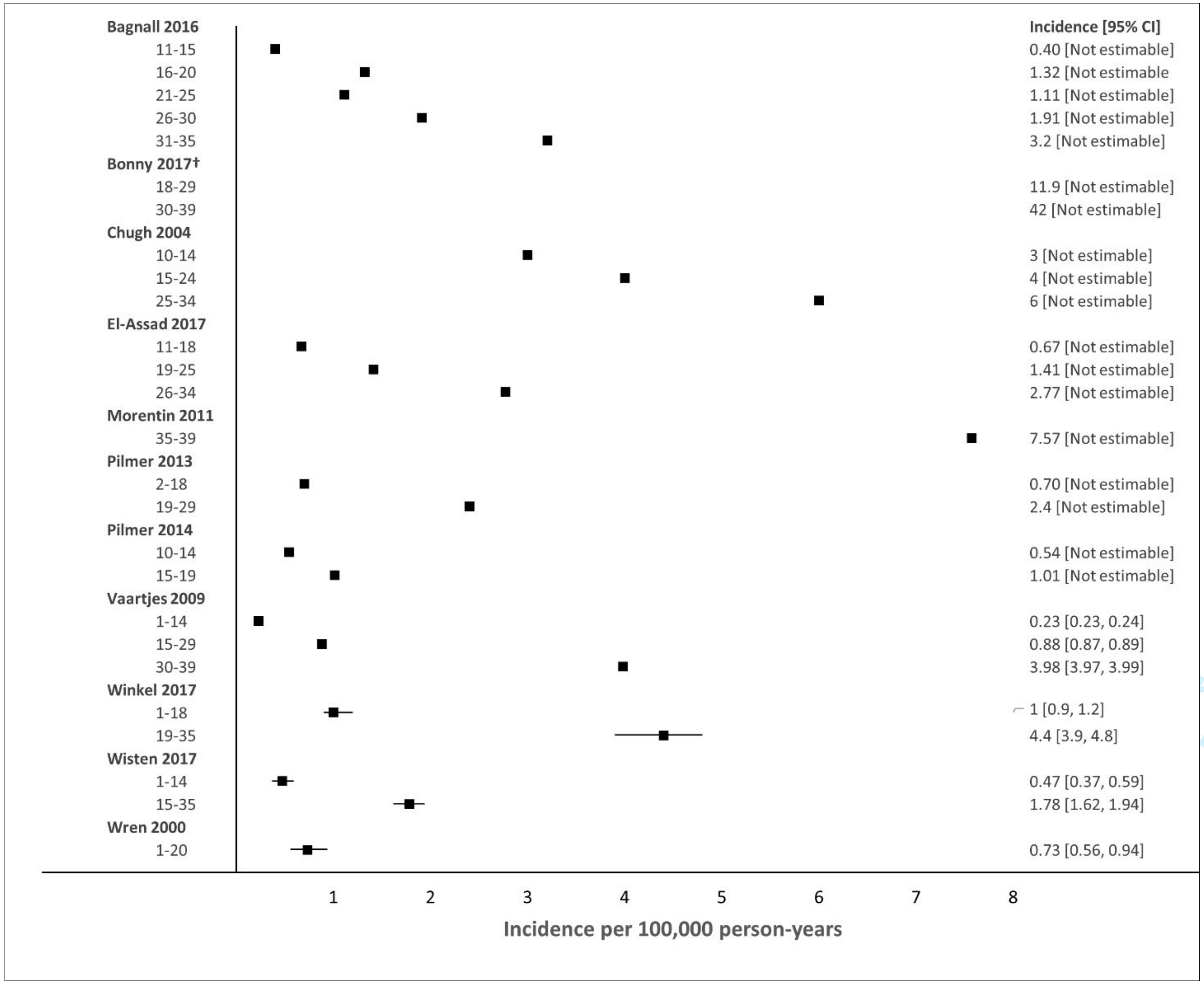

†- Data not presented on chart as significant outlier

For peer review only - http://bmjopen.bmj.com/site/about/guidelines.xhtml

Figure S2: Sudden cardiac death incidence by athletic/ military status

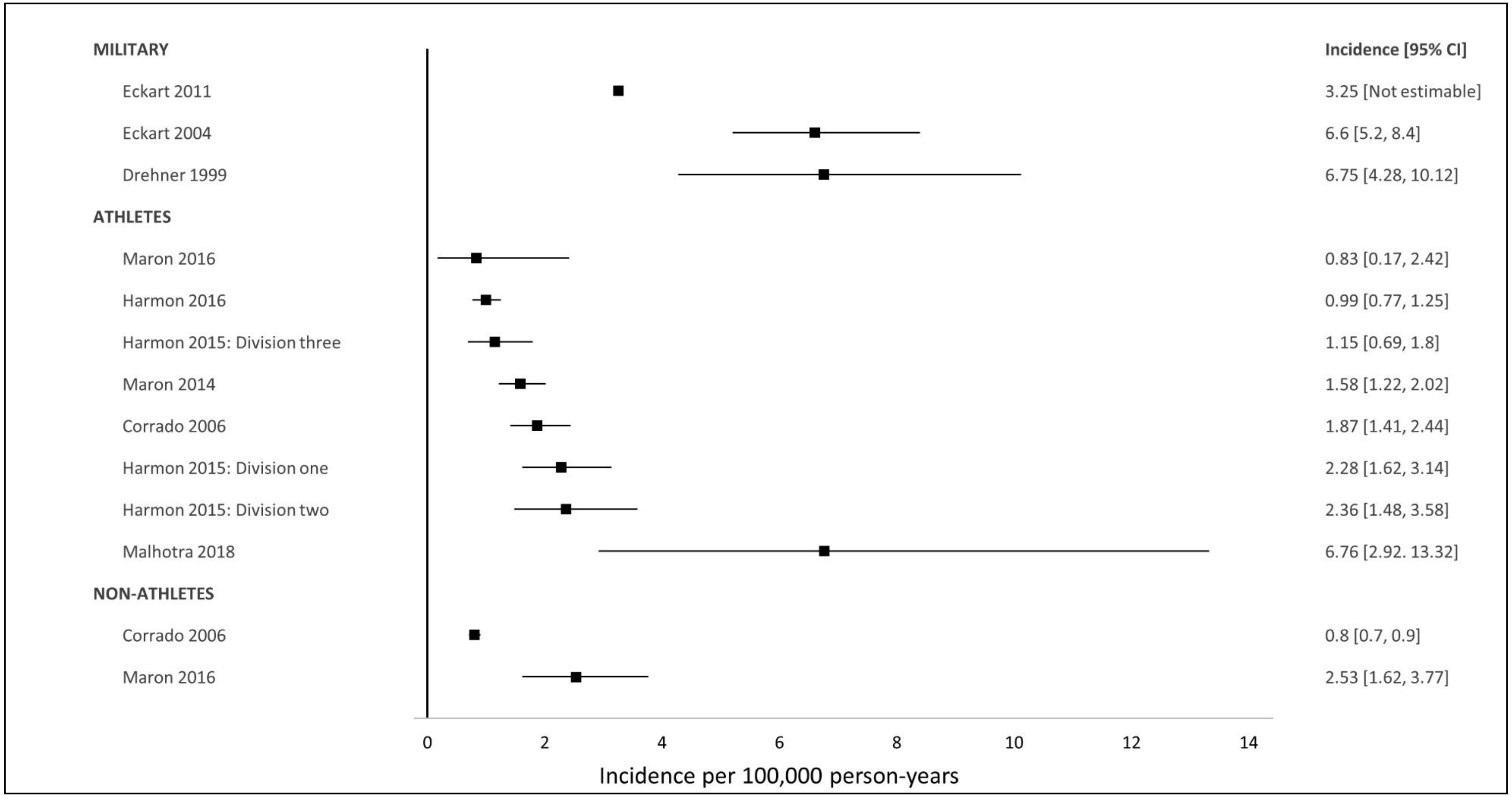

For peer review only - http://bmjopen.bmj.com/site/about/guidelines.xhtml

Figure S3: Sudden cardiac death incidence by race

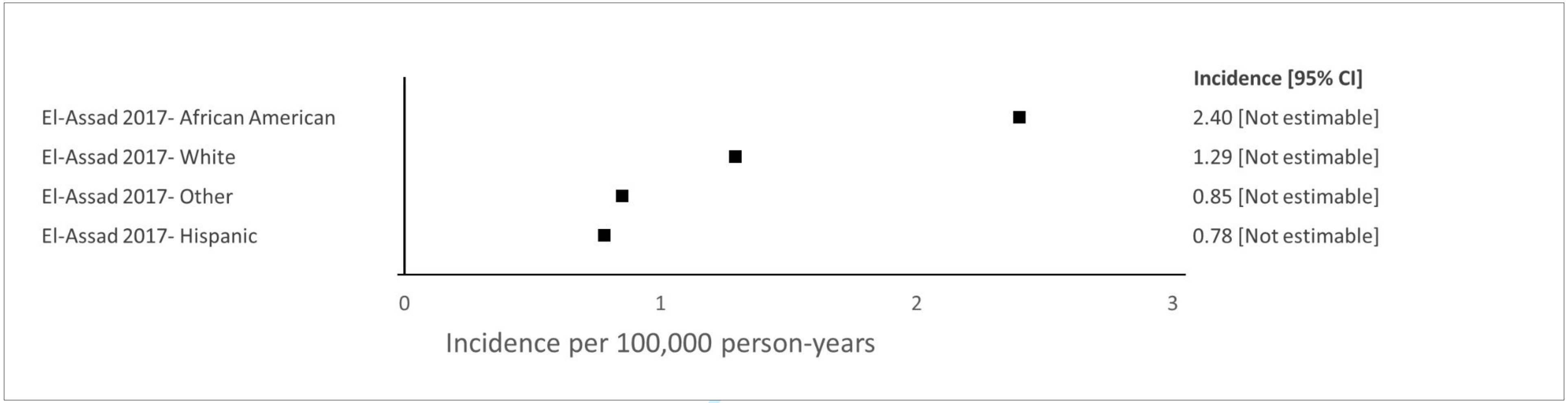

For peer review only - http://bmjopen.bmj.com/site/about/guidelines.xhtml

PRISMA 2009 Checklist

Section/topic	#	Checklist item	Reported on page #
**TITLE**			
Title	1	Identify the report as a systematic review, meta-analysis, or both.	1
**ABSTRACT**			
Structured summary	2	Provide a structured summary including, as applicable: background; objectives; data sources; study eligibility criteria, participants, and interventions; study appraisal and synthesis methods; results; limitations; conclusions and implications of key findings; systematic review registration number.	2
**INTRODUCTION**			
Rationale	3	Describe the rationale for the review in the context of what is already known.	4
Objectives	4	Provide an explicit statement of questions being addressed with reference to participants, interventions, comparisons, outcomes, and study design (PICOS).	4
**METHODS**			
Protocol and registration	5	Indicate if a review protocol exists, if and where it can be accessed (e.g., Web address), and, if available, provide registration information including registration number.	4
Eligibility criteria	6	Specify study characteristics (e.g., PICOS, length of follow-up) and report characteristics (e.g., years considered, language, publication status) used as criteria for eligibility, giving rationale.	5
Information sources	7	Describe all information sources (e.g., databases with dates of coverage, contact with study authors to identify additional studies) in the search and date last searched.	4
Search	8	Present full electronic search strategy for at least one database, including any limits used, such that it could be repeated.	Electronic supplement
Study selection	9	State the process for selecting studies (i.e., screening, eligibility, included in systematic review, and, if applicable, included in the meta-analysis).	4
Data collection process	10	Describe method of data extraction from reports (e.g., piloted forms, independently, in duplicate) and any processes for obtaining and confirming data from investigators.	5
Data items	11	List and define all variables for which data were sought (e.g., PICOS, funding sources) and any assumptions and simplifications made.	5
Risk of bias in individual studies	12	Describe methods used for assessing risk of bias of individual studies (including specification of whether this was done at the study or outcome level), and how this information is to be used in any data synthesis.	5
Summary measures	13	State the principal summary measures (e.g., risk ratio, difference in means).	5
Synthesis of results	14	Describe the methods of handling data and combining results of studies, if done, including measures of consistency (e.g., I^2) for each meta-analysis.	N/A

For peer review only - http://bmjopen.bmj.com/site/about/guidelines.xhtml

PRISMA 2009 Checklist

Section/topic	#	Checklist item	Reported on page #
Risk of bias across studies	15	Specify any assessment of risk of bias that may affect the cumulative evidence (e.g., publication bias, selective reporting within studies).	N/A
Additional analyses	16	Describe methods of additional analyses (e.g., sensitivity or subgroup analyses, meta-regression), if done, indicating which were pre-specified.	5
**RESULTS**			
Study selection	17	Give numbers of studies screened, assessed for eligibility, and included in the review, with reasons for exclusions at each stage, ideally with a flow diagram.	5-6/ figure one
Study characteristics	18	For each study, present characteristics for which data were extracted (e.g., study size, PICOS, follow-up period) and provide the citations.	Table one
Risk of bias within studies	19	Present data on risk of bias of each study and, if available, any outcome level assessment (see item 12).	Figure two/ electronic supplement
Results of individual studies	20	For all outcomes considered (benefits or harms), present, for each study: (a) simple summary data for each intervention group (b) effect estimates and confidence intervals, ideally with a forest plot.	Figure 3
Synthesis of results	21	Present results of each meta-analysis done, including confidence intervals and measures of consistency.	N/A
Risk of bias across studies	22	Present results of any assessment of risk of bias across studies (see Item 15).	N/A
Additional analysis	23	Give results of additional analyses, if done (e.g., sensitivity or subgroup analyses, meta-regression [see Item 16]).	Figure 4/ electronic supplemet
**DISCUSSION**			
Summary of evidence	24	Summarize the main findings including the strength of evidence for each main outcome; consider their relevance to key groups (e.g., healthcare providers, users, and policy makers).	7-8
Limitations	25	Discuss limitations at study and outcome level (e.g., risk of bias), and at review-level (e.g., incomplete retrieval of identified research, reporting bias).	7
Conclusions	26	Provide a general interpretation of the results in the context of other evidence, and implications for future research.	7-8
**FUNDING**			
Funding	27	Describe sources of funding for the systematic review and other support (e.g., supply of data); role of funders for the systematic review.	10

For peer review only - http://bmjopen.bmj.com/site/about/guidelines.xhtml

PRISMA 2009 Checklist

From: Moher D, Liberati A, Tetzlaff J, Altman DG, The PRISMA Group (2009). Preferred Reporting Items for Systematic Reviews and Meta-Analyses: The PRISMA Statement. PLoS Med 6(7): e1000097. doi:10.1371/journal.pmed1000097

For more information, visit: **www.prisma-statement.org**.

For peer review only

For peer review only - http://bmjopen.bmj.com/site/about/guidelines.xhtml

BMJ Open

Incidence of sudden cardiac death in the young: a systematic review

Journal:	*BMJ Open*
Manuscript ID	bmjopen-2020-040815.R1
Article Type:	Original research
Date Submitted by the Author:	18-Aug-2020

Complete List of Authors:	Couper, Keith; University of Warwick Warwick Medical School, Warwick Medical School; University Hospitals Birmingham NHS Foundation Trust, Critical Care Unit<br>Putt, Oliver; University of Warwick Warwick Medical School, Warwick Medical School<br>Field, Richard; University Hospitals Birmingham NHS Foundation Trust, Critical Care Unit<br>Poole, Kurtis; University of Warwick Warwick Medical School, Warwick Medical School<br>Bradlow, William; University Hospitals Birmingham NHS Foundation Trust, Department of cardiology<br>Clarke, Aileen; University of Warwick Warwick Medical School, Warwick Medical School<br>Perkins, Gavin; University of Warwick Warwick Medical School, Warwick Medical School; University Hospitals Birmingham NHS Foundation Trust, Critical Care Unit<br>Royle, Pamela; University of Warwick Warwick Medical School, Warwick Medical School<br>Yeung, Joyce ; University of Warwick Warwick Medical School, Warwick Medical School; University Hospitals Birmingham NHS Foundation Trust, Critical Care Unit<br>Taylor-Phillips, Sian; University of Warwick Warwick Medical School, Warwick Medical School

**Primary Subject Heading**:	Epidemiology
Secondary Subject Heading:	Epidemiology, Cardiovascular medicine
Keywords:	Cardiac Epidemiology < CARDIOLOGY, EPIDEMIOLOGY, Adult cardiology < CARDIOLOGY, Paediatric cardiology < PAEDIATRICS

SCHOLARONE™ Manuscripts

I, the Submitting Author has the right to grant and does grant on behalf of all authors of the Work (as defined in the below author licence), an exclusive licence and/or a non-exclusive licence for contributions from authors who are: i) UK Crown employees; ii) where BMJ has agreed a CC-BY licence shall apply, and/or iii) in accordance with the terms applicable for US Federal Government officers or employees acting as part of their official duties; on a worldwide, perpetual, irrevocable, royalty-free basis to BMJ Publishing Group Ltd ("BMJ") its licensees and where the relevant Journal is co-owned by BMJ to the co-owners of the Journal, to publish the Work in this journal and any other BMJ products and to exploit all rights, as set out in our licence.

The Submitting Author accepts and understands that any supply made under these terms is made by BMJ to the Submitting Author unless you are acting as an employee on behalf of your employer or a postgraduate student of an affiliated institution which is paying any applicable article publishing charge ("APC") for Open Access articles. Where the Submitting Author wishes to make the Work available on an Open Access basis (and intends to pay the relevant APC), the terms of reuse of such Open Access shall be governed by a Creative Commons licence – details of these licences and which Creative Commons licence will apply to this Work are set out in our licence referred to above.

Other than as permitted in any relevant BMJ Author's Self Archiving Policies, I confirm this Work has not been accepted for publication elsewhere, is not being considered for publication elsewhere and does not duplicate material already published. I confirm all authors consent to publication of this Work and authorise the granting of this licence.

For peer review only

Incidence of sudden cardiac death in the young: a systematic review

Keith Couper, Oliver Putt, Richard Field, Kurtis Poole, William Bradlow, Aileen Clarke, Gavin D Perkins, Pamela Royle, Joyce Yeung, Sian Taylor-Phillips

Keith Couper, Assistant Professor- k.couper@warwick.ac.uk
Warwick Medical School, University of Warwick, Coventry, UK
Critical Care Unit, University Hospitals Birmingham NHS Foundation Trust, Birmingham, UK

Oliver Putt, Academic Foundation Doctor- oliver.putt@nhs.net
Warwick Medical School, University of Warwick, Coventry, UK

Richard Field, Registrar- richiefield@doctors.org.uk
Critical Care Unit, University Hospitals Birmingham NHS Foundation Trust, Birmingham, UK

Kurtis Poole, Research Associate- kurtis.poole@nhs.net
Warwick Medical School, University of Warwick, Coventry, UK

William Bradlow, Consultant- William.bradlow@uhb.nhs.uk
Department of Cardiology, University Hospitals Birmingham NHS Foundation Trust, Birmingham, UK

Aileen Clarke, Professor- aileen.clarke@warwick.ac.uk
Warwick Medical School, University of Warwick, Coventry, UK

Gavin D Perkins, Professor,- g.d.perkins@warwick.ac.uk
Warwick Medical School, University of Warwick, Coventry, UK
Critical Care Unit, University Hospitals Birmingham NHS Foundation Trust, Birmingham, UK

Pamela Royle, Research Fellow- p.l.royle@warwick.ac.uk
Warwick Medical School, University of Warwick, Coventry, UK

Joyce Yeung, Associate Professor- j.yeung.4@warwick.ac.uk
Warwick Medical School, University of Warwick, Coventry, UK
Critical Care Unit, University Hospitals Birmingham NHS Foundation Trust, Birmingham, UK

Sian Taylor-Phillips, Professor- s.taylor-phillips@warwick.ac.uk
Warwick Medical School, University of Warwick, Coventry, UK

Corresponding author: Dr Keith Couper, Warwick Clinical Trials Unit, Warwick Medical School, University of Warwick, Gibbet Hill Road, Coventry, CV4 7AL
Tel: 02476 151179. Email: k.couper@warwick.ac.uk

For peer review only

Abstract

Objective: To summarise studies describing incidence of sudden cardiac death in a general population of young individuals to inform screening policy.

Design: Systematic review.

Data sources: Database searches of MEDLINE, EMBASE, and the Cochrane library (all inception to current) on 29th April 2019 (updated 16th November 2019), and forward/ backward citation tracking of eligible studies.

Study eligibility criteria: All studies that reported incidence of sudden of cardiac death in young individuals (12-39 years) in a general population, with no restriction on language or date. Planned sub-groups were incidence by age, sex, race, and athletic status (including military personnel).

Data extraction: Two reviewers independently assessed study eligibility, extracted study data, and assessed risk of bias using the Joanna Briggs Institute critical appraisal checklist for prevalence studies.

Analysis: Reported incidence of sudden cardiac death in the young per 100,000 person-years.

Results: 38 studies that reported incidence across five continents. We identified substantial heterogeneity in population, sudden cardiac death definition, and case ascertainment methods, precluding meta-analysis. Median reported follow-up years was 6.97 million (interquartile range 2.34 million to 23.70 million) and number of sudden cardiac death cases was 64 (interquartile range 40 to 251). In the general population, the median of reported incidence was 1.7 sudden cardiac death per 100,000 person-years (interquartile range (IQR) 1.3 to 2.6, range 0.75 to 11.9). Most studies (n=14, 54%) reported an incidence between one and two cases per 100,000 person-years. Incidence was higher in males and older individuals.

Conclusions: This systematic review identified variability in the reported incidence of sudden cardiac death in the young across studies. Most studies reported an incidence between one and two cases per 100,000 person-years.

PROSPERO registration PROSPERO (CRD42019120563).

Keywords: sudden cardiac death; epidemiology; incidence; systematic review

Word count: 2957

For peer review only

Strengths and limitations of this study

- This systematic review summarises studies that report the incidence of sudden cardiac death in the young.
- We searched key databases to identify relevant studies with no restriction on publication date or language.
- The reliable identification of sudden cardiac death is challenging, particularly in large retrospective studies, leading to a risk of misclassification bias.
- The definition of sudden cardiac death and methods used to identify sudden cardiac death varied across studies.
- Review findings will inform policy discussions on screening of asymptomatic young individuals for cardiac disease.

For peer review only

For peer review only - http://bmjopen.bmj.com/site/about/guidelines.xhtml

Introduction

Cardiovascular disease is the leading cause of death worldwide.[1] In the young, however, deaths due to cardiovascular disease are much less frequent than deaths due to other causes such as unintentional injury, suicide, and homicide.[2] Nevertheless, over 20,000 young individuals aged under 45 die due to cardiovascular disease in the United States each year.[3] The sub-group of young cardiovascular deaths that occur suddenly has a particularly profound effect on the individual's family and local community.

Screening of asymptomatic individuals for cardiac conditions, such as cardiomyopathies and channelopathies, has been proposed as a strategy to reduce the incidence of sudden cardiac death in young people. The best evidence that such a strategy might be effective comes from a single Italian before-after study that reported a decreased incidence of sudden cardiac death in athletes following the introduction of a mandatory athlete screening programme.[4] In view of the limitations of the current evidence, screening of young asymptomatic non-athletes is not presently supported by either the American Heart Association or European Society of Cardiology.[5-7]

For organisations that make decisions regarding the implementation of population screening programmes, such as the US Preventive Services Task Force and UK National Screening Committee, a clear understanding of the incidence of the target condition provides important context for decision-making.[8 9] In particular, incidence is an indicator of the size of the health problem, and maximum benefit which could be gained from screening, to be balanced against the potential harms such as stopping young people from participating in exercise. Previous reviews of sudden cardiac death incidence have focussed on the incidence across all ranges, or in specific populations.[10 11] The aim of this systematic review is to describe current evidence on the incidence of sudden cardiac death in the young.

Methods

We conducted a systematic review of studies that report the incidence of sudden cardiac death in the young. We were originally commissioned by the UK National Screening Committee to undertake a rapid review of incidence studies.[12] We subsequently chose to develop the rapid review in to this systematic review, with no limitation on study location, date, or publication language.

The review is written in accordance with the Preferred Reporting Items for Systematic Reviews and Meta-Analyses (PRISMA) guidelines. The review is registered with PROSPERO (CRD42019120563).[13]

Search and study selection

We searched MEDLINE (1946-current), EMBASE (1947-current) and the Cochrane library (inception-current) for eligible studies. The search strategy was developed by an information scientist. We, used a combination of keywords and MESH terms to describe the population (e.g. young adult, adolescent), condition (e.g. sudden death, sudden cardiac death), and study type (e.g. cohort, longitudinal) of interest. An example search strategy is included in the electronic supplement.

Following searches and duplicate removal, two reviewers independently screened study titles and abstracts. Conflicts were resolved through discussion or, where needed, arbitration by a third reviewer. The same process was adopted for review of full-text papers. We identified additional studies through forward and backward citation tracking of included studies.

For peer review only - http://bmjopen.bmj.com/site/about/guidelines.xhtml

Study eligibility criteria

We included studies that described the incidence (per 100,000 person-years) of sudden cardiac death in the young, or that provided sufficient data to allow calculation of the incidence. We defined a young individual as someone aged 12-39 years. This age range reflects the target group for a sudden cardiac death programme that was recently considered by the UK National Screening Committee.[12] The lower age cut-off of 12 years was used in the Italian sudden cardiac death screening study.[4] For reasons of pragmatism, we included studies where the reported incidence combined individuals in our target age range with younger individuals (≥ one-year). Exclusion of individuals under one-year avoided the risk of conflating sudden cardiac death with sudden infant death syndrome. We excluded studies where the reported incidence combined individuals in our target age range with older individuals (≥ 40-years), due to the marked increase in sudden cardiac death incidence from the age of 40.[14]

We also excluded studies that reported incidence only in a population with previously known disease, or only included deaths that occurred at certain times of day (e.g. at work or school) or during specific activities (e.g. sports). We imposed no restriction on language or publication date. We did not mandate a specific definition of sudden cardiac death or case-ascertainment process, but these were recorded during data extraction and considered in risk of bias assessments.

Our main population of interest was the general population aged 12-39. To be included in the main analysis, studies were required to report incidence in a general population that included males and females with an age group that spanned at least ten-years between 12 and 39 years. Where studies reported more than one age sub-group (e.g. 12-21 years; 22-31 years), we selected the sub-group closest to the age of mid-late teens to be included in the main analysis. Our planned sub-groups included incidence by age, sex, race, and athletic status (including military personnel). We do not report sub-groups of sub-groups (for example, athletes broken down by sex).

Data extraction and analysis

We used a piloted pro forma to extract population characteristics, study design case identification methods, use of systematic screening, and outcome data. We assessed risk of bias using the Joanna Briggs Institute critical appraisal checklist for prevalence studies.[15] We developed review-specific criteria for each checklist question. Two reviewers independently extracted data and assessed risk of bias, with discrepancies resolved through discussion. Papers published in a language other than English were translated by a fluent speaker.

We extracted incidence per 100,000 person-years and associated 95% confidence interval from the papers. Where these data were not reported, we calculated them from the reported number of sudden cardiac deaths and person follow-up years. We computed the 95% confidence interval based on a binomial distribution, using Stata Version 15.1. Clinical heterogeneity in relation to population characteristics and case ascertainment methods precluded pooling of data.

Patient/ public involvement

We did not involve patients or members of the public in setting the research question, designing the study, undertaking the study, the interpretation of the results, or study write-up.

Results

For peer review only - http://bmjopen.bmj.com/site/about/guidelines.xhtml

We performed initial database searches on 29th April 2019, and updated searches on 16th November 2019. Through searches and citation tracking, we identified 8360 unique citations of which we reviewed the full-text of 238. We excluded 27 of the 65 papers initially included due to substantial or complete data overlap with other studies (figure one).

Most of the 38 included studies[4 16-52] were undertaken in Europe (n=20, 53%) and North America (n=14, 37%) (table one). The majority were retrospective studies (n=29, 76%). Median number of deaths and person follow-up years were 64 (interquartile range 40 to 251) and 6.97 million (interquartile range 2.34 million to 23.70 million) respectively. Study duration ranged from one-year to 41-years.

There was variability in the population, case ascertainment methods, and sudden cardiac death definition used between studies. This methodological heterogeneity precluded meta-analysis. Only nine studies (24%) stated that they used the AHA/ESC definition of sudden cardiac death. Our risk of bias assessment found that most studies were at high risk of bias. (figure two; electronic supplement table S1) As shown in figure two, the main factor contributing to a high risk of bias was the method used to identify cases of sudden cardiac death. In five (13%) studies, sudden cardiac death was identified using death certificates. The remaining studies used a range of approaches that typically incorporated autopsy data to some extent.

Our main analysis included 26 studies (figure three). The median of reported incidence was 1.7 sudden cardiac death per 100,000 person-years (interquartile range (IQR) 1.3 to 2.6, range 0.75 to 11.9). Of the 26 studies, 17 (65%) reported an incidence of less than two sudden cardiac death events per 100,000 person-years, and 14 (54%) studies reported an incidence between one and two cases per 100,000 person-years. All four studies reporting an incidence over three cases per 100,000 person-years were at high risk of bias across multiple domains.

Geographically, incidence appeared highest in African and Asian countries, although this may in part reflect the case ascertainment process and associated risk of bias. These studies also tended to include only older individuals (≥ 18-years).

For our sub-group analysis, sex, age, athletic status, and race were reported in 13, 11, 9, and 1 studies respectively. We observed a consistently higher incidence in males (figure four). In males, median incidence was 2.7 sudden cardiac death per 100,000 person-years (IQR 1.8 to 4.4, range 1.3 to 36), compared with a median incidence in females of 0.9 cases per 100,000 person-years (IQR 0.6 to 1.4, range 0 to 7.0).

The use of different age cut-offs within studies made comparison between studies on the association of incidence of sudden cardiac death with age challenging (electronic supplement figure S1).

Incidence in military personnel was reported in three studies and appeared to be consistently higher than in other populations (electronic supplement figure S2). All three studies were undertaken in American military personnel and reported that individuals received pre-enrolment screening for cardiac conditions. We did not observe a clear difference in incidence between athletic and non-athletic populations. The highest incidence was reported in a prospective study of screened UK soccer players, although the low number of sudden cardiac deaths (8 events) means that the estimate of the incidence is imprecise.

Race was reported in a single American study which broke incidence down in to four categories (African American, White, Hispanic, other). Incidence was highest in the African-American population (electronic supplement figure S3).

For peer review only - http://bmjopen.bmj.com/site/about/guidelines.xhtml

Discussion

In this systematic review, we included 38 studies from five continents that reported incidence of sudden cardiac death in young individuals. We identified significant variability in the reported incidence across studies. In our main analysis, most studies reported an incidence of between one and two sudden cardiac deaths per 100,000 person-years, although reported incidence ranged from 0.75 to 11.9 cases per 100,000 person-years. This variability may reflect differences in population, case-ascertainment methods, and definition used for sudden cardiac death. In pre-defined subgroups, we observed that male sex and increasing age seem to be associated with increased incidence.

Strengths and weaknesses of the study

The key strength of this review was the systematic identification and synthesis of 38 studies from a wide range of settings. We did not restrict inclusion by date and language, with fluent speakers used for translation.

Our review has four key limitations. Firstly, whilst the geographical area covered by index studies was often large with a long recruitment period, the median number of sudden cardiac deaths identified per study was 64 cases. The method used to identify sudden cardiac death was often sub-optimal, such that misclassification bias may have significantly impacted the reported incidence in some studies.[53] Secondly, heterogeneity between studies in terms of the population and case ascertainment method used precluded meta-analysis. Thirdly, particularly for sub-groups, studies often described only the absolute incidence, and did not provide sufficient additional data to calculate the 95% confidence interval. As such, we could not describe the precision of the incidence rate in many studies. Finally, we limited our condition of interest to sudden cardiac death, and did not examine incidence of sudden cardiac arrest. Whilst survival after sudden cardiac arrest is associated with important morbidity, the standard Utstein categorisation of cardiac arrest cause encompasses all medical causes, of which cardiac causes are only a sub-set.[54 55]

Comparison with other studies

Our review builds on two previous systematic reviews that described sudden cardiac death incidence across all age groups or in specific groups of young individuals.[10 11] In line with our review findings, these reviews reported issues across index studies in relation to variability in reported incidence, use of different definitions of sudden cardiac death, and use of different methods to identify cases of sudden cardiac death.

Variability in reported incidence reflects in part the challenge of accurately identifying cases of sudden cardiac death. The method used to identify deaths varied across studies, although most used administrative data sources. Nevertheless, the inclusion of some patient groups, such as in-hospital deaths in patients admitted for a minor procedure, may have varied across studies. The definition of sudden cardiac death is based on both the circumstances and cause of death, such that classification requires knowledge of the cause of death and events leading up to the death.[5 6] Only nine studies used the ESC/AHA definition of sudden cardiac death. Other studies either predated this definition of sudden cardiac death or adopted a different definition to fit the method used to identify cases of sudden cardiac death. Death certificate data provide an efficient way to estimate sudden cardiac

For peer review only - http://bmjopen.bmj.com/site/about/guidelines.xhtml

death incidence. However, their use typically precludes the use of the AHA/ESC definition of sudden cardiac death as the certificate does not include information on the circumstances of the death. As such, studies relying on death certificate data likely over-estimate sudden cardiac death incidence.[19]

Determination of the cause of sudden cardiac death usually requires an autopsy, ideally by a specialist pathologist.[56 57] In included studies, reporting of the proportion of patients that received an autopsy and type of autopsy performed was often unclear. This is reflected in our risk of bias assessments. Legal requirements for autopsy vary between countries. A recent European study reported an autopsy rate in young sudden death of less than 50%, whilst an Australasian study reported an autopsy rate of 100%.[17 45] Where autopsy is not used, cause of death may be misclassified, although the impact of this misclassification on overall incidence is uncertain. In some cases, cause of death may remain uncertain even after autopsy. In these cases, genetic testing of the deceased may be informative in determining both the cause of death and the need for cardiac screening of the victim's family.[17 57 58] In an Australasian study, genetic testing of 113 cases of unexplained sudden cardiac death cases identified cardiac gene variants that were definitely or probably pathogenic in over a quarter of cases.[17]

Studies included in this review spanned a 66-year period (1950 to 2016), although most studies report data collected since 2000. Recent observational studies provide evidence of a decreasing incidence of sudden cardiac death.[25 42 59] A Danish study reported a 3% average annual decrease in sudden cardiac death incidence between 2000 and 2009.[59] Potential explanations for this decrease include improved investigation of inherited cardiac disease, public health strategies to deal with obesity and smoking, and improved response to out-of-hospital cardiac arrest..

Clinical and policy implications

The decision to implement screening programmes in both the UK and USA requires evidence that the benefits of screening outweigh its harms.[9 60] Disease incidence data provides important context for evaluating evidence, particularly in the absence of direct evidence of benefit from randomised controlled trials. In less common conditions, a key concern are the potential harms that may result from false-positive results, and overdiagnosis of disease which may never become symptomatic. Nevertheless, rare disease may still meet criteria for population-based screening conditions, such as maple syrup urine disease in newborns.[61]

The incidence of a condition describes the number of individuals that might benefit from screening. In practice, however, not all young individuals that sustain sudden cardiac death have the potential to benefit from screening.

Firstly, to be effective, screening must reliably identify individuals with disease. In this context, screening must detect a range of structural and electrical cardiac disease. The optimal screening strategy remains uncertain. A wide range of strategies have been described, ranging from those that comprise only a physical examination or medical history to more detailed assessments that include a 12-lead electrocardiogram.[12 62] In programmes where a 12-lead electrocardiogram is collected, a number of assessment criteria have been developed to determine the need for follow-up testing.[63-65] In a prospective cohort study of 11,168 screened teenage footballers in which screening incorporated a physical examination, health questionnaire, echocardiogram, and 12-lead electrocardiogram, six out of eight individuals who subsequently experienced sudden cardiac death did not have disease detected at screening.[23] This highlights a key challenge for policy makers in that, even if they decide to support a screening programme, detailed consideration will need to be given to the screening process and both the financial implications and individual harm that may stem from false-negatives and false-positive screening results.[66 67]

For peer review only - http://bmjopen.bmj.com/site/about/guidelines.xhtml

Secondly, population-level screening is targeted at asymptomatic individuals without a family history of sudden cardiac death. The incidence of sudden cardiac death reported in index studies included both asymptomatic low-risk individuals and individuals at increased risk of sudden cardiac death who, in some countries, may already have access to screening.[6 68-73] A recent study suggested that around one-third of young individuals experienced warning symptoms in the month preceding their sudden cardiac death, indicating the need for clinicians to consider cardiological investigations in symptomatic young individuals.[74] Examples of individuals at increased risk of sudden cardiac death include those with diagnosed cardiac disease, asymptomatic individuals with a family history of sudden cardiac death, and symptomatic individuals without diagnosed cardiac disease. As such, policy makers need to be cognisant that incidence reported in population-based studies will over-estimate the potential benefit of screening programmes which are targeted at low-risk asymptomatic individuals.

Finally, effective screening relies on the individuals willingness to be screened. To date, most studies report screening which, whilst technically voluntary, was mandated for participation in an activity, such as sport. Studies of athletes report young people are supportive of screening.[75 76] In practice, however, uptake of school-based cardiac screening programmes varies from 56% to 79%, with substantial variation by year and school.[77 78]

In conclusion, studies of incidence of sudden cardiac death in young individuals have produced varying estimates of the incidence, although most studies report the incidence to be between one and two cases per 100,000 person-years. In these studies of young individuals, incidence was highest in males and amongst older individuals.

For peer review only

For peer review only - http://bmjopen.bmj.com/site/about/guidelines.xhtml

Table and figure list

Figure one: PRISMA flow chart of study selections

Figure two: Risk of bias assessment

Figure three: Incidence of sudden cardiac death in general population

Figure footnote: Range following study name indicates population age range

Figure four: Incidence of sudden cardiac death in general population- sex sub-groups

Table one: summary of included studies

Author contributions

KC accepts full responsibility for the work and conduct of the study, had access to the data, and controlled the decision to publish.

KC designed the study, acted as reviewer, undertook the analysis, and drafted the manuscript. OP and RF acted as reviewers. PR designed database searches. KP, WB, AC, GDP, JY and STP contributed to study design. All authors contributed to the write up and approved the final version.

Ethics approval

Not required

Data sharing statement

All data relevant to the study are included in the article or uploaded as supplementary information

Funding

This work was supported by Public Health England (Award/Grant number is not applicable). Public Health England originally commissioned a rapid review through the UK National Screening Committee, on which this systematic review was based. Otherwise, Public Health England had no role in study design; collection, analysis, and interpretation of data; in the writing of the report; and in the decision to submit the article for publication. All authors, external and internal, had full access to all of the data (including statistical reports and tables) in the study and can take responsibility for the integrity of the data and the accuracy of the data analysis.

This work was supported by the National Institute for Health Research (NIHR) Applied Research Collaboration (ARC) West Midlands (Award/Grant number is not applicable).

Professor Perkins is supported as an NIHR senior investigator. Professor Taylor-Phillips is supported by an NIHR Career Development Fellowship (CDF-2016-09-018).

The opinions are those of the authors and not the NIHR, the UK National Screening Committee, the NHS or the Department of Health and Social Care.

For peer review only - http://bmjopen.bmj.com/site/about/guidelines.xhtml

Competing interests

All authors have completed the ICMJE uniform disclosure at www.icmje.org/coi_disclosure.pdf and declare: KC, KP, AC, PR, GDP, JY, STP received financial support from Public Health England for the rapid review on which this review was based; no financial relationships with any organisations that might have an interest in the submitted work in the previous three years; KC, JY and GDP have volunteer roles with the International Liaison Committee on Resuscitation, European Resuscitation Council and Resuscitation Council UK.

For peer review only

References

1. World Health Organisation. World Health Statistics 2018: Monitoing health for the SDGs (sustainable development goals). Geneva, 2018. [Available from http://apps.who.int/iris/bitstream/handle/10665/272596/9789241565585-eng.pdf?ua=1 accessed 13/02/2020]

2. Center for Disease Control and Prevention. 10 leading causes of death by age group, United States- 2017. [Available from: https://www.cdc.gov/injury/wisqars/LeadingCauses.html accessed 20/12/2019]

3. Benjamin EJ, Muntner P, Alonso A, et al. Heart Disease and Stroke Statistics- 2019 Update: A Report From the American Heart Association. Circulation 2019;139(10):e56-e528.

4. Corrado D, Basso C, Pavei A, et al. Trends in Sudden Cardiovascular Death in Young Competitive Athletes After Implementation of a Preparticipation Screening Program. JAMA 2006;296(13):1593-601.

5. Al-Khatib SM, Stevenson WG, Ackerman MJ, et al. 2017 AHA/ACC/HRS Guideline for Management of Patients With Ventricular Arrhythmias and the Prevention of Sudden Cardiac Death. Circulation 2018;138(13):e272-e391.

6. Priori SG, Blomström-Lundqvist C, Mazzanti A, et al. 2015 ESC Guidelines for the management of patients with ventricular arrhythmias and the prevention of sudden cardiac death. Eur Heart J 2015;36(41):2793-867.

7. Van Brabandt H, Desomer A, Gerkens S, et al. Harms and benefits of screening young people to prevent sudden cardiac death. BMJ 2016;353:i1156.

8. US Preventive Services Task Force. Procedure Manual 2018 [Available from: https://www.uspreventiveservicestaskforce.org/Page/Name/procedure-manual accessed 08/01/2020]

9. Public Health England. Criteria for appraising the viability, effectiveness and appropriateness of a screening programme. [Available from: https://www.gov.uk/government/publications/evidence-review-criteria-national-screening-programmes/criteria-for-appraising-the-viability-effectiveness-and-appropriateness-of-a-screening-programme accessed 13/02/2020]

10. Harmon KG, Drezner JA, Wilson MG, et al. Incidence of sudden cardiac death in athletes: a state-of-the-art review. Heart 2014;100(16):1227-34.

11. Kong MH, Fonarow GC, Peterson ED, et al. Systematic Review of the Incidence of Sudden Cardiac Death in the United States. JACC 2011;57(7):794-801.

12. Couper K, Poole K, Bradlow W, et al. Sudden cardiac death in the young: External review against programme appraisal criteria for the UK National Screening Committee. [Available from https://legacyscreening.phe.org.uk/policydb_download.php?doc=1239 accessed 17/08/2020]

13. Moher D, Liberati A, Tetzlaff J, et al. Preferred reporting items for systematic reviews and meta-analyses: the PRISMA statement. Ann Intern Med 2009;151(4):264-9.

For peer review only

For peer review only - http://bmjopen.bmj.com/site/about/guidelines.xhtml

14. Risgaard B, Winkel BG, Jabbari R, et al. Burden of sudden cardiac death in persons aged 1 to 49 years: nationwide study in Denmark. Circ Arrhythm Electrophysiol 2014;7(2):205-11.

15. Joanna Briggs Institute. Joanna Briggs Institute Checklist for Prevalence Studies 2017 [Available from: http://joannabriggs.org/assets/docs/critical-appraisal-tools/JBI_Critical_Appraisal-Checklist_for_Prevalence_Studies2017.pdf accessed 17/03/2019].

16. Anastasakis A, Papatheodorou E, Ritsatos K, et al. Sudden unexplained death in the young: epidemiology, aetiology and value of the clinically guided genetic screening. EP Europace 2018;20(3):472-80.

17. Bagnall RD, Weintraub RG, Ingles J, et al. A Prospective Study of Sudden Cardiac Death among Children and Young Adults. NEJM 2016;374(25):2441-52.

18. Bonny A, Tibazarwa K, Mbouh S, et al. Epidemiology of sudden cardiac death in Cameroon: The first population-based cohort survey in sub-Saharan Africa. Int J Epidemiol 2017;46(4):1230-38.

19. Chugh SS, Jui J, Gunson K, et al. Current burden of sudden cardiac death: Multiple source surveillance versus retrospective death certificate-based review in a large U.S. community. JACC 2004;44(6):1268-75.

20. Eckart RE, Shry EA, Burke AP, et al. Sudden death in young adults: An autopsy-based series of a population undergoing active surveillance. JACC 2011;58(12):1254-61.

21. Fragkouli K, Vougiouklakis T. Sudden cardiac death: An 11-year postmortem analysis in the region of Epirus, Greece. Pathol Res Pract 2010;206(10):690-94.

22. Hofer F, Fellmann F, Schlapfer J, et al. Sudden cardiac death in the young (5-39 years) in the canton of Vaud, Switzerland. BMC Cardiovasc Disord 2014;14:140.

23. Malhotra A, Dhutia H, Finocchiaro G, et al. Outcomes of Cardiac Screening in Adolescent Soccer Players. NEJM 2018;379(6):524-34.

24. Papadakis M, Sharma S, Cox S, et al. The magnitude of sudden cardiac death in the young: a death certificate-based review in England and Wales. EP Europace 2009;11(10):1353-8.

25. Wisten A, Krantz P, Stattin EL. Sudden cardiac death among the young in Sweden from 2000 to 2010: an autopsy-based study. EP Europace 2017;19(8):1327-34.

26. Winkel BG, Risgaard B, Bjune T, et al. Gender differences in sudden cardiac death in the young-a nationwide study. BMC Cardiovasc Disord 2017;17:19.

27. Pilmer CM, Kirsh JA, Hildebrandt D, et al. Sudden cardiac death in children and adolescents between 1 and 19 years of age. Heart Rhythm 2014;11(2):239-45.

28. Pilmer CM, Porter B, Kirsh JA, et al. Scope and nature of sudden cardiac death before age 40 in Ontario: a report from the cardiac death advisory committee of the office of the chief coroner. Heart Rhythm 2013;10(4):517-23.

29. Harmon KG, Asif IM, Maleszewski JJ, et al. Incidence, Cause, and Comparative Frequency of Sudden Cardiac Death in National Collegiate Athletic Association Athletes: A Decade in Review. Circulation 2015;132(1):10-9.

30. Harmon KG, Asif IM, Maleszewski JJ, et al. Incidence and Etiology of Sudden Cardiac Arrest and Death in High School Athletes in the United States. Mayo Clin Proc 2016;91(11):1493-502.

For peer review only - http://bmjopen.bmj.com/site/about/guidelines.xhtml

31. Maron BJ, Haas TS, Murphy CJ, et al. Incidence and causes of sudden death in U.S. college athletes. JACC 2014;63(16):1636-43.

32. Maron BJ, Haas TS, Duncanson ER, et al. Comparison of the Frequency of Sudden Cardiovascular Deaths in Young Competitive Athletes Versus Nonathletes: Should We Really Screen Only Athletes? Am J Cardiol 2016;117(8):1339-41.

33. Morentin B, Audicana C. Population-based study of out-of-hospital sudden cardiovascular death: incidence and causes of death in middle-aged adults. Rev Esp Cardiol 2011;64(1):28-34.

34. Zhang J, Zhou X, Xing Q, et al. Epidemiological investigation of sudden cardiac death in multiethnic Xinjiang Uyghur autonomous region in Northwest China. BMC Public Health 2019;19(1):116.

35. Wren C, O'Sullivan JJ, Wright C. Sudden death in children and adolescents. Heart 2000;83(4):410-3.

36. Wisten A, Forsberg H, Krantz P, et al. Sudden cardiac death in 15-35-year olds in Sweden during 1992-99. J Intern Med 2002;252(6):529-36.

37. Vaartjes I, Hendrix A, Hertogh EM, et al. Sudden death in persons younger than 40 years of age: incidence and causes. Eur J Cardiovasc Prev Rehabil 2009;16(5):592-6.

38. Uuskula M, Lamp K, Vali M. An age-related difference in the ratio of sudden coronary death over acute myocardial infarction in Estonian males. J Clin Epidemiol 1998;51(7):577-80.

39. Morentin B, Paz Suarez-Mier M, Audicana C, et al. Incidence and causes of sudden death in persons less than 36 years of age. Med Clin 2001;116(8):281-5.

40. Margey R, Roy A, Tobin S, et al. Sudden cardiac death in 14- to 35-year olds in Ireland from 2005 to 2007: a retrospective registry. EP Europace 2011;13(10):1411-8.

41. Goudevenos JA, Papadimitriou ED, Papathanasiou A, et al. Incidence and other epidemiological characteristics of sudden cardiac death in northwest Greece. Int J Cardiol 1995;49(1):67-75.

42. El-Assaad I, Al-Kindi SG, Aziz PF. Trends of Out-of-Hospital Sudden Cardiac Death Among Children and Young Adults. Pediatrics 2017;140(6):e20171438.

43. Eckart RE, Scoville SL, Campbell CL, et al. Sudden death in young adults: a 25-year review of autopsies in military recruits. Ann Intern Med 2004;141(11):829-34.

44. Driscoll DJ, Edwards WD. Sudden unexpected death in children and adolescents. JACC 1985;5(6):118B-21B.

45. Asatryan B, Vital C, Kellerhals C, et al. Sports-related sudden cardiac deaths in the young population of Switzerland. PLoS ONE 2017;12(3):e0174434.

46. Hua W, Zhang L-F, Wu Y-F, et al. Incidence of Sudden Cardiac Death in China: Analysis of 4 Regional Populations. JACC 2009;54(12):1110-18.

47. Einarsson G, Björnsson J, Gunnarsson G. Sudden cardiac death in the young. A 30 year nation-wide study in Iceland. Int J Cardiol 2007;119:S34-S35.

48. Neuspiel DR, Kuller LH. Sudden and Unexpected Natural Death in Childhood and Adolescence. JAMA 1985;254(10):1321-25.

For peer review only

For peer review only - http://bmjopen.bmj.com/site/about/guidelines.xhtml

49. Drehner D, Neuhauser KM, Neuhauser TS, et al. Death among U.S. Air Force basic trainees, 1956 to 1996. Mil Med 1999;164(12):841-7.

50. Karvouni E, Anastasakis A, Spiliopoulou C, et al. Sudden death in the young in Greece. Hell J Cardiol 2000;41:350-70.

51. Anderson RE, Hill RB, Broudy DW, et al. A population-based autopsy study of sudden, unexpected deaths from natural causes among persons 5 to 39 years old during a 12-year period. Hum Pathol 1994;25(12):1332-40.

52. Perez G, Marrugat J, Sunyer J, et al. Sudden cardiac death in the counties of Girona. Med Clin 1992;99(13):489-92.

53. Delgado-Rodríguez M, Llorca J. Bias. Journal Epidemiol Community Health 2004;58(8):635-41.

54. Lilja G, Nielsen N, Bro-Jeppesen J, et al. Return to Work and Participation in Society After Out-of-Hospital Cardiac Arrest. Circ Cardiovasc Qual Outcomes 2018;11(1):e003566.

55. Perkins GD, Jacobs IG, Nadkarni VM, et al. Cardiac arrest and cardiopulmonary resuscitation outcome reports: update of the Utstein Resuscitation Registry Templates for Out-of-Hospital Cardiac Arrest: a statement for healthcare professionals from a task force of the International Liaison Committee on Resuscitation and the American Heart Association Emergency Cardiovascular Care Committee and the Council on Cardiopulmonary, Critical Care, Perioperative and Resuscitation. Circulation 2015;132(13):1286-300.

56. de Noronha SV, Behr ER, Papadakis M, et al. The importance of specialist cardiac histopathological examination in the investigation of young sudden cardiac deaths. EP Europace 2013;16(6):899-907.

57. Basso C, Aguilera B, Banner J, et al. Guidelines for autopsy investigation of sudden cardiac death: 2017 update from the Association for European Cardiovascular Pathology. Virchows Archiv 2017;471(6):691-705.

58. Lahrouchi N, Raju H, Lodder EM, et al. Utility of Post-Mortem Genetic Testing in Cases of Sudden Arrhythmic Death Syndrome. JACC 2017;69(17):2134-45.

59. Lynge TH, Nielsen JL, Blanche P, et al. Decline in incidence of sudden cardiac death in the young: a 10-year nationwide study of 8756 deaths in Denmark. EP Europace 2019;21(6):909-17.

60. U.S. Preventive Services Task Force. Grade Definitions 2012 [Available from: https://www.uspreventiveservicestaskforce.org/Page/Name/grade-definitions accessed 06/01/2019]

61. UK National Screening Committee. The UK NSC recommendation on Maple Syrup Urine Disease 2014 [Available from: https://legacyscreening.phe.org.uk/msud accessed 21st January 2020]

62. Harmon KG, Zigman M, Drezner JA. The effectiveness of screening history, physical exam, and ECG to detect potentially lethal cardiac disorders in athletes: a systematic review/meta-analysis. J Electrocardiol 2015;48(3):329-38.

63. Riding NR, Sheikh N, Adamuz C, et al. Comparison of three current sets of electrocardiographic interpretation criteria for use in screening athletes. Heart 2015;101(5):384-90.

For peer review only - http://bmjopen.bmj.com/site/about/guidelines.xhtml

64. Brosnan M, La Gerche A, Kalman J, et al. The Seattle Criteria increase the specificity of preparticipation ECG screening among elite athletes. Br J Sports Med 2014;48(15):1144-50.

65. Dunn TP, Pickham D, Aggarwal S, et al. Limitations of Current AHA Guidelines and Proposal of New Guidelines for the Preparticipation Examination of Athletes. Clin J Sport Med 2015;25(6):472-77.

66. Dhutia H, Malhotra A, Yeo TJ, et al. Inter-Rater Reliability and Downstream Financial Implications of Electrocardiography Screening in Young Athletes. Circ Cardiovasc Qual Outcomes 2017;10(8):e003306.

67. Dhutia H, Malhotra A, Gabus V, et al. Cost Implications of Using Different ECG Criteria for Screening Young Athletes in the United Kingdom. J Am Coll Cardiol 2016;68(7):702-11.

68. Department of Health. National Service Framework for Coronary Heart Disease- Chapter Eight: Arrhythmias and Sudden Cardiac Death London: Department of Health, 2005. [Available from: https://webarchive.nationalarchives.gov.uk/20100420225444/http://www.dh.gov.uk/en/Healthcare/Longtermconditions/Vascular/Coronaryheartdisease/DH_4117048 accessed 13/02/2020]

69. National Institute for Health and Care Excellence. Clinical Guideline 109: Transient loss of consciousness ('blackouts') in over 16s 2014 [Available from: https://www.nice.org.uk/guidance/cg109 accessed 18/03/2019]

70. Brignole M, Moya A, de Lange FJ, et al. 2018 ESC Guidelines for the diagnosis and management of syncope. Eur Heart J 2018;39(21):1883-948.

71. Norrish G, Field E, Mcleod K, et al. Clinical presentation and survival of childhood hypertrophic cardiomyopathy: a retrospective study in United Kingdom. Eur Heart J 2018;40(12):986-93.

72. Alexander PMA, Nugent AW, Daubeney PEF, et al. Long-Term Outcomes of Hypertrophic Cardiomyopathy Diagnosed During Childhood. Circulation 2018;138(1):29-36.

73. Ranthe MF, Winkel BG, Andersen EW, et al. Risk of cardiovascular disease in family members of young sudden cardiac death victims. Eur Heart J 2013;34(7):503-11.

74. Jayaraman R, Reinier K, Nair S, et al. Risk Factors of Sudden Cardiac Death in the Young. Circulation 2018;137(15):1561-70.

75. Asif IM, Annett S, Ewing JA, et al. Psychological impact of electrocardiogram screening in National Collegiate Athletic Association athletes. Br J Sports Med 2017;51(20):1489-92.

76. Asif IM, Johnson S, Schmieg J, et al. The psychological impact of cardiovascular screening: the athlete's perspective. Br J Sports Med 2014;48(15):1162-6.

77. Marek J, Bufalino V, Davis J, et al. Feasibility and findings of large-scale electrocardiographic screening in young adults: data from 32,561 subjects. Heart Rhythm 2011;8(10):1555-9.

78. Vilardell P, Brugada J, Aboal J, et al. Characterization of electrocardiographic findings in young students. Rev Esp Cardiol 2020;73(2):139-44.

For peer review only - http://bmjopen.bmj.com/site/about/guidelines.xhtml

Table one:

	Design	Setting	Population: Type/ Age	SCD cases	Follow-up years	Systematic screening	SCD definition	Case ascertainment	
								Identification of deaths	Main method to determine SCD
Anastasakis 2018	PC	Greece 2002-2010	General 1-35	226	12,750,000	No	AHA/ ESC	Government records/ death certificates	Autopsy record review
Anderson 1994	RC	USA 1977-1988	General 5-39	183	9,836,196	No	Other	Medical examiner records	Autopsy record review
Asatryan 2017	RC	Switzerland 1999-2010	General 10-39	349[†]	25,344,456	Athletes only	Other	Forensic medicine database	Autopsy record review
Bagnall 2016	PC	Austrasia 2010-2012	General 1-35	490	37,770,000	No	AHA/ ESC	Government records/ death certificates; Autopsy records	Autopsy record review
Bonny 2017	PC	Cameroon 2013	General 18-39	-	-	No	Other	Government records/ death certificates	Medical record review; interviews
Chugh 2004	PC	USA 2002-2003	General 10-34	-	-	No	AHA/ ESC	Hospital/ ambulance/ medical examiner records; death certificates	Autopsy/ medical record review; ICD code
Corrado 2006	PC	Italy 1979-2004	General 12-35	320	36,144,100	Athletes only	Other	Medical centre registers, media searches (prospective registry)	Autopsy record review
Drehner 1999	RC	USA 1956-1996	Military 17-26	23	340,990	Yes	Not stated	Military records	Autopsy record review
Driscoll 1985	RC	USA 1950-1982	General 1-22	7	930,678	No	Other	Government records/ death certificates	Autopsy/ medical record review
Eckart 2004	RC	USA 1977-2001	Militrary 17-35	64	-	Yes	Other	Military records	Autopsy/ military record review
Eckart 2011	RC	USA 1998-2008	Military <20	-	-	Yes	Other	Military records	Autopsy record review
Einarsson 2007	RC	Iceland 1974-2004	General 12-35	42	-	No	Not stated	Government records/ death certificates	Autopsy/ medical record review
El-Assaad 2017	RC	USA 1999-2015	General 1-34	31492	-	No	Other	Government records/ death certificates	ICD code
Fragkouli 2010	RC	Greece 1998-2008	General 1-35	28	-	No	Other	Forensic laboratory records	Autopsy/ medical/ police record review
Goudevenos 1995	PC	Greece 1990-1993	General 30-39	-	-	No	Other	Hospital/ coroner records; Department of statistics	Autopsy/ medical record review; interviews
Harmon 2015	RC	USA 2003-2013	Athletes 17-24	79	4,242,519	Yes	AHA/ ESC	Media search; NCAA records	Autopsy record review; interviews
Harmon 2016	RC	USA 2007-2013	Athletes 14-18	69	6,974,640	Yes	Other	Media search	Autopsy record review
Hofer 2014	RC	Switzerland 2000-2007	General 5-39	40	2,340,368	No	Not stated	Government records/ death certificates	ICD code
Hua 2009	PC	China 2005-2006	General 25-34	9	115,188	No	Other	Government records/ death certificates	Medical record review; interviews

For peer review only - http://bmjopen.bmj.com/site/about/guidelines.xhtml

Karvouni 2000	RC	Greece 1997-1999	General 1-35	33‡	4,033,333	No	Other	Forensic laboratory, hospital and ambulance records	Autopsy record review
Malhotra 2018	PC	UK 1996-2016	Athletes 15-17	8	118,351	Yes	Not stated	Voluntary reports/ survey; media search	Autopsy record review
Margey 2011	RC	Ireland 2005-2007	General 15-34	116	4,065,513	No	AHA/ ESC	Government records/ death certificates	Autopsy record review
Maron 2014	RC	USA 2002-2011	Athletes 17-26	64	4,052,369	No	Not stated	Media search; NCAA records	Autopsy record review
Maron 2016	RC	USA 2000-2014	General 14-23	27	1,308,730	No	Not stated	Medical examiner records	Autopsy record review
Morentin 2001	RC	Spain 1991-1998	General 1-35	46	4,413,088	No	Other	Forensic laboratory records	Autopsy record review
Morentin 2011	RC	Spain 2003-2008	General 35-39	42	-	No	AHA/ ESC	Government records/ death certificates	Autopsy record review
Neuspiel 1985	RC	USA 1972-1980	General 1-21	51	-	No	Other	Government records/ death certificates	Autopsy/ medical record review
Papadakis 2009	RC	UK 2002-2005	General 1-34	1677	94,256,200	No	Other	Government records/ death certificates	ICD code
Perez 1992	PC	Spain 1987-1988	General 25-34	1	-	No	Not stated	Government records/ death certificates	Medical record review; interviews
Pilmer 2013	RC	Canada 2008	General 2-29	56	-	No	Other	Coroner records	Autopsy/ medical/ police record review
Pilmer 2014	RC	Canada 2005-2009	General 1-19	116	14,893,860	No	Other	Coroner records	Autopsy record review
Uuskula 1998	RC	Estonia 1980-1996	General 20-39	251	902,241	No	Other	Government records/ death certificates	Autopsy record review
Vaartjes 2009	RC	Holland 1996-2006	General 1-39	1500	92,374,043	No	Other	Government records/ death certificates	ICD code
Winkel 2017	RC	Denmark 2000-2009	General 1-35	635	23,700,000	No	AHA/ ESC	Government records/ death certificates	Autopsy record review
Wisten 2002	RC	Sweden 1992-1999	General 15-35	181	19,514,080	No	AHA/ ESC	Government records/ national forensic medicine database	Autopsy/ medical record review
Wisten 2017	RC	Sweden 2000-2010	General 1-35	552	42,900,000	Athletes only	AHA/ ESC	Government records/ national forensic medicine database	Autopsy record review
Wren 2000	RC	England 1985-1994	General 1-20	59	8,060,000	No	Other	Coroner/ hospital records	Death certificate
Zhang 2019	RC	China 2015	General 18-35	-	-	No	Other	Government records/ death certificates	Medical record review; interviews

PC- Prospective cohort; RC- Retrospective cohort. AHA/ESC- American Heart Association/ European Society of Cardiology. NCAA National Collegiate Athletic Association. SCD- Sudden cardiac death.
† Study inflated number of SCDs to account for low autopsy rate. ‡- Unable to replicate reported incidence based on study data.

For peer review only

For peer review only - http://bmjopen.bmj.com/site/about/guidelines.xhtml

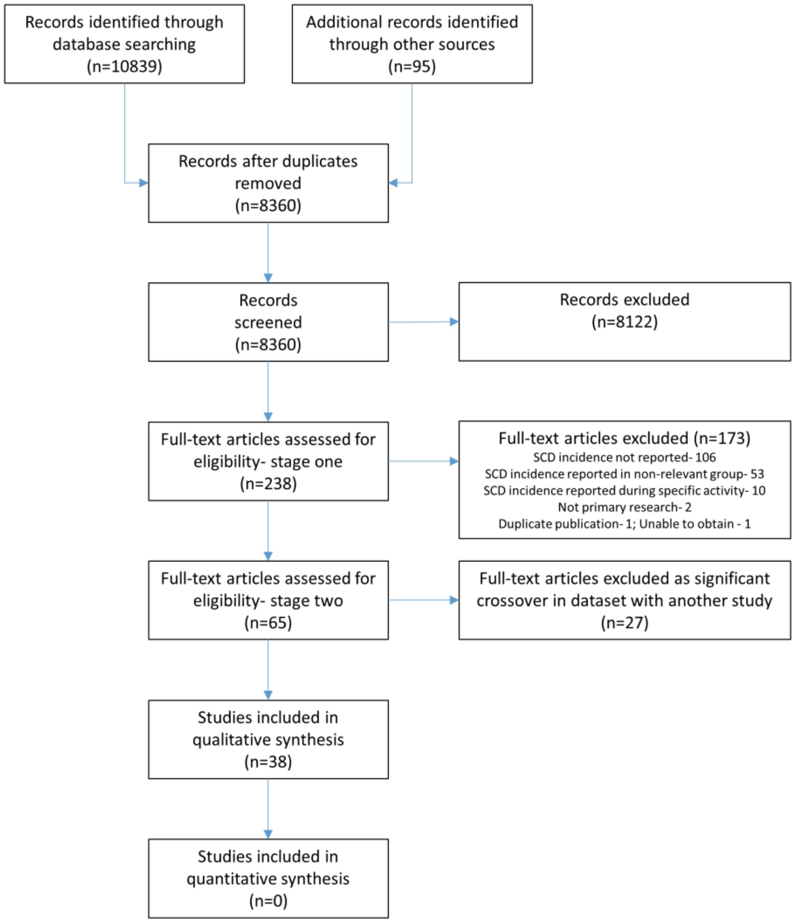

PRISMA flow chart of study selections

190x275mm (300 x 300 DPI)

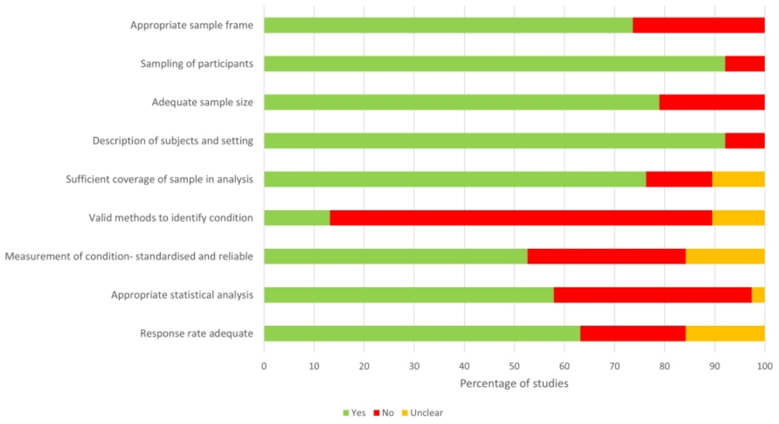

Risk of bias assessment

338x190mm (300 x 300 DPI)

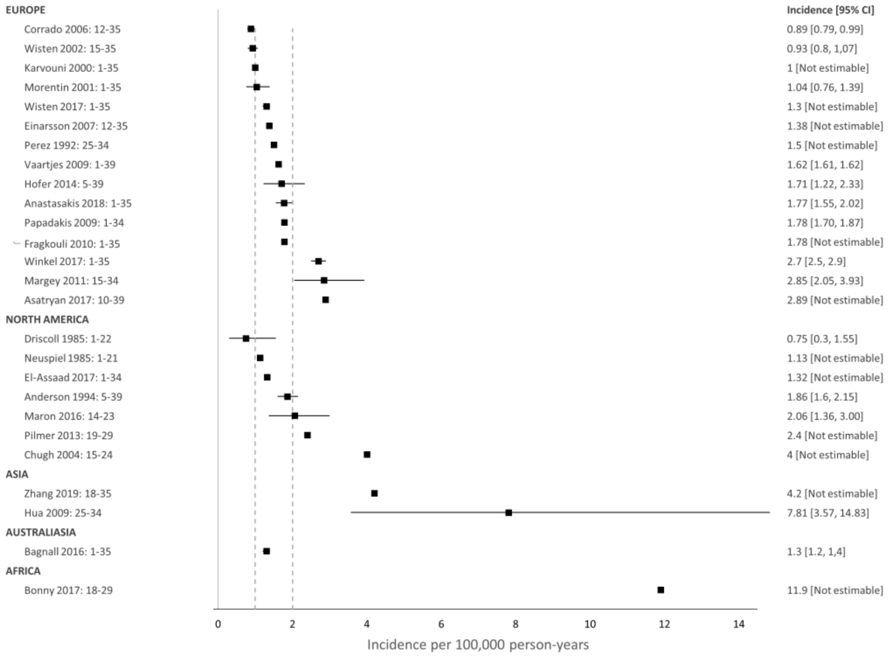

Incidence of sudden cardiac death in general population

Figure footnote: Range following study name indicates population age range

275x190mm (300 x 300 DPI)

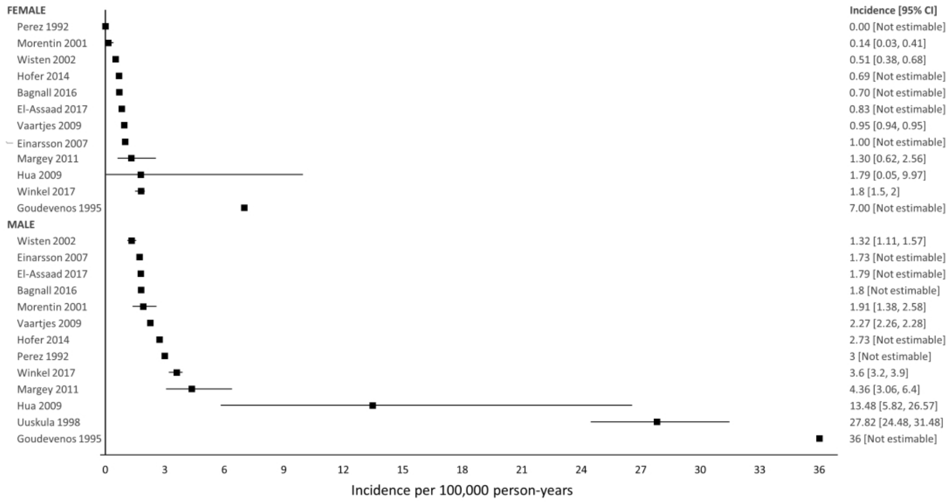

Incidence of sudden cardiac death in general population- sex sub-groups

275x190mm (300 x 300 DPI)

Incidence of sudden cardiac death in the young: a systematic review- Supplementary material

Contents:

MEDLINE search strategy- page 1

Table S1: study risk of bias assessment- page 2

Figure S1: Sudden cardiac death incidence by age- page 3

Figure S2: Sudden cardiac death incidence by athletic/ military status- page 4

Figure S3: Sudden cardiac death incidence by race- page 5

For peer review only

MEDLINE search strategy

1 exp Death, Sudden, Cardiac/
2 (sudden adj3 death).mp
3 cardiac death.mp
4 1 or 2 or 3
5 exp Incidence/
6 incidence.mp
7 exp Prevalence/
8 prevalence.mp
9 5 or 6 or 7 or 8
10 (adolescen* or young* or student* or athlet* or soccer or football*).mp
11 exp Young Adult/ or exp Adult/
12 exp Adolescent/
13 exp Child/
14 10 or 11 or 12 or 13
15 exp Cohort Studies/
16 exp Longitudinal Studies/
17 exp Follow-Up Studies/
18 exp Prospective Studies/
19 exp Retrospective Studies/
20 (longitudinal or prospective* or observational or registry).mp
21 (autopsy or death certificate*).mp
22 15 or 16 or 17 or 18 or 19 or 20 or 21
23 4 and 9 and 14 and 22
24 limit 23 to (case reports or comment or editorial or letter)
25 23 not 24

For peer review only - http://bmjopen.bmj.com/site/about/guidelines.xhtml

Table S1: study risk of bias assessment

	1-Appropriate sample frame	2-Sampling of participants	3-Adequate sample size	4-Description of subjects and setting	5-Sufficient coverage of sample in analysis	6-Valid methods to identify condition	7-Measurement of condition-standardised and reliable	8-Appropriate statistical analysis	9-Response rate adequate
Anastasakis 2018	Y	Y	Y	Y	Y	Y	Y	Y	Y
Anderson 1994	Y	Y	Y	Y	Y	N	Y	Y	Y
Asatryan 2017	Y	Y	Y	Y	Y	N	N	N	N
Bagnall 2016	Y	Y	Y	Y	Y	Y	Y	Y	Y
Bonny 2017	Y	N	N	N	U/C	N	Y	N	Y
Chugh 2004	Y	Y	N	Y	Y	N	N	N	Y
Corrado 2006	Y	Y	Y	Y	N	N	Y	Y	Y
Drehner 1999	N	Y	Y	Y	Y	N	U/C	N	N
Driscoll 1985	Y	Y	N	Y	Y	N	N	Y	Y
Eckart 2004	N	Y	Y	Y	Y	N	Y	Y	Y
Eckart 2011	N	Y	Y	Y	Y	N	Y	Y	N
Einarsson 2007	Y	Y	Y	Y	Y	U/C	U/C	U/C	U/C
El-Assaad 2017	Y	Y	Y	Y	Y	N	Y	N	Y
Fragkouli 2010	Y	Y	Y	Y	U/C	N	Y	N	U/C
Goudevenos 1995	N	Y	N	Y	Y	N	N	N	N
Harmon 2016	N	Y	Y	Y	N	N	N	Y	N
Harmon 2015	N	Y	Y	Y	N	N	N	Y	U/C
Hofer 2014	Y	Y	Y	Y	Y	N	N	Y	Y
Hua 2009	Y	N	N	N	N	N	N	Y	Y
Karvouni 2000	Y	Y	Y	Y	U/C	N	Y	N	U/C
Malhotra 2018	N	Y	N	Y	Y	U/C	Y	Y	Y
Margey 2011	Y	Y	Y	Y	Y	Y	Y	Y	N
Maron 2016	Y	Y	Y	Y	Y	U/C	U/C	Y	Y
Maron 2014	N	Y	Y	Y	N	U/C	Y	Y	N
Morentin 2001	Y	Y	Y	Y	Y	N	Y	Y	Y
Morentin 2011	N	Y	N	Y	Y	Y	U/C	N	Y
Neuspiel 1985	Y	Y	Y	Y	Y	N	N	N	Y
Papadakis 2009	Y	Y	Y	Y	Y	N	Y	Y	Y
Perez 1992	Y	Y	N	Y	Y	N	U/C	N	Y
Pilmer 2013	Y	Y	Y	Y	U/C	N	Y	N	U/C
Pilmer 2014	Y	Y	Y	Y	Y	N	Y	N	Y
Uuskula 1998	N	Y	Y	Y	Y	N	U/C	Y	U/C
Vaartjes 2009	Y	Y	Y	Y	Y	N	Y	Y	Y
Winkel 2017	Y	Y	Y	Y	Y	N	N	Y	Y
Wisten 2002	Y	Y	Y	Y	Y	Y	N	N	Y
Wisten 2017	Y	Y	Y	Y	Y	N	N	Y	N
Wren 2000	Y	Y	Y	Y	Y	N	Y	Y	Y
Zhang 2019	Y	N	Y	N	Y	N	Y	N	Y

Key: Y- Yes; N- No; U/C- Unclear

Full list of questions:

1. Was the sample frame appropriate to address the target population?
2.Were study participants sampled in an appropriate way?
3.Was the sample size adequate?
4.Were the study subjects and the setting described in detail?
5.Was the data analysis conducted with sufficient coverage of the identified sample?
6.Were valid methods used for the identification of the condition?
7.Was the condition measured in a standard, reliable way for all participants?
8.Was there appropriate statistical analysis?
9.Was the response rate adequate, and if not, was the low response rate managed appropriately?

For peer review only - http://bmjopen.bmj.com/site/about/guidelines.xhtml

Figure S1: Sudden cardiac death incidence by age

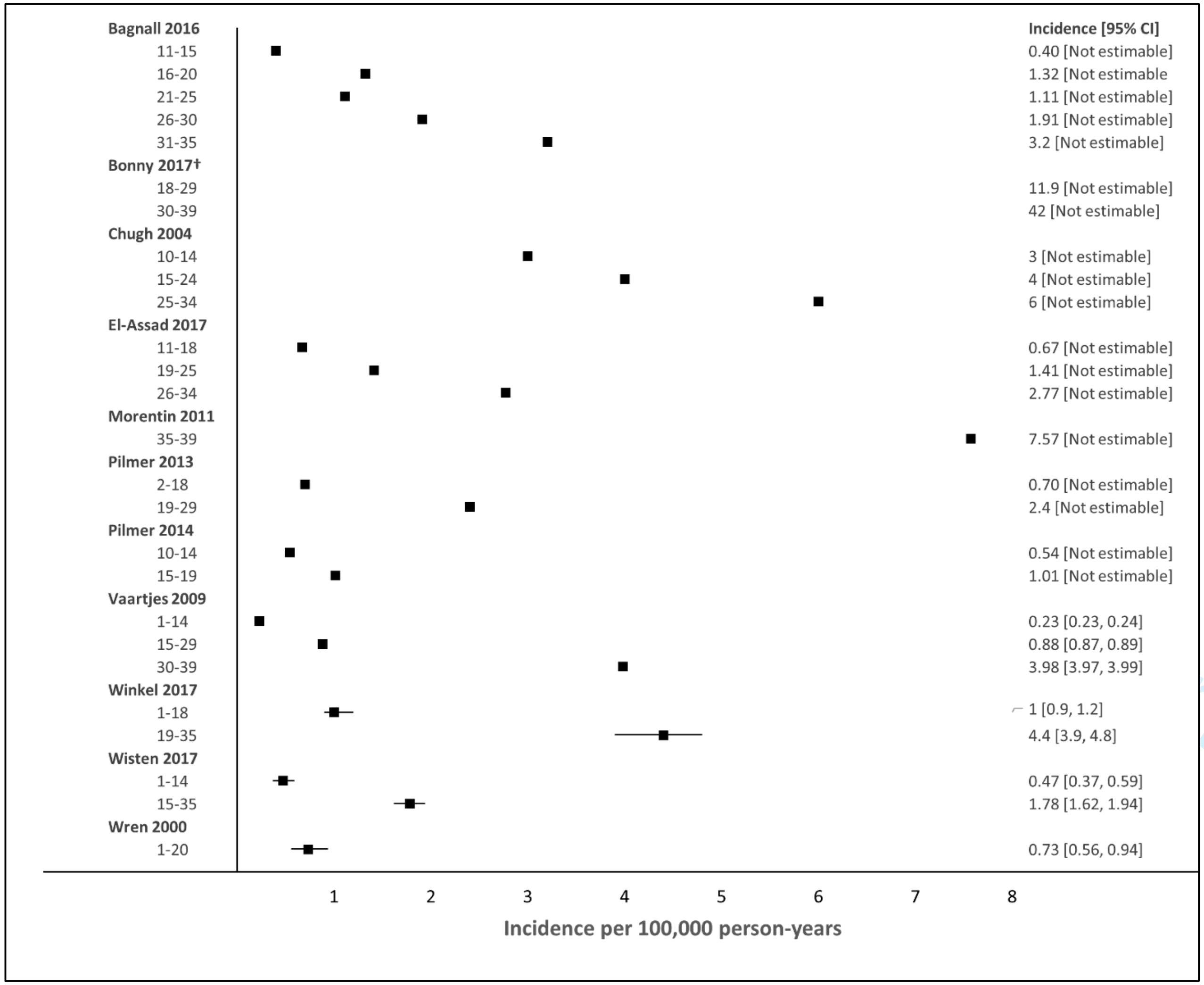

†- Data not presented on chart as significant outlier

For peer review only - http://bmjopen.bmj.com/site/about/guidelines.xhtml

Figure S2: Sudden cardiac death incidence by athletic/ military status

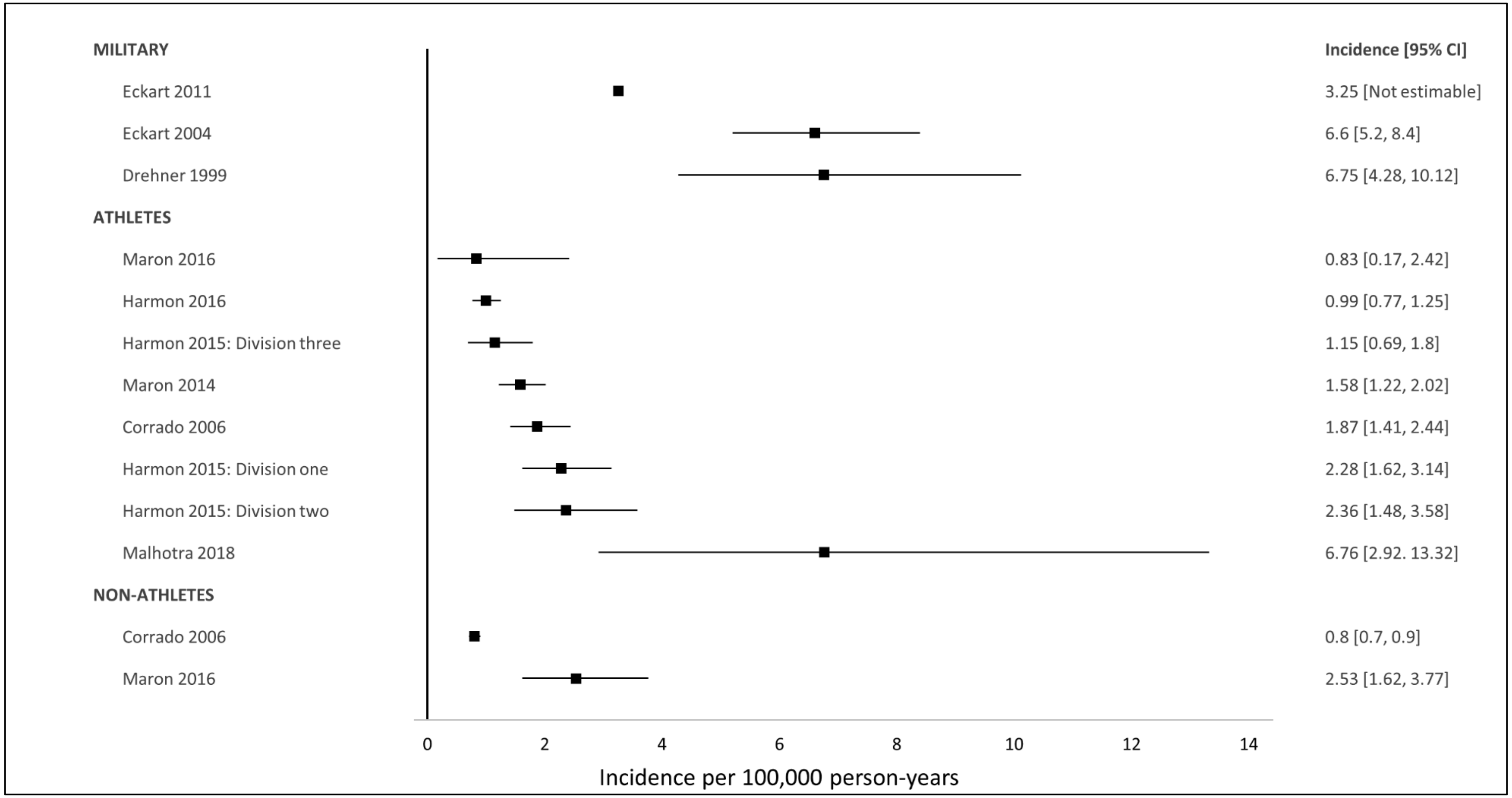

Figure S3: Sudden cardiac death incidence by race

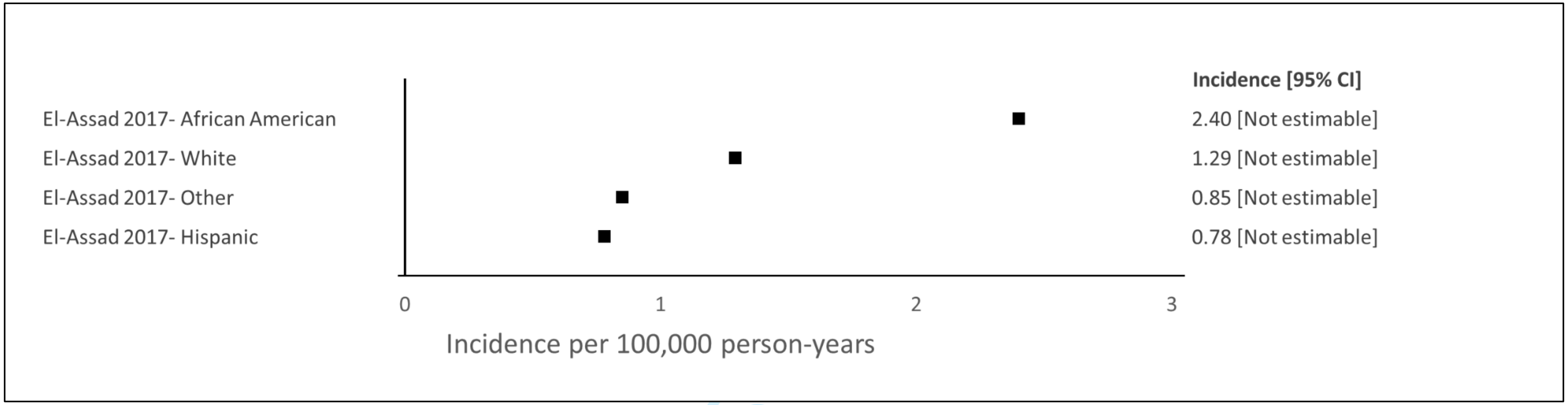

For peer review only - http://bmjopen.bmj.com/site/about/guidelines.xhtml

PRISMA 2009 Checklist

Section/topic	#	Checklist item	Reported on page #
**TITLE**			
Title	1	Identify the report as a systematic review, meta-analysis, or both.	1
**ABSTRACT**			
Structured summary	2	Provide a structured summary including, as applicable: background; objectives; data sources; study eligibility criteria, participants, and interventions; study appraisal and synthesis methods; results; limitations; conclusions and implications of key findings; systematic review registration number.	2
**INTRODUCTION**			
Rationale	3	Describe the rationale for the review in the context of what is already known.	4
Objectives	4	Provide an explicit statement of questions being addressed with reference to participants, interventions, comparisons, outcomes, and study design (PICOS).	4
**METHODS**			
Protocol and registration	5	Indicate if a review protocol exists, if and where it can be accessed (e.g., Web address), and, if available, provide registration information including registration number.	4
Eligibility criteria	6	Specify study characteristics (e.g., PICOS, length of follow-up) and report characteristics (e.g., years considered, language, publication status) used as criteria for eligibility, giving rationale.	5
Information sources	7	Describe all information sources (e.g., databases with dates of coverage, contact with study authors to identify additional studies) in the search and date last searched.	4
Search	8	Present full electronic search strategy for at least one database, including any limits used, such that it could be repeated.	Electronic supplement
Study selection	9	State the process for selecting studies (i.e., screening, eligibility, included in systematic review, and, if applicable, included in the meta-analysis).	4
Data collection process	10	Describe method of data extraction from reports (e.g., piloted forms, independently, in duplicate) and any processes for obtaining and confirming data from investigators.	5
Data items	11	List and define all variables for which data were sought (e.g., PICOS, funding sources) and any assumptions and simplifications made.	5
Risk of bias in individual studies	12	Describe methods used for assessing risk of bias of individual studies (including specification of whether this was done at the study or outcome level), and how this information is to be used in any data synthesis.	5
Summary measures	13	State the principal summary measures (e.g., risk ratio, difference in means).	5
Synthesis of results	14	Describe the methods of handling data and combining results of studies, if done, including measures of consistency (e.g., I^2) for each meta-analysis.	N/A

For peer review only

For peer review only - http://bmjopen.bmj.com/site/about/guidelines.xhtml

PRISMA 2009 Checklist

Section/topic	#	Checklist item	Reported on page #
Risk of bias across studies	15	Specify any assessment of risk of bias that may affect the cumulative evidence (e.g., publication bias, selective reporting within studies).	N/A
Additional analyses	16	Describe methods of additional analyses (e.g., sensitivity or subgroup analyses, meta-regression), if done, indicating which were pre-specified.	5
**RESULTS**			
Study selection	17	Give numbers of studies screened, assessed for eligibility, and included in the review, with reasons for exclusions at each stage, ideally with a flow diagram.	5-6/ figure one
Study characteristics	18	For each study, present characteristics for which data were extracted (e.g., study size, PICOS, follow-up period) and provide the citations.	Table one
Risk of bias within studies	19	Present data on risk of bias of each study and, if available, any outcome level assessment (see item 12).	Figure two/ electronic supplement
Results of individual studies	20	For all outcomes considered (benefits or harms), present, for each study: (a) simple summary data for each intervention group (b) effect estimates and confidence intervals, ideally with a forest plot.	Figure 3
Synthesis of results	21	Present results of each meta-analysis done, including confidence intervals and measures of consistency.	N/A
Risk of bias across studies	22	Present results of any assessment of risk of bias across studies (see Item 15).	N/A
Additional analysis	23	Give results of additional analyses, if done (e.g., sensitivity or subgroup analyses, meta-regression [see Item 16]).	Figure 4/ electronic supplemet
**DISCUSSION**			
Summary of evidence	24	Summarize the main findings including the strength of evidence for each main outcome; consider their relevance to key groups (e.g., healthcare providers, users, and policy makers).	7-8
Limitations	25	Discuss limitations at study and outcome level (e.g., risk of bias), and at review-level (e.g., incomplete retrieval of identified research, reporting bias).	7
Conclusions	26	Provide a general interpretation of the results in the context of other evidence, and implications for future research.	7-8
**FUNDING**			
Funding	27	Describe sources of funding for the systematic review and other support (e.g., supply of data); role of funders for the systematic review.	10

For peer review only

PRISMA 2009 Checklist

From: Moher D, Liberati A, Tetzlaff J, Altman DG, The PRISMA Group (2009). Preferred Reporting Items for Systematic Reviews and Meta-Analyses: The PRISMA Statement. PLoS Med 6(7): e1000097. doi:10.1371/journal.pmed1000097

For more information, visit: **www.prisma-statement.org**.

For peer review only

BMJ Open

Incidence of sudden cardiac death in the young: a systematic review

Journal:	*BMJ Open*
Manuscript ID	bmjopen-2020-040815.R2
Article Type:	Original research
Date Submitted by the Author:	28-Aug-2020
Complete List of Authors:	Couper, Keith; University of Warwick Warwick Medical School, Warwick Medical School; University Hospitals Birmingham NHS Foundation Trust, Critical Care Unit<br>Putt, Oliver; University of Warwick Warwick Medical School, Warwick Medical School<br>Field, Richard; University Hospitals Birmingham NHS Foundation Trust, Critical Care Unit<br>Poole, Kurtis; University of Warwick Warwick Medical School, Warwick Medical School<br>Bradlow, William; University Hospitals Birmingham NHS Foundation Trust, Department of cardiology<br>Clarke, Aileen; University of Warwick Warwick Medical School, Warwick Medical School<br>Perkins, Gavin; University of Warwick Warwick Medical School, Warwick Medical School; University Hospitals Birmingham NHS Foundation Trust, Critical Care Unit<br>Royle, Pamela; University of Warwick Warwick Medical School, Warwick Medical School<br>Yeung, Joyce ; University of Warwick Warwick Medical School, Warwick Medical School; University Hospitals Birmingham NHS Foundation Trust, Critical Care Unit<br>Taylor-Phillips, Sian; University of Warwick Warwick Medical School, Warwick Medical School
**Primary Subject Heading**:	Epidemiology
Secondary Subject Heading:	Epidemiology, Cardiovascular medicine
Keywords:	Cardiac Epidemiology < CARDIOLOGY, EPIDEMIOLOGY, Adult cardiology < CARDIOLOGY, Paediatric cardiology < PAEDIATRICS

SCHOLARONE™
Manuscripts

I, the Submitting Author has the right to grant and does grant on behalf of all authors of the Work (as defined in the below author licence), an exclusive licence and/or a non-exclusive licence for contributions from authors who are: i) UK Crown employees; ii) where BMJ has agreed a CC-BY licence shall apply, and/or iii) in accordance with the terms applicable for US Federal Government officers or employees acting as part of their official duties; on a worldwide, perpetual, irrevocable, royalty-free basis to BMJ Publishing Group Ltd ("BMJ") its licensees and where the relevant Journal is co-owned by BMJ to the co-owners of the Journal, to publish the Work in this journal and any other BMJ products and to exploit all rights, as set out in our licence.

The Submitting Author accepts and understands that any supply made under these terms is made by BMJ to the Submitting Author unless you are acting as an employee on behalf of your employer or a postgraduate student of an affiliated institution which is paying any applicable article publishing charge ("APC") for Open Access articles. Where the Submitting Author wishes to make the Work available on an Open Access basis (and intends to pay the relevant APC), the terms of reuse of such Open Access shall be governed by a Creative Commons licence – details of these licences and which Creative Commons licence will apply to this Work are set out in our licence referred to above.

Other than as permitted in any relevant BMJ Author's Self Archiving Policies, I confirm this Work has not been accepted for publication elsewhere, is not being considered for publication elsewhere and does not duplicate material already published. I confirm all authors consent to publication of this Work and authorise the granting of this licence.

For peer review only

Incidence of sudden cardiac death in the young: a systematic review

Keith Couper, Oliver Putt, Richard Field, Kurtis Poole, William Bradlow, Aileen Clarke, Gavin D Perkins, Pamela Royle, Joyce Yeung, Sian Taylor-Phillips

Keith Couper, Assistant Professor- k.couper@warwick.ac.uk
Warwick Medical School, University of Warwick, Coventry, UK
Critical Care Unit, University Hospitals Birmingham NHS Foundation Trust, Birmingham, UK

Oliver Putt, Academic Foundation Doctor- oliver.putt@nhs.net
Warwick Medical School, University of Warwick, Coventry, UK

Richard Field, Registrar- richiefield@doctors.org.uk
Critical Care Unit, University Hospitals Birmingham NHS Foundation Trust, Birmingham, UK

Kurtis Poole, Research Associate- kurtis.poole@nhs.net
Warwick Medical School, University of Warwick, Coventry, UK

William Bradlow, Consultant- William.bradlow@uhb.nhs.uk
Department of Cardiology, University Hospitals Birmingham NHS Foundation Trust, Birmingham, UK

Aileen Clarke, Professor- aileen.clarke@warwick.ac.uk
Warwick Medical School, University of Warwick, Coventry, UK

Gavin D Perkins, Professor,- g.d.perkins@warwick.ac.uk
Warwick Medical School, University of Warwick, Coventry, UK
Critical Care Unit, University Hospitals Birmingham NHS Foundation Trust, Birmingham, UK

Pamela Royle, Research Fellow- p.l.royle@warwick.ac.uk
Warwick Medical School, University of Warwick, Coventry, UK

Joyce Yeung, Associate Professor- j.yeung.4@warwick.ac.uk
Warwick Medical School, University of Warwick, Coventry, UK
Critical Care Unit, University Hospitals Birmingham NHS Foundation Trust, Birmingham, UK

Sian Taylor-Phillips, Professor- s.taylor-phillips@warwick.ac.uk
Warwick Medical School, University of Warwick, Coventry, UK

Corresponding author: Dr Keith Couper, Warwick Clinical Trials Unit, Warwick Medical School, University of Warwick, Gibbet Hill Road, Coventry, CV4 7AL
Tel: 02476 151179. Email: k.couper@warwick.ac.uk

For peer review only

For peer review only - http://bmjopen.bmj.com/site/about/guidelines.xhtml

Abstract

Objective: To summarise studies describing incidence of sudden cardiac death in a general population of young individuals to inform screening policy.

Design: Systematic review.

Data sources: Database searches of MEDLINE, EMBASE, and the Cochrane library (all inception to current) on 29th April 2019 (updated 16th November 2019), and forward/ backward citation tracking of eligible studies.

Study eligibility criteria: All studies that reported incidence of sudden of cardiac death in young individuals (12-39 years) in a general population, with no restriction on language or date. Planned sub-groups were incidence by age, sex, race, and athletic status (including military personnel).

Data extraction: Two reviewers independently assessed study eligibility, extracted study data, and assessed risk of bias using the Joanna Briggs Institute critical appraisal checklist for prevalence studies.

Analysis: Reported incidence of sudden cardiac death in the young per 100,000 person-years.

Results: 38 studies that reported incidence across five continents. We identified substantial heterogeneity in population, sudden cardiac death definition, and case ascertainment methods, precluding meta-analysis. Median reported follow-up years was 6.97 million (interquartile range 2.34 million to 23.70 million) and number of sudden cardiac death cases was 64 (interquartile range 40 to 251). In the general population, the median of reported incidence was 1.7 sudden cardiac death per 100,000 person-years (interquartile range (IQR) 1.3 to 2.6, range 0.75 to 11.9). Most studies (n=14, 54%) reported an incidence between one and two cases per 100,000 person-years. Incidence was higher in males and older individuals.

Conclusions: This systematic review identified variability in the reported incidence of sudden cardiac death in the young across studies. Most studies reported an incidence between one and two cases per 100,000 person-years.

PROSPERO registration PROSPERO (CRD42019120563).

Keywords: sudden cardiac death; epidemiology; incidence; systematic review

Word count: 2977

For peer review only

Strengths and limitations of this study

- This systematic review is reported in accordance with the PRISMA (Preferred Reporting Items for Systematic Reviews and Meta-Analyses) checklist.
- We identified studies that reported incidence of sudden cardiac death in the young through searches of key databases and citation tracking
- All eligible studies were included, irrespective of publication date or publication language.
- We identified and described variability in the definition of sudden cardiac death and methods used to identify sudden cardiac death across studies.
- We did not include cases of out-of-hospital cardiac arrest with successful resuscitation.

For peer review only

For peer review only - http://bmjopen.bmj.com/site/about/guidelines.xhtml

Introduction

Cardiovascular disease is the leading cause of death worldwide.[1] In the young, however, deaths due to cardiovascular disease are much less frequent than deaths due to other causes such as unintentional injury, suicide, and homicide.[2] Nevertheless, over 20,000 young individuals aged under 45 die due to cardiovascular disease in the United States each year.[3] The sub-group of young cardiovascular deaths that occur suddenly has a particularly profound effect on the individual's family and local community.

Screening of asymptomatic individuals for cardiac conditions, such as cardiomyopathies and channelopathies, has been proposed as a strategy to reduce the incidence of sudden cardiac death in young people. The best evidence that such a strategy might be effective comes from a single Italian before-after study that reported a decreased incidence of sudden cardiac death in athletes following the introduction of a mandatory athlete screening programme.[4] In view of the limitations of the current evidence, screening of young asymptomatic non-athletes is not presently supported by either the American Heart Association or European Society of Cardiology.[5-7]

For organisations that make decisions regarding the implementation of population screening programmes, such as the US Preventive Services Task Force and UK National Screening Committee, a clear understanding of the incidence of the target condition provides important context for decision-making.[8 9] In particular, incidence is an indicator of the size of the health problem, and maximum benefit which could be gained from screening, to be balanced against the potential harms such as stopping young people from participating in exercise. Previous reviews of sudden cardiac death incidence have focussed on the incidence across all ranges, or in specific populations.[10 11] The aim of this systematic review is to describe current evidence on the incidence of sudden cardiac death in the young.

Methods

We conducted a systematic review of studies that report the incidence of sudden cardiac death in the young. We were originally commissioned by the UK National Screening Committee to undertake a rapid review of incidence studies.[12] We subsequently chose to develop the rapid review in to this systematic review, with no limitation on study location, date, or publication language.

The review is written in accordance with the Preferred Reporting Items for Systematic Reviews and Meta-Analyses (PRISMA) guidelines. The review is registered with PROSPERO (CRD42019120563).[13]

Search and study selection

We searched MEDLINE (1946-current), EMBASE (1947-current) and the Cochrane library (inception-current) for eligible studies. The search strategy was developed by an information scientist. We, used a combination of keywords and MESH terms to describe the population (e.g. young adult, adolescent), condition (e.g. sudden death, sudden cardiac death), and study type (e.g. cohort, longitudinal) of interest. An example search strategy is included in the electronic supplement.

Following searches and duplicate removal, two reviewers independently screened study titles and abstracts. Conflicts were resolved through discussion or, where needed, arbitration by a third reviewer. The same process was adopted for review of full-text papers. We identified additional studies through forward and backward citation tracking of included studies.

For peer review only - http://bmjopen.bmj.com/site/about/guidelines.xhtml

Study eligibility criteria

We included studies that described the incidence (per 100,000 person-years) of sudden cardiac death in the young, or that provided sufficient data to allow calculation of the incidence. We defined a young individual as someone aged 12-39 years. This age range reflects the target group for a sudden cardiac death programme that was recently considered by the UK National Screening Committee.[12] The lower age cut-off of 12 years was used in the Italian sudden cardiac death screening study.[4] For reasons of pragmatism, we included studies where the reported incidence combined individuals in our target age range with younger individuals (≥ one-year). Exclusion of individuals under one-year avoided the risk of conflating sudden cardiac death with sudden infant death syndrome. We excluded studies where the reported incidence combined individuals in our target age range with older individuals (≥ 40-years), due to the marked increase in sudden cardiac death incidence from the age of 40.[14]

We also excluded studies that reported incidence only in a population with previously known disease, or only included deaths that occurred at certain times of day (e.g. at work or school) or during specific activities (e.g. sports). We imposed no restriction on language or publication date. We did not mandate a specific definition of sudden cardiac death or case-ascertainment process, but these were recorded during data extraction and considered in risk of bias assessments.

Our main population of interest was the general population aged 12-39. To be included in the main analysis, studies were required to report incidence in a general population that included males and females with an age group that spanned at least ten-years between 12 and 39 years. Where studies reported more than one age sub-group (e.g. 12-21 years; 22-31 years), we selected the sub-group closest to the age of mid-late teens to be included in the main analysis. Our planned sub-groups included incidence by age, sex, race, and athletic status (including military personnel). We do not report sub-groups of sub-groups (for example, athletes broken down by sex).

Data extraction and analysis

We used a piloted pro forma to extract population characteristics, study design case identification methods, use of systematic screening, and outcome data. We assessed risk of bias using the Joanna Briggs Institute critical appraisal checklist for prevalence studies.[15] We developed review-specific criteria for each checklist question. Two reviewers independently extracted data and assessed risk of bias, with discrepancies resolved through discussion. Papers published in a language other than English were translated by a fluent speaker.

We extracted incidence per 100,000 person-years and associated 95% confidence interval from the papers. Where these data were not reported, we calculated them from the reported number of sudden cardiac deaths and person follow-up years. We computed the 95% confidence interval based on a binomial distribution, using Stata Version 15.1. Clinical heterogeneity in relation to population characteristics and case ascertainment methods precluded pooling of data.

Patient/ public involvement

We did not involve patients or members of the public in setting the research question, designing the study, undertaking the study, the interpretation of the results, or study write-up.

Results

For peer review only - http://bmjopen.bmj.com/site/about/guidelines.xhtml

We performed initial database searches on 29th April 2019, and updated searches on 16th November 2019. Through searches and citation tracking, we identified 8360 unique citations of which we reviewed the full-text of 238. We excluded 27 of the 65 papers initially included due to substantial or complete data overlap with other studies (figure one).

Most of the 38 included studies[4 16-52] were undertaken in Europe (n=20, 53%) and North America (n=14, 37%) (table one). The majority were retrospective studies (n=29, 76%). Median number of deaths and person follow-up years were 64 (interquartile range 40 to 251) and 6.97 million (interquartile range 2.34 million to 23.70 million) respectively. Study duration ranged from one-year to 41-years.

There was variability in the population, case ascertainment methods, and sudden cardiac death definition used between studies. This methodological heterogeneity precluded meta-analysis. Only nine studies (24%) stated that they used the AHA/ESC definition of sudden cardiac death. Our risk of bias assessment found that most studies were at high risk of bias. (figure two; electronic supplement table S1) As shown in figure two, the main factor contributing to a high risk of bias was the method used to identify cases of sudden cardiac death. In five (13%) studies, sudden cardiac death was identified using death certificates. The remaining studies used a range of approaches that typically incorporated autopsy data to some extent.

Our main analysis included 26 studies (figure three). The median of reported incidence was 1.7 sudden cardiac death per 100,000 person-years (interquartile range (IQR) 1.3 to 2.6, range 0.75 to 11.9). Of the 26 studies, 17 (65%) reported an incidence of less than two sudden cardiac death events per 100,000 person-years, and 14 (54%) studies reported an incidence between one and two cases per 100,000 person-years. All four studies reporting an incidence over three cases per 100,000 person-years were at high risk of bias across multiple domains.

Geographically, incidence appeared highest in African and Asian countries, although this may in part reflect the case ascertainment process and associated risk of bias. These studies also tended to include only older individuals (≥ 18-years).

For our sub-group analysis, sex, age, athletic status, and race were reported in 13, 11, 9, and 1 studies respectively. We observed a consistently higher incidence in males (figure four). In males, median incidence was 2.7 sudden cardiac death per 100,000 person-years (IQR 1.8 to 4.4, range 1.3 to 36), compared with a median incidence in females of 0.9 cases per 100,000 person-years (IQR 0.6 to 1.4, range 0 to 7.0).

The use of different age cut-offs within studies made comparison between studies on the association of incidence of sudden cardiac death with age challenging (electronic supplement figure S1).

Incidence in military personnel was reported in three studies and appeared to be consistently higher than in other populations (electronic supplement figure S2). All three studies were undertaken in American military personnel and reported that individuals received pre-enrolment screening for cardiac conditions. We did not observe a clear difference in incidence between athletic and non-athletic populations. The highest incidence was reported in a prospective study of screened UK soccer players, although the low number of sudden cardiac deaths (8 events) means that the estimate of the incidence is imprecise.

Race was reported in a single American study which broke incidence down in to four categories (African American, White, Hispanic, other). Incidence was highest in the African-American population (electronic supplement figure S3).

For peer review only - http://bmjopen.bmj.com/site/about/guidelines.xhtml

Discussion

In this systematic review, we included 38 studies from five continents that reported incidence of sudden cardiac death in young individuals. We identified significant variability in the reported incidence across studies. In our main analysis, most studies reported an incidence of between one and two sudden cardiac deaths per 100,000 person-years, although reported incidence ranged from 0.75 to 11.9 cases per 100,000 person-years. This variability may reflect differences in population, case-ascertainment methods, and definition used for sudden cardiac death. In pre-defined subgroups, we observed that male sex and increasing age seem to be associated with increased incidence.

Strengths and weaknesses of the study

The key strength of this review was the systematic identification and synthesis of 38 studies from a wide range of settings. We did not restrict inclusion by date and language, with fluent speakers used for translation.

Our review has four key limitations. Firstly, whilst the geographical area covered by index studies was often large with a long recruitment period, the median number of sudden cardiac deaths identified per study was 64 cases. The method used to identify sudden cardiac death was often sub-optimal, such that misclassification bias may have significantly impacted the reported incidence in some studies.[53] Secondly, heterogeneity between studies in terms of the population and case ascertainment method used precluded meta-analysis. Thirdly, particularly for sub-groups, studies often described only the absolute incidence, and did not provide sufficient additional data to calculate the 95% confidence interval. As such, we could not describe the precision of the incidence rate in many studies. Finally, we limited our condition of interest to sudden cardiac death, and did not examine incidence of sudden cardiac arrest. Whilst survival after sudden cardiac arrest is associated with important morbidity, the standard Utstein categorisation of cardiac arrest cause encompasses all medical causes, of which cardiac causes are only a sub-set.[54 55]

Comparison with other studies

Our review builds on two previous systematic reviews that described sudden cardiac death incidence across all age groups or in specific groups of young individuals.[10 11] In line with our review findings, these reviews reported issues across index studies in relation to variability in reported incidence, use of different definitions of sudden cardiac death, and use of different methods to identify cases of sudden cardiac death.

Variability in reported incidence reflects in part the challenge of accurately identifying cases of sudden cardiac death. The method used to identify deaths varied across studies, although most used administrative data sources. Nevertheless, the inclusion of some patient groups, such as in-hospital deaths in patients admitted for a minor procedure, may have varied across studies. The definition of sudden cardiac death is based on both the circumstances and cause of death, such that classification requires knowledge of the cause of death and events leading up to the death.[5 6] Only nine studies used the ESC/AHA definition of sudden cardiac death. Other studies either predated this definition of sudden cardiac death or adopted a different definition to fit the method used to identify cases of sudden cardiac death. Death certificate data provide an efficient way to estimate sudden cardiac

For peer review only - http://bmjopen.bmj.com/site/about/guidelines.xhtml
For peer review only

death incidence. However, their use typically precludes the use of the AHA/ESC definition of sudden cardiac death as the certificate does not include information on the circumstances of the death. As such, studies relying on death certificate data likely over-estimate sudden cardiac death incidence.[19]

Determination of the cause of sudden cardiac death usually requires an autopsy, ideally by a specialist pathologist.[56 57] In included studies, reporting of the proportion of patients that received an autopsy and type of autopsy performed was often unclear. This is reflected in our risk of bias assessments. The decision to undertake an autopsy in a specific case may be influenced by factors such as the legal requirement for an autopsy, family acceptance of autopsy, and the identification of a likely cause of death without autopsy. A recent European study reported an autopsy rate in young sudden death of less than 50%.[45] Where autopsy is not used, cause of death may be misclassified, although the impact of this misclassification on overall incidence is uncertain. In some cases, cause of death may remain uncertain even after autopsy. In these cases, genetic testing of the deceased may be informative in determining both the cause of death and the need for cardiac screening of the victim's family.[17 57 58] In an Australasian study, genetic testing of 113 cases of unexplained sudden cardiac death cases identified cardiac gene variants that were definitely or probably pathogenic in over a quarter of cases.[17]

Studies included in this review spanned a 66-year period (1950 to 2016), although most studies report data collected since 2000. Recent observational studies provide evidence of a decreasing incidence of sudden cardiac death.[25 42 59] A Danish study reported a 3% average annual decrease in sudden cardiac death incidence between 2000 and 2009.[59] Potential explanations for this decrease include improved investigation of inherited cardiac disease, public health strategies to deal with obesity and smoking, and improved response to out-of-hospital cardiac arrest..

Clinical and policy implications

The decision to implement screening programmes in both the UK and USA requires evidence that the benefits of screening outweigh its harms.[9 60] Disease incidence data provides important context for evaluating evidence, particularly in the absence of direct evidence of benefit from randomised controlled trials. In less common conditions, a key concern are the potential harms that may result from false-positive results, and overdiagnosis of disease which may never become symptomatic. Nevertheless, rare disease may still meet criteria for population-based screening conditions, such as maple syrup urine disease in newborns.[61]

The incidence of a condition describes the number of individuals that might benefit from screening. In practice, however, not all young individuals that sustain sudden cardiac death have the potential to benefit from screening.

Firstly, to be effective, screening must reliably identify individuals with disease. In this context, screening must detect a range of structural and electrical cardiac disease. The optimal screening strategy remains uncertain. A wide range of strategies have been described, ranging from those that comprise only a physical examination or medical history to more detailed assessments that include a 12-lead electrocardiogram.[12 62] In programmes where a 12-lead electrocardiogram is collected, a number of assessment criteria have been developed to determine the need for follow-up testing.[63-65] In a prospective cohort study of 11,168 screened teenage footballers in which screening incorporated a physical examination, health questionnaire, echocardiogram, and 12-lead electrocardiogram, six out of eight individuals who subsequently experienced sudden cardiac death did not have disease detected at screening.[23] This highlights a key challenge for policy makers in that, even if they decide to support a screening programme, detailed consideration will need to be

For peer review only - http://bmjopen.bmj.com/site/about/guidelines.xhtml

given to the screening process and both the financial implications and individual harm that may stem from false-negatives and false-positive screening results.[66 67]

Secondly, population-level screening is targeted at asymptomatic individuals without a family history of sudden cardiac death. The incidence of sudden cardiac death reported in index studies included both asymptomatic low-risk individuals and individuals at increased risk of sudden cardiac death who, in some countries, may already have access to screening.[6 68-73] A recent study suggested that around one-third of young individuals experienced warning symptoms in the month preceding their sudden cardiac death, indicating the need for clinicians to consider cardiological investigations in symptomatic young individuals.[74] Examples of individuals at increased risk of sudden cardiac death include those with diagnosed cardiac disease, asymptomatic individuals with a family history of sudden cardiac death, and symptomatic individuals without diagnosed cardiac disease. As such, policy makers need to be cognisant that incidence reported in population-based studies will over-estimate the potential benefit of screening programmes which are targeted at low-risk asymptomatic individuals.

Finally, effective screening relies on the individuals willingness to be screened. To date, most studies report screening which, whilst technically voluntary, was mandated for participation in an activity, such as sport. Studies of athletes report young people are supportive of screening.[75 76] In practice, however, uptake of school-based cardiac screening programmes varies from 56% to 79%, with substantial variation by year and school.[77 78]

In conclusion, studies of incidence of sudden cardiac death in young individuals have produced varying estimates of the incidence, although most studies report the incidence to be between one and two cases per 100,000 person-years. In these studies of young individuals, incidence was highest in males and amongst older individuals.

For peer review only

For peer review only - http://bmjopen.bmj.com/site/about/guidelines.xhtml

Table and figure list

Figure one: PRISMA flow chart of study selections

Figure two: Risk of bias assessment

Figure three: Incidence of sudden cardiac death in general population

Figure footnote: Range following study name indicates population age range

Figure four: Incidence of sudden cardiac death in general population- sex sub-groups

Table one: summary of included studies

Author contributions

KC accepts full responsibility for the work and conduct of the study, had access to the data, and controlled the decision to publish.

KC designed the study, acted as reviewer, undertook the analysis, and drafted the manuscript. OP and RF acted as reviewers. PR designed database searches. KP, WB, AC, GDP, JY and STP contributed to study design. All authors contributed to the write up and approved the final version.

Ethics approval

Not required

Data sharing statement

All data relevant to the study are included in the article or uploaded as supplementary information

Funding

This work was supported by Public Health England (Award/Grant number is not applicable). Public Health England originally commissioned a rapid review through the UK National Screening Committee, on which this systematic review was based. Otherwise, Public Health England had no role in study design; collection, analysis, and interpretation of data; in the writing of the report; and in the decision to submit the article for publication. All authors, external and internal, had full access to all of the data (including statistical reports and tables) in the study and can take responsibility for the integrity of the data and the accuracy of the data analysis.

This work was supported by the National Institute for Health Research (NIHR) Applied Research Collaboration (ARC) West Midlands (Award/Grant number is not applicable).

Professor Perkins is supported as an NIHR senior investigator. Professor Taylor-Phillips is supported by an NIHR Career Development Fellowship (CDF-2016-09-018).

The opinions are those of the authors and not the NIHR, the UK National Screening Committee, the NHS or the Department of Health and Social Care.

For peer review only - http://bmjopen.bmj.com/site/about/guidelines.xhtml

Competing interests

All authors have completed the ICMJE uniform disclosure at www.icmje.org/coi_disclosure.pdf and declare: KC, KP, AC, PR, GDP, JY, STP received financial support from Public Health England for the rapid review on which this review was based; no financial relationships with any organisations that might have an interest in the submitted work in the previous three years; KC, JY and GDP have volunteer roles with the International Liaison Committee on Resuscitation, European Resuscitation Council and Resuscitation Council UK.

For peer review only

For peer review only - http://bmjopen.bmj.com/site/about/guidelines.xhtml

References

1. World Health Organisation. World Health Statistics 2018: Monitoing health for the SDGs (sustainable development goals). Geneva, 2018. [Available from http://apps.who.int/iris/bitstream/handle/10665/272596/9789241565585-eng.pdf?ua=1 accessed 13/02/2020]

2. Center for Disease Control and Prevention. 10 leading causes of death by age group, United States- 2017. [Available from: https://www.cdc.gov/injury/wisqars/LeadingCauses.html accessed 20/12/2019]

3. Benjamin EJ, Muntner P, Alonso A, et al. Heart Disease and Stroke Statistics- 2019 Update: A Report From the American Heart Association. Circulation 2019;139(10):e56-e528.

4. Corrado D, Basso C, Pavei A, et al. Trends in Sudden Cardiovascular Death in Young Competitive Athletes After Implementation of a Preparticipation Screening Program. JAMA 2006;296(13):1593-601.

5. Al-Khatib SM, Stevenson WG, Ackerman MJ, et al. 2017 AHA/ACC/HRS Guideline for Management of Patients With Ventricular Arrhythmias and the Prevention of Sudden Cardiac Death. Circulation 2018;138(13):e272-e391.

6. Priori SG, Blomström-Lundqvist C, Mazzanti A, et al. 2015 ESC Guidelines for the management of patients with ventricular arrhythmias and the prevention of sudden cardiac death. Eur Heart J 2015;36(41):2793-867.

7. Van Brabandt H, Desomer A, Gerkens S, et al. Harms and benefits of screening young people to prevent sudden cardiac death. BMJ 2016;353:i1156.

8. US Preventive Services Task Force. Procedure Manual 2018 [Available from: https://www.uspreventiveservicestaskforce.org/Page/Name/procedure-manual accessed 08/01/2020]

9. Public Health England. Criteria for appraising the viability, effectiveness and appropriateness of a screening programme. [Available from: https://www.gov.uk/government/publications/evidence-review-criteria-national-screening-programmes/criteria-for-appraising-the-viability-effectiveness-and-appropriateness-of-a-screening-programme accessed 13/02/2020]

10. Harmon KG, Drezner JA, Wilson MG, et al. Incidence of sudden cardiac death in athletes: a state-of-the-art review. Heart 2014;100(16):1227-34.

11. Kong MH, Fonarow GC, Peterson ED, et al. Systematic Review of the Incidence of Sudden Cardiac Death in the United States. JACC 2011;57(7):794-801.

12. Couper K, Poole K, Bradlow W, et al. Sudden cardiac death in the young: External review against programme appraisal criteria for the UK National Screening Committee. [Available from https://legacyscreening.phe.org.uk/policydb_download.php?doc=1239 accessed 17/08/2020]

13. Moher D, Liberati A, Tetzlaff J, et al. Preferred reporting items for systematic reviews and meta-analyses: the PRISMA statement. Ann Intern Med 2009;151(4):264-9.

For peer review only

For peer review only - http://bmjopen.bmj.com/site/about/guidelines.xhtml

14. Risgaard B, Winkel BG, Jabbari R, et al. Burden of sudden cardiac death in persons aged 1 to 49 years: nationwide study in Denmark. Circ Arrhythm Electrophysiol 2014;7(2):205-11.

15. Joanna Briggs Institute. Joanna Briggs Institute Checklist for Prevalence Studies 2017 [Available from: http://joannabriggs.org/assets/docs/critical-appraisal-tools/JBI_Critical_Appraisal-Checklist_for_Prevalence_Studies2017.pdf accessed 17/03/2019].

16. Anastasakis A, Papatheodorou E, Ritsatos K, et al. Sudden unexplained death in the young: epidemiology, aetiology and value of the clinically guided genetic screening. EP Europace 2018;20(3):472-80.

17. Bagnall RD, Weintraub RG, Ingles J, et al. A Prospective Study of Sudden Cardiac Death among Children and Young Adults. NEJM 2016;374(25):2441-52.

18. Bonny A, Tibazarwa K, Mbouh S, et al. Epidemiology of sudden cardiac death in Cameroon: The first population-based cohort survey in sub-Saharan Africa. Int J Epidemiol 2017;46(4):1230-38.

19. Chugh SS, Jui J, Gunson K, et al. Current burden of sudden cardiac death: Multiple source surveillance versus retrospective death certificate-based review in a large U.S. community. JACC 2004;44(6):1268-75.

20. Eckart RE, Shry EA, Burke AP, et al. Sudden death in young adults: An autopsy-based series of a population undergoing active surveillance. JACC 2011;58(12):1254-61.

21. Fragkouli K, Vougiouklakis T. Sudden cardiac death: An 11-year postmortem analysis in the region of Epirus, Greece. Pathol Res Pract 2010;206(10):690-94.

22. Hofer F, Fellmann F, Schlapfer J, et al. Sudden cardiac death in the young (5-39 years) in the canton of Vaud, Switzerland. BMC Cardiovasc Disord 2014;14:140.

23. Malhotra A, Dhutia H, Finocchiaro G, et al. Outcomes of Cardiac Screening in Adolescent Soccer Players. NEJM 2018;379(6):524-34.

24. Papadakis M, Sharma S, Cox S, et al. The magnitude of sudden cardiac death in the young: a death certificate-based review in England and Wales. EP Europace 2009;11(10):1353-8.

25. Wisten A, Krantz P, Stattin EL. Sudden cardiac death among the young in Sweden from 2000 to 2010: an autopsy-based study. EP Europace 2017;19(8):1327-34.

26. Winkel BG, Risgaard B, Bjune T, et al. Gender differences in sudden cardiac death in the young-a nationwide study. BMC Cardiovasc Disord 2017;17:19.

27. Pilmer CM, Kirsh JA, Hildebrandt D, et al. Sudden cardiac death in children and adolescents between 1 and 19 years of age. Heart Rhythm 2014;11(2):239-45.

28. Pilmer CM, Porter B, Kirsh JA, et al. Scope and nature of sudden cardiac death before age 40 in Ontario: a report from the cardiac death advisory committee of the office of the chief coroner. Heart Rhythm 2013;10(4):517-23.

29. Harmon KG, Asif IM, Maleszewski JJ, et al. Incidence, Cause, and Comparative Frequency of Sudden Cardiac Death in National Collegiate Athletic Association Athletes: A Decade in Review. Circulation 2015;132(1):10-9.

30. Harmon KG, Asif IM, Maleszewski JJ, et al. Incidence and Etiology of Sudden Cardiac Arrest and Death in High School Athletes in the United States. Mayo Clin Proc 2016;91(11):1493-502.

For peer review only - http://bmjopen.bmj.com/site/about/guidelines.xhtml

31. Maron BJ, Haas TS, Murphy CJ, et al. Incidence and causes of sudden death in U.S. college athletes. JACC 2014;63(16):1636-43.

32. Maron BJ, Haas TS, Duncanson ER, et al. Comparison of the Frequency of Sudden Cardiovascular Deaths in Young Competitive Athletes Versus Nonathletes: Should We Really Screen Only Athletes? Am J Cardiol 2016;117(8):1339-41.

33. Morentin B, Audicana C. Population-based study of out-of-hospital sudden cardiovascular death: incidence and causes of death in middle-aged adults. Rev Esp Cardiol 2011;64(1):28-34.

34. Zhang J, Zhou X, Xing Q, et al. Epidemiological investigation of sudden cardiac death in multiethnic Xinjiang Uyghur autonomous region in Northwest China. BMC Public Health 2019;19(1):116.

35. Wren C, O'Sullivan JJ, Wright C. Sudden death in children and adolescents. Heart 2000;83(4):410-3.

36. Wisten A, Forsberg H, Krantz P, et al. Sudden cardiac death in 15-35-year olds in Sweden during 1992-99. J Intern Med 2002;252(6):529-36.

37. Vaartjes I, Hendrix A, Hertogh EM, et al. Sudden death in persons younger than 40 years of age: incidence and causes. Eur J Cardiovasc Prev Rehabil 2009;16(5):592-6.

38. Uuskula M, Lamp K, Vali M. An age-related difference in the ratio of sudden coronary death over acute myocardial infarction in Estonian males. J Clin Epidemiol 1998;51(7):577-80.

39. Morentin B, Paz Suarez-Mier M, Audicana C, et al. Incidence and causes of sudden death in persons less than 36 years of age. Med Clin 2001;116(8):281-5.

40. Margey R, Roy A, Tobin S, et al. Sudden cardiac death in 14- to 35-year olds in Ireland from 2005 to 2007: a retrospective registry. EP Europace 2011;13(10):1411-8.

41. Goudevenos JA, Papadimitriou ED, Papathanasiou A, et al. Incidence and other epidemiological characteristics of sudden cardiac death in northwest Greece. Int J Cardiol 1995;49(1):67-75.

42. El-Assaad I, Al-Kindi SG, Aziz PF. Trends of Out-of-Hospital Sudden Cardiac Death Among Children and Young Adults. Pediatrics 2017;140(6):e20171438.

43. Eckart RE, Scoville SL, Campbell CL, et al. Sudden death in young adults: a 25-year review of autopsies in military recruits. Ann Intern Med 2004;141(11):829-34.

44. Driscoll DJ, Edwards WD. Sudden unexpected death in children and adolescents. JACC 1985;5(6):118B-21B.

45. Asatryan B, Vital C, Kellerhals C, et al. Sports-related sudden cardiac deaths in the young population of Switzerland. PLoS ONE 2017;12(3):e0174434.

46. Hua W, Zhang L-F, Wu Y-F, et al. Incidence of Sudden Cardiac Death in China: Analysis of 4 Regional Populations. JACC 2009;54(12):1110-18.

47. Einarsson G, Björnsson J, Gunnarsson G. Sudden cardiac death in the young. A 30 year nation-wide study in Iceland. Int J Cardiol 2007;119:S34-S35.

48. Neuspiel DR, Kuller LH. Sudden and Unexpected Natural Death in Childhood and Adolescence. JAMA 1985;254(10):1321-25.

For peer review only

For peer review only - http://bmjopen.bmj.com/site/about/guidelines.xhtml

49. Drehner D, Neuhauser KM, Neuhauser TS, et al. Death among U.S. Air Force basic trainees, 1956 to 1996. Mil Med 1999;164(12):841-7.

50. Karvouni E, Anastasakis A, Spiliopoulou C, et al. Sudden death in the young in Greece. Hell J Cardiol 2000;41:350-70.

51. Anderson RE, Hill RB, Broudy DW, et al. A population-based autopsy study of sudden, unexpected deaths from natural causes among persons 5 to 39 years old during a 12-year period. Hum Pathol 1994;25(12):1332-40.

52. Perez G, Marrugat J, Sunyer J, et al. Sudden cardiac death in the counties of Girona. Med Clin 1992;99(13):489-92.

53. Delgado-Rodríguez M, Llorca J. Bias. Journal Epidemiol Community Health 2004;58(8):635-41.

54. Lilja G, Nielsen N, Bro-Jeppesen J, et al. Return to Work and Participation in Society After Out-of-Hospital Cardiac Arrest. Circ Cardiovasc Qual Outcomes 2018;11(1):e003566.

55. Perkins GD, Jacobs IG, Nadkarni VM, et al. Cardiac arrest and cardiopulmonary resuscitation outcome reports: update of the Utstein Resuscitation Registry Templates for Out-of-Hospital Cardiac Arrest: a statement for healthcare professionals from a task force of the International Liaison Committee on Resuscitation and the American Heart Association Emergency Cardiovascular Care Committee and the Council on Cardiopulmonary, Critical Care, Perioperative and Resuscitation. Circulation 2015;132(13):1286-300.

56. de Noronha SV, Behr ER, Papadakis M, et al. The importance of specialist cardiac histopathological examination in the investigation of young sudden cardiac deaths. EP Europace 2013;16(6):899-907.

57. Basso C, Aguilera B, Banner J, et al. Guidelines for autopsy investigation of sudden cardiac death: 2017 update from the Association for European Cardiovascular Pathology. Virchows Archiv 2017;471(6):691-705.

58. Lahrouchi N, Raju H, Lodder EM, et al. Utility of Post-Mortem Genetic Testing in Cases of Sudden Arrhythmic Death Syndrome. JACC 2017;69(17):2134-45.

59. Lynge TH, Nielsen JL, Blanche P, et al. Decline in incidence of sudden cardiac death in the young: a 10-year nationwide study of 8756 deaths in Denmark. EP Europace 2019;21(6):909-17.

60. U.S. Preventive Services Task Force. Grade Definitions 2012 [Available from: https://www.uspreventiveservicestaskforce.org/Page/Name/grade-definitions accessed 06/01/2019]

61. UK National Screening Committee. The UK NSC recommendation on Maple Syrup Urine Disease 2014 [Available from: https://legacyscreening.phe.org.uk/msud accessed 21st January 2020]

62. Harmon KG, Zigman M, Drezner JA. The effectiveness of screening history, physical exam, and ECG to detect potentially lethal cardiac disorders in athletes: a systematic review/meta-analysis. J Electrocardiol 2015;48(3):329-38.

63. Riding NR, Sheikh N, Adamuz C, et al. Comparison of three current sets of electrocardiographic interpretation criteria for use in screening athletes. Heart 2015;101(5):384-90.

For peer review only

64. Brosnan M, La Gerche A, Kalman J, et al. The Seattle Criteria increase the specificity of preparticipation ECG screening among elite athletes. Br J Sports Med 2014;48(15):1144-50.

65. Dunn TP, Pickham D, Aggarwal S, et al. Limitations of Current AHA Guidelines and Proposal of New Guidelines for the Preparticipation Examination of Athletes. Clin J Sport Med 2015;25(6):472-77.

66. Dhutia H, Malhotra A, Yeo TJ, et al. Inter-Rater Reliability and Downstream Financial Implications of Electrocardiography Screening in Young Athletes. Circ Cardiovasc Qual Outcomes 2017;10(8):e003306.

67. Dhutia H, Malhotra A, Gabus V, et al. Cost Implications of Using Different ECG Criteria for Screening Young Athletes in the United Kingdom. J Am Coll Cardiol 2016;68(7):702-11.

68. Department of Health. National Service Framework for Coronary Heart Disease- Chapter Eight: Arrhythmias and Sudden Cardiac Death London: Department of Health, 2005. [Available from: https://webarchive.nationalarchives.gov.uk/20100420225444/http://www.dh.gov.uk/en/Healthcare/Longtermconditions/Vascular/Coronaryheartdisease/DH_4117048 accessed 13/02/2020]

69. National Institute for Health and Care Excellence. Clinical Guideline 109: Transient loss of consciousness ('blackouts') in over 16s 2014 [Available from: https://www.nice.org.uk/guidance/cg109 accessed 18/03/2019]

70. Brignole M, Moya A, de Lange FJ, et al. 2018 ESC Guidelines for the diagnosis and management of syncope. Eur Heart J 2018;39(21):1883-948.

71. Norrish G, Field E, Mcleod K, et al. Clinical presentation and survival of childhood hypertrophic cardiomyopathy: a retrospective study in United Kingdom. Eur Heart J 2018;40(12):986-93.

72. Alexander PMA, Nugent AW, Daubeney PEF, et al. Long-Term Outcomes of Hypertrophic Cardiomyopathy Diagnosed During Childhood. Circulation 2018;138(1):29-36.

73. Ranthe MF, Winkel BG, Andersen EW, et al. Risk of cardiovascular disease in family members of young sudden cardiac death victims. Eur Heart J 2013;34(7):503-11.

74. Jayaraman R, Reinier K, Nair S, et al. Risk Factors of Sudden Cardiac Death in the Young. Circulation 2018;137(15):1561-70.

75. Asif IM, Annett S, Ewing JA, et al. Psychological impact of electrocardiogram screening in National Collegiate Athletic Association athletes. Br J Sports Med 2017;51(20):1489-92.

76. Asif IM, Johnson S, Schmieg J, et al. The psychological impact of cardiovascular screening: the athlete's perspective. Br J Sports Med 2014;48(15):1162-6.

77. Marek J, Bufalino V, Davis J, et al. Feasibility and findings of large-scale electrocardiographic screening in young adults: data from 32,561 subjects. Heart Rhythm 2011;8(10):1555-9.

78. Vilardell P, Brugada J, Aboal J, et al. Characterization of electrocardiographic findings in young students. Rev Esp Cardiol 2020;73(2):139-44.

For peer review only - http://bmjopen.bmj.com/site/about/guidelines.xhtml

Table one:

	Design	Setting	Population: Type/ Age	SCD cases	Follow-up years	Systematic screening	SCD definition	Case ascertainment	
								Identification of deaths	Main method to determine SCD
Anastasakis 2018	PC	Greece 2002-2010	General 1-35	226	12,750,000	No	AHA/ ESC	Government records/ death certificates	Autopsy record review
Anderson 1994	RC	USA 1977-1988	General 5-39	183	9,836,196	No	Other	Medical examiner records	Autopsy record review
Asatryan 2017	RC	Switzerland 1999-2010	General 10-39	349[†]	25,344,456	Athletes only	Other	Forensic medicine database	Autopsy record review
Bagnall 2016	PC	Austrasia 2010-2012	General 1-35	490	37,770,000	No	AHA/ ESC	Government records/ death certificates; Autopsy records	Autopsy record review
Bonny 2017	PC	Cameroon 2013	General 18-39	-	-	No	Other	Government records/ death certificates	Medical record review; interviews
Chugh 2004	PC	USA 2002-2003	General 10-34	-	-	No	AHA/ ESC	Hospital/ ambulance/ medical examiner records; death certificates	Autopsy/ medical record review; ICD code
Corrado 2006	PC	Italy 1979-2004	General 12-35	320	36,144,100	Athletes only	Other	Medical centre registers, media searches (prospective registry)	Autopsy record review
Drehner 1999	RC	USA 1956-1996	Military 17-26	23	340,990	Yes	Not stated	Military records	Autopsy record review
Driscoll 1985	RC	USA 1950-1982	General 1-22	7	930,678	No	Other	Government records/ death certificates	Autopsy/ medical record review
Eckart 2004	RC	USA 1977-2001	Milirary 17-35	64	-	Yes	Other	Military records	Autopsy/ military record review
Eckart 2011	RC	USA 1998-2008	Military <20	-	-	Yes	Other	Military records	Autopsy record review
Einarsson 2007	RC	Iceland 1974-2004	General 12-35	42	-	No	Not stated	Government records/ death certificates	Autopsy/ medical record review
El-Assaad 2017	RC	USA 1999-2015	General 1-34	31492	-	No	Other	Government records/ death certificates	ICD code
Fragkouli 2010	RC	Greece 1998-2008	General 1-35	28	-	No	Other	Forensic laboratory records	Autopsy/ medical/ police record review
Goudevenos 1995	PC	Greece 1990-1993	General 30-39	-	-	No	Other	Hospital/ coroner records; Department of statistics	Autopsy/ medical record review; interviews
Harmon 2015	RC	USA 2003-2013	Athletes 17-24	79	4,242,519	Yes	AHA/ ESC	Media search; NCAA records	Autopsy record review; interviews
Harmon 2016	RC	USA 2007-2013	Athletes 14-18	69	6,974,640	Yes	Other	Media search	Autopsy record review
Hofer 2014	RC	Switzerland 2000-2007	General 5-39	40	2,340,368	No	Not stated	Government records/ death certificates	ICD code
Hua 2009	PC	China 2005-2006	General 25-34	9	115,188	No	Other	Government records/ death certificates	Medical record review; interviews

For peer review only

For peer review only - http://bmjopen.bmj.com/site/about/guidelines.xhtml

Karvouni 2000	RC	Greece 1997-1999	General 1-35	33‡	4,033,333	No	Other	Forensic laboratory, hospital and ambulance records	Autopsy record review
Malhotra 2018	PC	UK 1996-2016	Athletes 15-17	8	118,351	Yes	Not stated	Voluntary reports/ survey; media search	Autopsy record review
Margey 2011	RC	Ireland 2005-2007	General 15-34	116	4,065,513	No	AHA/ ESC	Government records/ death certificates	Autopsy record review
Maron 2014	RC	USA 2002-2011	Athletes 17-26	64	4,052,369	No	Not stated	Media search; NCAA records	Autopsy record review
Maron 2016	RC	USA 2000-2014	General 14-23	27	1,308,730	No	Not stated	Medical examiner records	Autopsy record review
Morentin 2001	RC	Spain 1991-1998	General 1-35	46	4,413,088	No	Other	Forensic laboratory records	Autopsy record review
Morentin 2011	RC	Spain 2003-2008	General 35-39	42	-	No	AHA/ ESC	Government records/ death certificates	Autopsy record review
Neuspiel 1985	RC	USA 1972-1980	General 1-21	51	-	No	Other	Government records/ death certificates	Autopsy/ medical record review
Papadakis 2009	RC	UK 2002-2005	General 1-34	1677	94,256,200	No	Other	Government records/ death certificates	ICD code
Perez 1992	PC	Spain 1987-1988	General 25-34	1	-	No	Not stated	Government records/ death certificates	Medical record review; interviews
Pilmer 2013	RC	Canada 2008	General 2-29	56	-	No	Other	Coroner records	Autopsy/ medical/ police record review
Pilmer 2014	RC	Canada 2005-2009	General 1-19	116	14,893,860	No	Other	Coroner records	Autopsy record review
Uuskula 1998	RC	Estonia 1980-1996	General 20-39	251	902,241	No	Other	Government records/ death certificates	Autopsy record review
Vaartjes 2009	RC	Holland 1996-2006	General 1-39	1500	92,374,043	No	Other	Government records/ death certificates	ICD code
Winkel 2017	RC	Denmark 2000-2009	General 1-35	635	23,700,000	No	AHA/ ESC	Government records/ death certificates	Autopsy record review
Wisten 2002	RC	Sweden 1992-1999	General 15-35	181	19,514,080	No	AHA/ ESC	Government records/ national forensic medicine database	Autopsy/ medical record review
Wisten 2017	RC	Sweden 2000-2010	General 1-35	552	42,900,000	Athletes only	AHA/ ESC	Government records/ national forensic medicine database	Autopsy record review
Wren 2000	RC	England 1985-1994	General 1-20	59	8,060,000	No	Other	Coroner/ hospital records	Death certificate
Zhang 2019	RC	China 2015	General 18-35	-	-	No	Other	Government records/ death certificates	Medical record review; interviews

PC- Prospective cohort; RC- Retrospective cohort. AHA/ESC- American Heart Association/ European Society of Cardiology. NCAA National Collegiate Athletic Association. SCD- Sudden cardiac death.
† Study inflated number of SCDs to account for low autopsy rate. ‡- Unable to replicate reported incidence based on study data.

For peer review only

For peer review only - http://bmjopen.bmj.com/site/about/guidelines.xhtml

For peer review only

For peer review only - http://bmjopen.bmj.com/site/about/guidelines.xhtml

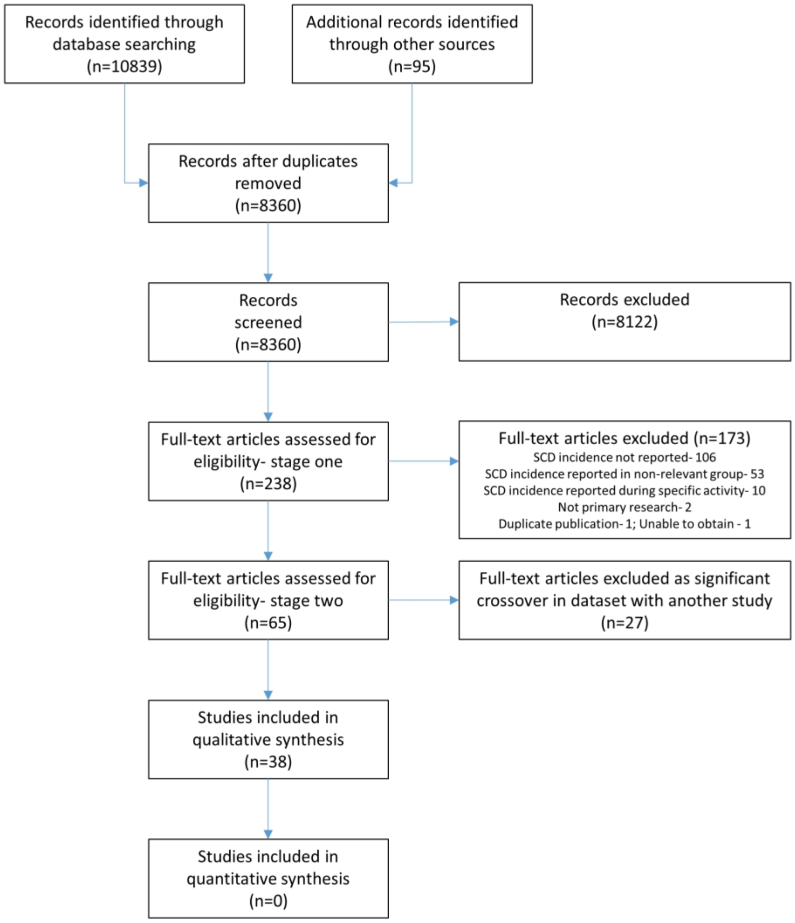

PRISMA flow chart of study selections

190x275mm (300 x 300 DPI)

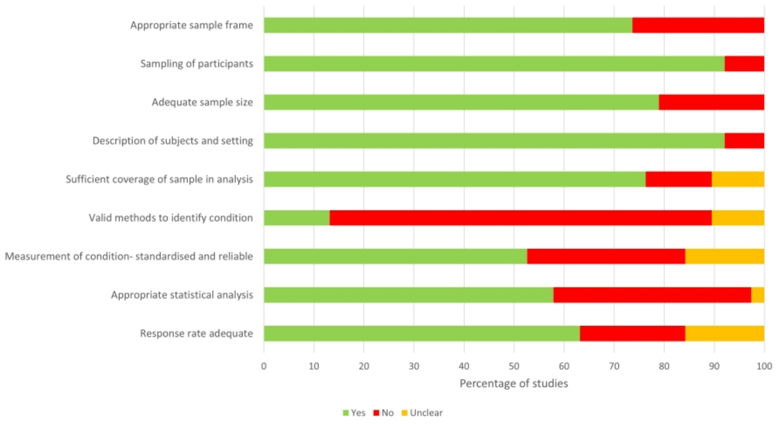

Risk of bias assessment

338x190mm (300 x 300 DPI)

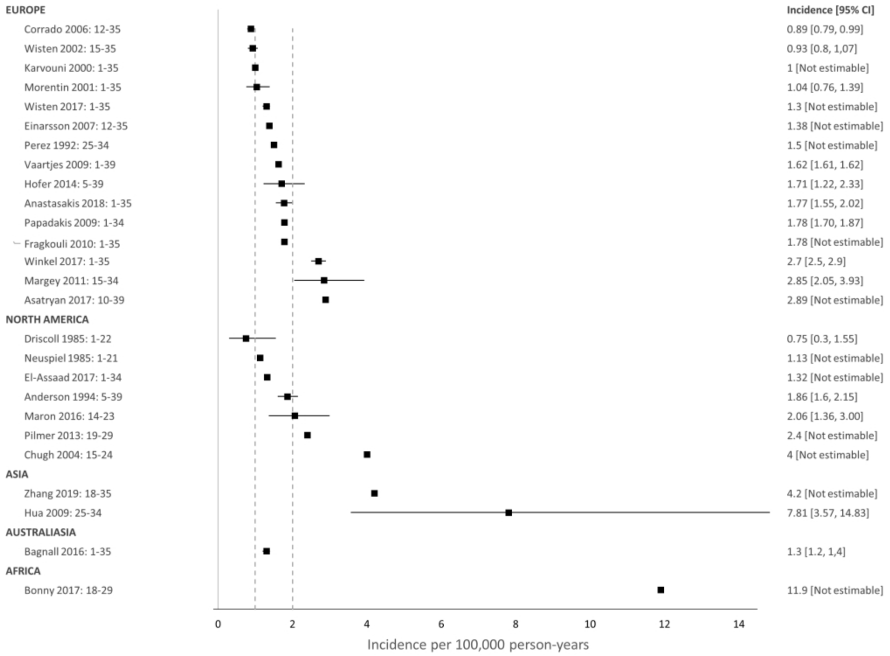

Incidence of sudden cardiac death in general population

Figure footnote: Range following study name indicates population age range

275x190mm (300 x 300 DPI)

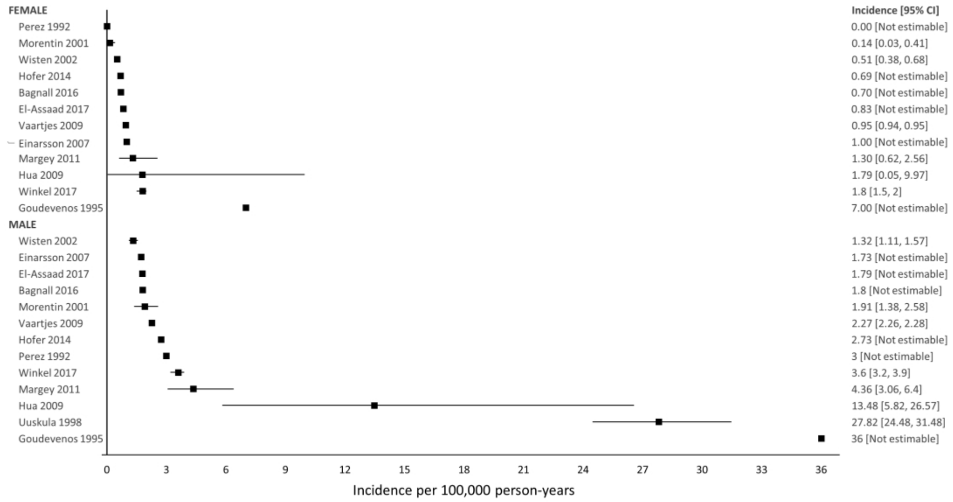

Incidence of sudden cardiac death in general population- sex sub-groups

275x190mm (300 x 300 DPI)

Incidence of sudden cardiac death in the young: a systematic review- Supplementary material

Contents:

MEDLINE search strategy- page 1

Table S1: study risk of bias assessment- page 2

Figure S1: Sudden cardiac death incidence by age- page 3

Figure S2: Sudden cardiac death incidence by athletic/ military status- page 4

Figure S3: Sudden cardiac death incidence by race- page 5

MEDLINE search strategy

1 exp Death, Sudden, Cardiac/
2 (sudden adj3 death).mp
3 cardiac death.mp
4 1 or 2 or 3
5 exp Incidence/
6 incidence.mp
7 exp Prevalence/
8 prevalence.mp
9 5 or 6 or 7 or 8
10 (adolescen* or young* or student* or athlet* or soccer or football*).mp
11 exp Young Adult/ or exp Adult/
12 exp Adolescent/
13 exp Child/
14 10 or 11 or 12 or 13
15 exp Cohort Studies/
16 exp Longitudinal Studies/
17 exp Follow-Up Studies/
18 exp Prospective Studies/
19 exp Retrospective Studies/
20 (longitudinal or prospective* or observational or registry).mp
21 (autopsy or death certificate*).mp
22 15 or 16 or 17 or 18 or 19 or 20 or 21
23 4 and 9 and 14 and 22
24 limit 23 to (case reports or comment or editorial or letter)
25 23 not 24

For peer review only

Table S1: study risk of bias assessment

	1-Appropriate sample frame	2-Sampling of participants	3-Adequate sample size	4-Description of subjects and setting	5-Sufficient coverage of sample in analysis	6-Valid methods to identify condition	7-Measurement of condition- standardised and reliable	8-Appropriate statistical analysis	9-Response rate adequate
Anastasakis 2018	Y	Y	Y	Y	Y	Y	Y	Y	Y
Anderson 1994	Y	Y	Y	Y	Y	N	Y	Y	Y
Asatryan 2017	Y	Y	Y	Y	Y	N	N	N	N
Bagnall 2016	Y	Y	Y	Y	Y	Y	Y	Y	Y
Bonny 2017	Y	N	N	N	U/C	N	Y	N	Y
Chugh 2004	Y	Y	N	Y	Y	N	N	N	Y
Corrado 2006	Y	Y	Y	Y	N	N	Y	Y	Y
Drehner 1999	N	Y	Y	Y	Y	N	U/C	N	N
Driscoll 1985	Y	Y	N	Y	Y	N	N	Y	Y
Eckart 2004	N	Y	Y	Y	Y	N	Y	Y	Y
Eckart 2011	N	Y	Y	Y	Y	N	Y	Y	N
Einarsson 2007	Y	Y	Y	Y	Y	U/C	U/C	U/C	U/C
El-Assaad 2017	Y	Y	Y	Y	Y	N	Y	N	Y
Fragkouli 2010	Y	Y	Y	Y	U/C	N	Y	N	U/C
Goudevenos 1995	N	Y	N	Y	Y	N	N	N	N
Harmon 2016	N	Y	Y	Y	N	N	N	Y	N
Harmon 2015	N	Y	Y	Y	N	N	N	Y	U/C
Hofer 2014	Y	Y	Y	Y	Y	N	N	Y	Y
Hua 2009	Y	N	N	N	N	N	N	Y	Y
Karvouni 2000	Y	Y	Y	Y	U/C	N	Y	N	U/C
Malhotra 2018	N	Y	N	Y	Y	U/C	Y	Y	Y
Margey 2011	Y	Y	Y	Y	Y	Y	Y	Y	N
Maron 2016	Y	Y	Y	Y	Y	U/C	U/C	Y	Y
Maron 2014	N	Y	Y	Y	N	U/C	Y	Y	N
Morentin 2001	Y	Y	Y	Y	Y	N	Y	Y	Y
Morentin 2011	N	Y	N	Y	Y	Y	U/C	N	Y
Neuspiel 1985	Y	Y	Y	Y	Y	N	N	N	Y
Papadakis 2009	Y	Y	Y	Y	Y	N	Y	Y	Y
Perez 1992	Y	Y	N	Y	Y	N	U/C	N	Y
Pilmer 2013	Y	Y	Y	Y	U/C	N	Y	N	U/C
Pilmer 2014	Y	Y	Y	Y	Y	N	Y	N	Y
Uuskula 1998	N	Y	Y	Y	Y	N	U/C	Y	U/C
Vaartjes 2009	Y	Y	Y	Y	Y	N	Y	Y	Y
Winkel 2017	Y	Y	Y	Y	Y	N	N	Y	Y
Wisten 2002	Y	Y	Y	Y	Y	Y	N	N	Y
Wisten 2017	Y	Y	Y	Y	Y	N	N	Y	N
Wren 2000	Y	Y	Y	Y	Y	N	Y	Y	Y
Zhang 2019	Y	N	Y	N	Y	N	Y	N	Y

Key: Y- Yes; N- No; U/C- Unclear

Full list of questions:

1. Was the sample frame appropriate to address the target population?
2.Were study participants sampled in an appropriate way?
3.Was the sample size adequate?
4.Were the study subjects and the setting described in detail?
5.Was the data analysis conducted with sufficient coverage of the identified sample?
6.Were valid methods used for the identification of the condition?
7.Was the condition measured in a standard, reliable way for all participants?
8.Was there appropriate statistical analysis?
9.Was the response rate adequate, and if not, was the low response rate managed appropriately?

For peer review only - http://bmjopen.bmj.com/site/about/guidelines.xhtml

Figure S1: Sudden cardiac death incidence by age

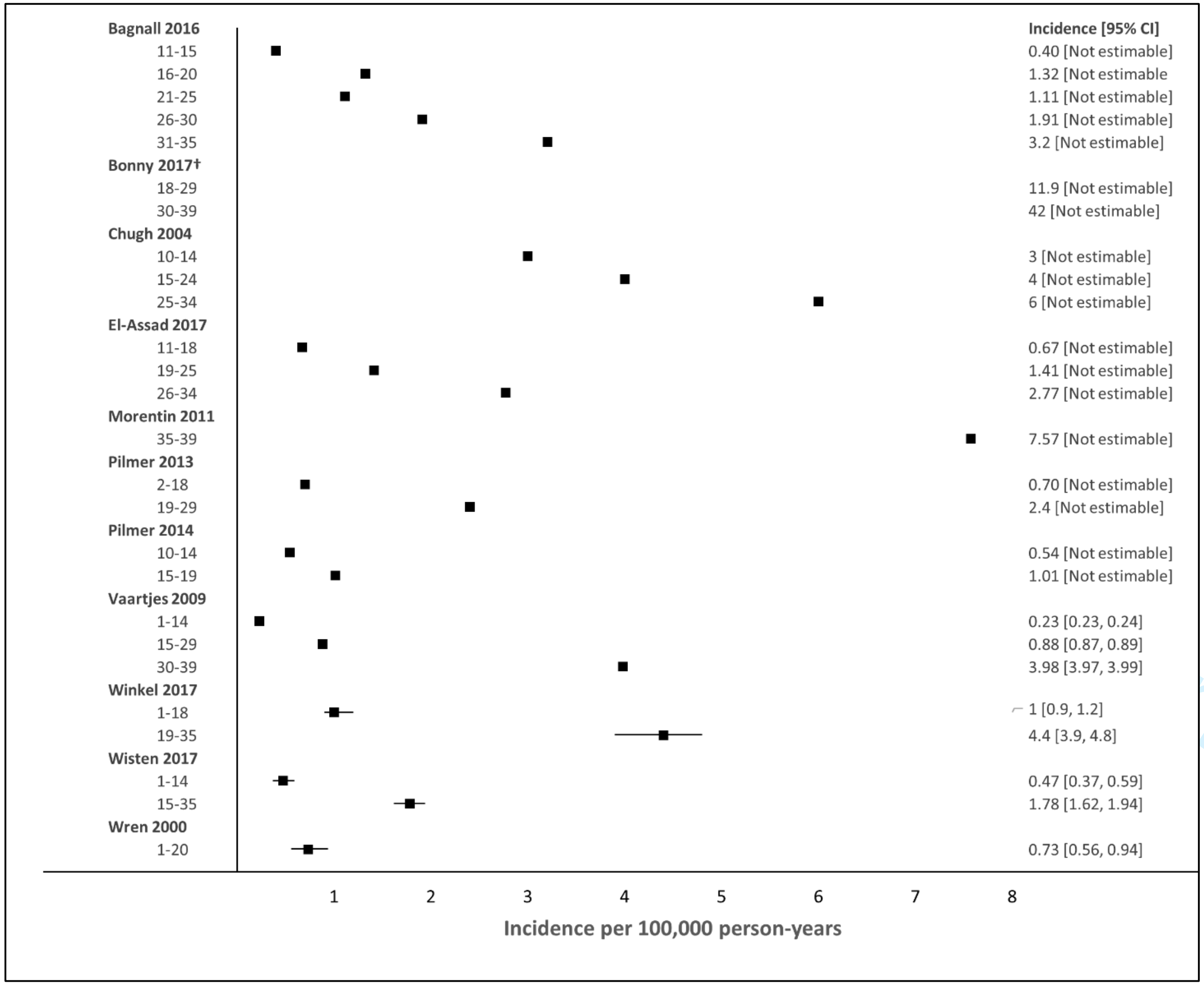

†- Data not presented on chart as significant outlier

For peer review only - http://bmjopen.bmj.com/site/about/guidelines.xhtml

Figure S2: Sudden cardiac death incidence by athletic/ military status

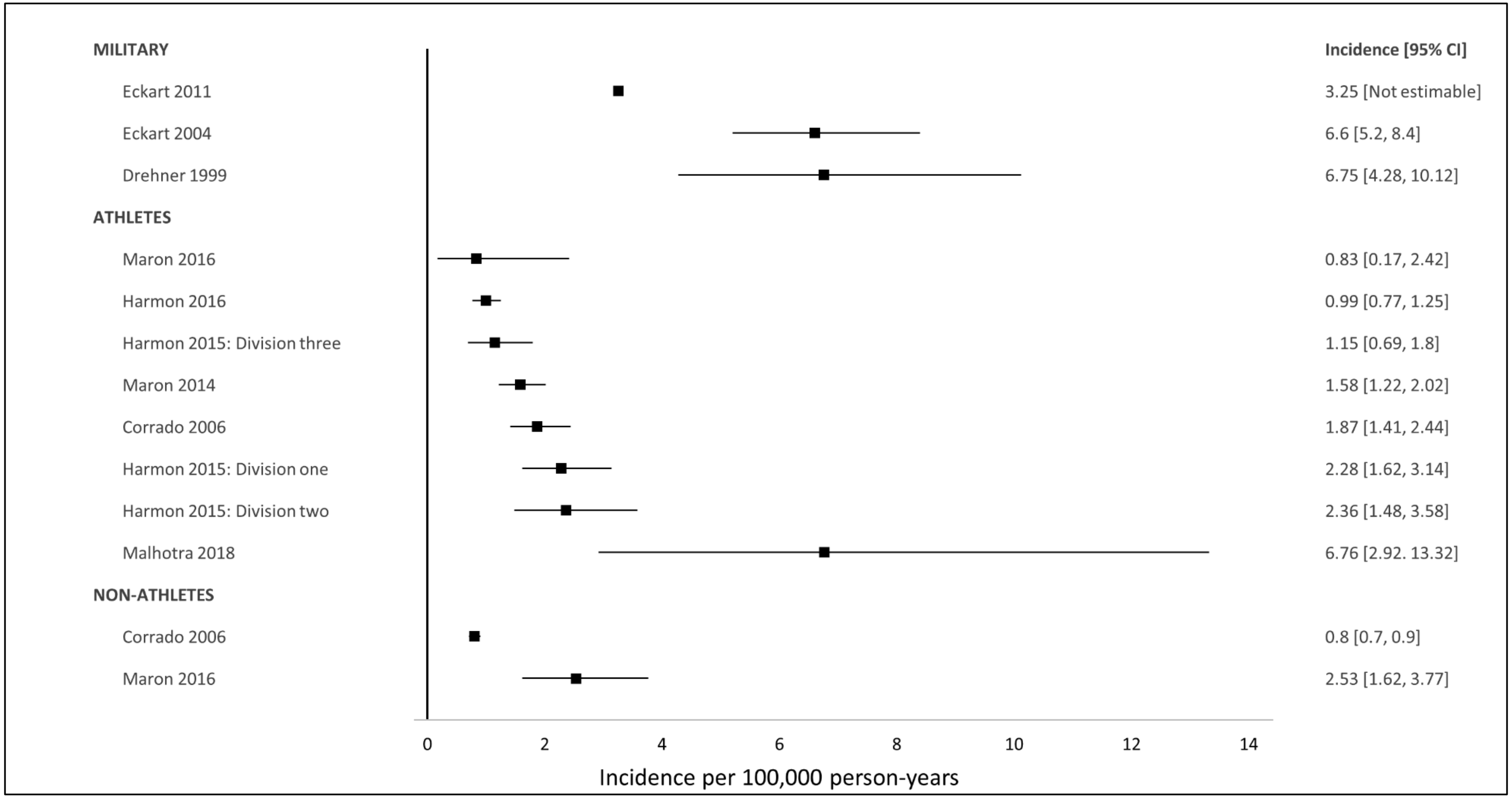

Figure S3: Sudden cardiac death incidence by race

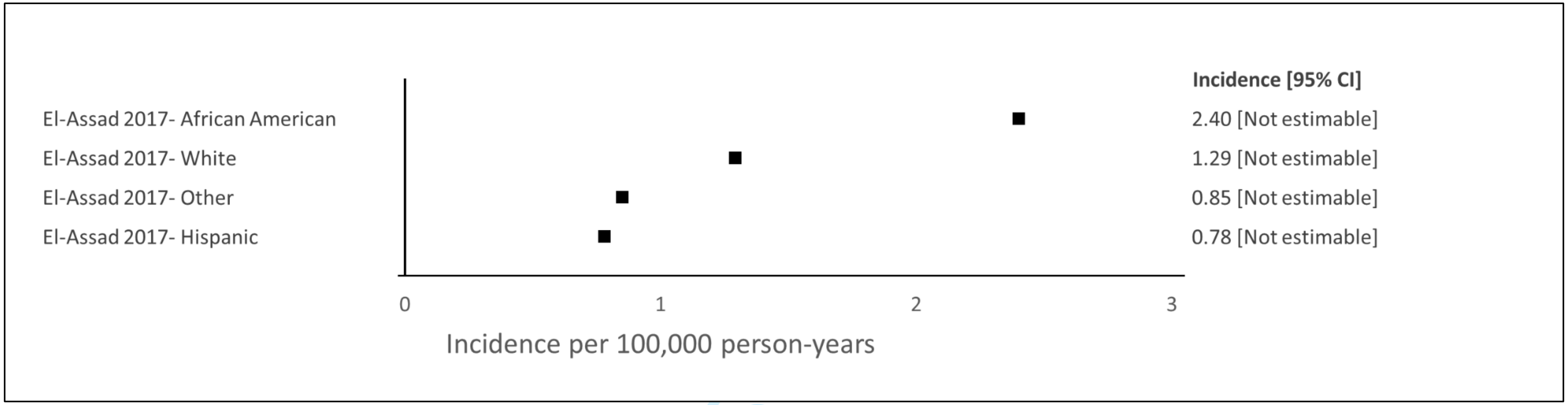

For peer review only - http://bmjopen.bmj.com/site/about/guidelines.xhtml

PRISMA 2009 Checklist

Section/topic	#	Checklist item	Reported on page #
**TITLE**			
Title	1	Identify the report as a systematic review, meta-analysis, or both.	1
**ABSTRACT**			
Structured summary	2	Provide a structured summary including, as applicable: background; objectives; data sources; study eligibility criteria, participants, and interventions; study appraisal and synthesis methods; results; limitations; conclusions and implications of key findings; systematic review registration number.	2
**INTRODUCTION**			
Rationale	3	Describe the rationale for the review in the context of what is already known.	4
Objectives	4	Provide an explicit statement of questions being addressed with reference to participants, interventions, comparisons, outcomes, and study design (PICOS).	4
**METHODS**			
Protocol and registration	5	Indicate if a review protocol exists, if and where it can be accessed (e.g., Web address), and, if available, provide registration information including registration number.	4
Eligibility criteria	6	Specify study characteristics (e.g., PICOS, length of follow-up) and report characteristics (e.g., years considered, language, publication status) used as criteria for eligibility, giving rationale.	5
Information sources	7	Describe all information sources (e.g., databases with dates of coverage, contact with study authors to identify additional studies) in the search and date last searched.	4
Search	8	Present full electronic search strategy for at least one database, including any limits used, such that it could be repeated.	Electronic supplement
Study selection	9	State the process for selecting studies (i.e., screening, eligibility, included in systematic review, and, if applicable, included in the meta-analysis).	4
Data collection process	10	Describe method of data extraction from reports (e.g., piloted forms, independently, in duplicate) and any processes for obtaining and confirming data from investigators.	5
Data items	11	List and define all variables for which data were sought (e.g., PICOS, funding sources) and any assumptions and simplifications made.	5
Risk of bias in individual studies	12	Describe methods used for assessing risk of bias of individual studies (including specification of whether this was done at the study or outcome level), and how this information is to be used in any data synthesis.	5
Summary measures	13	State the principal summary measures (e.g., risk ratio, difference in means).	5
Synthesis of results	14	Describe the methods of handling data and combining results of studies, if done, including measures of consistency (e.g., I^2) for each meta-analysis.	N/A

For peer review only

PRISMA 2009 Checklist

Section/topic	#	Checklist item	Reported on page #
Risk of bias across studies	15	Specify any assessment of risk of bias that may affect the cumulative evidence (e.g., publication bias, selective reporting within studies).	N/A
Additional analyses	16	Describe methods of additional analyses (e.g., sensitivity or subgroup analyses, meta-regression), if done, indicating which were pre-specified.	5
**RESULTS**			
Study selection	17	Give numbers of studies screened, assessed for eligibility, and included in the review, with reasons for exclusions at each stage, ideally with a flow diagram.	5-6/ figure one
Study characteristics	18	For each study, present characteristics for which data were extracted (e.g., study size, PICOS, follow-up period) and provide the citations.	Table one
Risk of bias within studies	19	Present data on risk of bias of each study and, if available, any outcome level assessment (see item 12).	Figure two/ electronic supplement
Results of individual studies	20	For all outcomes considered (benefits or harms), present, for each study: (a) simple summary data for each intervention group (b) effect estimates and confidence intervals, ideally with a forest plot.	Figure 3
Synthesis of results	21	Present results of each meta-analysis done, including confidence intervals and measures of consistency.	N/A
Risk of bias across studies	22	Present results of any assessment of risk of bias across studies (see Item 15).	N/A
Additional analysis	23	Give results of additional analyses, if done (e.g., sensitivity or subgroup analyses, meta-regression [see Item 16]).	Figure 4/ electronic supplemet
**DISCUSSION**			
Summary of evidence	24	Summarize the main findings including the strength of evidence for each main outcome; consider their relevance to key groups (e.g., healthcare providers, users, and policy makers).	7-8
Limitations	25	Discuss limitations at study and outcome level (e.g., risk of bias), and at review-level (e.g., incomplete retrieval of identified research, reporting bias).	7
Conclusions	26	Provide a general interpretation of the results in the context of other evidence, and implications for future research.	7-8
**FUNDING**			
Funding	27	Describe sources of funding for the systematic review and other support (e.g., supply of data); role of funders for the systematic review.	10

For peer review only

PRISMA 2009 Checklist

From: Moher D, Liberati A, Tetzlaff J, Altman DG, The PRISMA Group (2009). Preferred Reporting Items for Systematic Reviews and Meta-Analyses: The PRISMA Statement. PLoS Med 6(7): e1000097. doi:10.1371/journal.pmed1000097

For more information, visit: **www.prisma-statement.org**.

For peer review only

For peer review only - http://bmjopen.bmj.com/site/about/guidelines.xhtml
